# Synthesizing the Evidence Base to Enhance Coordination between Humanitarian Mine Action and Emergency Care for Casualties of Explosive Ordnance and Explosive Weapons: A Scoping Review

**DOI:** 10.1017/S1049023X24000669

**Published:** 2024-12

**Authors:** Hannah Wild, Christopher LeBoa, Nikolaos Markou-Pappas, Micah Trautwein, Loren Persi, Christelle Loupforest, Elke Hottentot, Emilie Calvello Hynes, Jack Denny, Firoz Alizada, Reykhan Muminova, Teresa Jewell, Sebastian Kasack, Stacey Pizzino, Gregory Hynes, Lina Echeverri, Flavio Salio, Sherry M. Wren, Charles Mock, Adam L. Kushner, Barclay T. Stewart

**Affiliations:** 1. Department of Surgery, University of Washington, Seattle, Washington USA; 2. Explosive Weapons Trauma Care Collective, International Blast Injury Research Network, University of Southampton, Southampton, United Kingdom; 3. Department of Environmental Health Sciences, University of California Berkeley, Berkeley, California USA; 4. Center for Research and Training in Disaster Medicine, Humanitarian Aid and Global Health (CRIMEDIM), Novara, Italy; 5. Dartmouth Geisel School of Medicine, Hanover, New Hampshire USA; 6. Victim Assistance Specialist, Belgrade, Serbia; 7. United Nations Mine Action Service (UNMAS), Geneva, Switzerland; 8. Victim Assistance Specialist, Geneva, Switzerland; 9. World Health Organization, Geneva, Switzerland; 10. International Blast Injury Research Network (IBRN), University of Southampton, Southampton, United Kingdom; 11. Antipersonnel Mine Ban Convention Implementation Support Unit, Geneva, Switzerland; 12. Tajikistan National Mine Action Centre, Dushanbe, Tajikistan; 13. Health Science Library, University of Washington, Seattle, Washington USA; 14. Mines Advisory Group, Manchester, United Kingdom; 15. School of Public Health, Faculty of Medicine, The University of Queensland, Brisbane, Australia; 16. International Federation of Red Cross and Red Crescent Societies, Geneva, Switzerland; 17. Stanford University School of Medicine, Stanford, California USA; 18. Surgeons OverSeas, New York, New York USA; 19. Global Injury Control Section, Harborview Injury Prevention Washington and Research Center, Seattle, Washington USA

**Keywords:** blast injury, civilian casualties, conflict, emergency, critical, and operative (ECO) care, explosive ordnance, explosive weapons, low- and middle-income countries, low-resource settings, prehospital trauma care, trauma systems, trauma training

## Abstract

**Background:**

Humanitarian mine action (HMA) stakeholders have an organized presence with well-resourced medical capability in many conflict and post-conflict settings. Humanitarian mine action has the potential to positively augment local trauma care capacity for civilian casualties of explosive ordnance (EO) and explosive weapons (EWs). Yet at present, few strategies exist for coordinated engagement between HMA and the health sector to support emergency care system strengthening to improve outcomes among EO/EW casualties.

**Methods:**

A scoping literature review was conducted to identify records that described trauma care interventions pertinent to civilian casualties of EO/EW in resource-constrained settings using structured searches of indexed databases and grey literature. A 2017 World Health Organization (WHO) review on trauma systems components in low- and middle-income countries (LMICs) was updated with additional eligible reports describing trauma care interventions in LMICs or among civilian casualties of EO/EWs after 2001.

**Results:**

A total of 14,195 non-duplicative records were retrieved, of which 48 reports met eligibility criteria. Seventy-four reports from the 2017 WHO review and 16 reports identified from reference lists yielded 138 reports describing interventions in 47 countries. Intervention efficacy was assessed using heterogenous measures ranging from trainee satisfaction to patient outcomes; only 39 reported mortality differences. Interventions that could feasibly be supported by HMA stakeholders were synthesized into a bundle of opportunities for HMA engagement designated links in a Civilian Casualty Care Chain (C-CCC).

**Conclusions:**

This review identified trauma care interventions with the potential to reduce mortality and disability among civilian EO/EW casualties that could be feasibly supported by HMA stakeholders. In partnership with local and multi-lateral health authorities, HMA can leverage their medical capabilities and expertise to strengthen emergency care capacity to improve trauma outcomes in settings affected by EO/EWs.

## Introduction

Over the past two decades, humanitarian mine action (HMA) stakeholders have devoted immense effort and resources to mitigating civilian harm from explosive ordnance (EO) including landmines, cluster munitions, explosive remnants of war, and victim-activated improvised explosive devices (IEDs).^1^ In contemporary conflicts, use of all types of explosive weapons (EWs), including not only EO but also air-dropped munitions such as bombing and shelling, in densely populated areas causes disproportionate civilian casualties, threatening to overshadow gains made in EO clearance and disposal.^2,3^ For example, the highest number of cluster munition casualties was recorded in 2022 since the Convention on Cluster Munitions (Geneva, Switzerland) entered into force in 2008. This trend was largely driven by the extensive use of cluster munitions in residential areas of Ukraine, including attacks on protected civilian structures (eg, schools and hospitals).^4^ The on-going use of EWs in current conflicts will result in unquantified future casualties before the conclusion of the war and afterwards due to explosive remnants.^5–8^ In Gaza, nearly 18,500 Palestinians were injured and over 7,000 killed (66% women and children) between October 7 and the October 27, 2023 ground invasion by the Israel Defense Forces, nearly all of which were likely attributable to explosive-injury-related deaths from bombings and other air-launched munitions.^9^

Humanitarian mine action refers to a set of activities and initiatives aimed at addressing the impact of EO on civilian populations and communities. These activities aim to minimize the threat posed by EO, allowing affected areas to be safely accessed and used for agriculture, housing, and access to services as well as to meet the needs of people injured, survivors, affected family, and community members. Largely, HMA is comprised of five complementary pillars: (1) EO risk education (EORE); (2) land release (ie, survey, mapping, marking, and clearance); (3) victim assistance (ie, emergency and on-going medical care, rehabilitation, psycho-social support, and socio-economic inclusion); (4) stockpile destruction; and (5) advocacy against the use of EO that are prohibited, indiscriminate, and/or cause disproportionate civilian harm.^10^ The HMA sector is diverse and dynamic, with a variety of actors and structures collaborating at the global level and in EO-affected countries, including National Mine Action Authorities (Geneva, Switzerland) and Centers, HMA operators, survivors’ organizations, countries engaged in international cooperation and assistance, United Nations treaty (eg, Antipersonnel Mine Ban Convention [APMBC; convention on the prohibition of the use, stockpiling, production, and transfer of anti-personnel mines and their destruction] and Convention on Cluster Munitions), implementation support units, as well as public, private, and nongovernmental organizations (NGOs) providing the services included in victim assistance. The HMA stakeholders have an organized presence with significant medical capabilities in many conflict and post-conflict settings where local health infrastructure is often disrupted and humanitarian health actors are sparse. As such, HMA is uniquely positioned to contribute to strengthening emergency care systems to improve trauma care quality for civilian casualties of EO. Though HMA focuses predominantly on EO, such engagement would also yield benefits for casualties of EWs. Yet at present, few mechanisms for such care and coordination exist.

In November 2022, 83 countries adopted the *Political Declaration on Strengthening the Protection of Civilians from the Humanitarian Consequences Arising from the Use of Explosive Weapons in Populated Areas* (EWIPA).^11^ Elsewhere, survivors of landmines and other EO/EWs are leading their communities to recovery in extraordinary examples of grassroots advocacy and action.^12^ Continued prevention, clearance, and advocacy initiatives are necessary but insufficient given continued civilian casualties of EO/EWs. Many EO/EW incidents occur in low-resource settings, where post-injury care must be significantly strengthened to reduce death and disability (Table 1 ^13,14^). Organized emergency care systems have demonstrated positive impact on preventable mortality, yet are challenging to organize given resource and security constraints in conflict-affected settings.^15–19^ A scoping review was conducted with the objective of identifying both evidence-based and feasible emergency care interventions that HMA stakeholders could engage in to support civilian casualties of EO/EWs. The synthesis of these interventions can help inform the development of a strategy for enhanced care and coordination between HMA and health stakeholders including Ministries of Health and the World Health Organization (WHO; Geneva, Switzerland) to improve outcomes for EO/EW casualties.

**Table 1.** Explosive Ordnance Casualties and Health Care/Surgical Capacity by Country

Countries with >500 Mine/Explosive Remnant of War Casualties in 2022	EO Casualties	Hospital Beds per 1,000 Population	Surgeons per 100,000 Population
Syria	834	1.4	[NA]
Ukraine*	608	7.5	87
Yemen*	582	0.7	1
Myanmar	545	1.0	2
Nigeria	431	0.5	1
Afghanistan	303	0.4	0
Mali	182	0.1	1
Iraq	169	1.3	8
Colombia	145	1.7	23
Angola	107	0.8	[NA]

Note: From Landmine Monitor 2023^13^ and World Bank Data^14^; it is important to note that the burden of EO is not known in many affected contexts due to inadequate casualty surveillance capacity. Further, IEDs are not fully captured in these data.

* Indicates State Parties to the Antipersonnel Mine Ban Convention.

Abbreviations: EO, explosive ordnance; IED, improvised explosive devices.

## Report: Methods

### Search Strategy

A scoping review was conducted of peer-reviewed and grey literature repositories to identify records describing trauma care interventions with feasible resource requirements applicable to civilian casualties of EO/EWs. Scoping review methodology was used given the heterogeneity of included literature as well as the specific aims of this review (ie, synthesizing existing evidence to derive opportunities for improved coordination between health and HMA stakeholders). A 2017 WHO review evaluating the impact of trauma systems and system components in low- and lower-middle income countries (LMICs) was updated during this review.^20^ Interventions with demonstrated efficacy in LMICs may have relevance to the environments in which EO/EW casualties occur, many of which are resource-constrained, and have the potential to be adapted for implementation in conflict settings. Additional search strings were constructed using keywords and database-specific index terminology to include interventions relevant to casualties of EO/EW (Supplement 1; available online only). Search terms for LMICs were developed from the Cochrane Effective Practice and Organization of Care (EPOC; London, United Kingdom) LMIC filters 2020 (v.4). A pre-review protocol was registered with Open Science Framework (Charlottesville, Virginia USA; Supplement 2 - available online only).^21^ Database searches were conducted in PubMed/MEDLINE (National Center for Biotechnology Information, National Institutes of Health; Bethesda, Maryland USA); Embase (Elsevier; Amsterdam, Netherlands); Cumulative Index to Nursing and Allied Health Literature (CINAHL) on EBSCO (Ipswich, Massachusetts USA); Global Index Medicus (WHO); CABI Global Health (EBSCO); Cochrane Library (Wiley; Hoboken, New Jersey USA); Web of Science Core Collection (Clarivate Analytics; London, United Kingdom) – SCI-EXPANDED, SSCI, AHCI, ESCI; and Google Scholar (Google Inc.; Mountain View, California USA), as well as grey literature including organizational websites (eg, WHO and International Committee of the Red Cross [ICRC; Geneva, Switzerland]). Reference lists of eligible reports were screened for relevant records.

### Eligibility Criteria

All reports from the 2017 WHO review were included in analysis.^20^ Eligibility criteria limited additional reports to those that described trauma care interventions in LMICs or civilian casualties of EO/EWs. The definition for EO was in accordance with International Mine Action Standard (IMAS; Geneva, Switzerland) 4.10, and EW was used to describe all forms of weapons causing explosive injuries including air-dropped munitions.^22^ Eligible interventions and patient populations were limited to those from LMICs as defined by The World Bank (Washington, DC USA) economic classification (economic classification at study time point was utilized – for example, a record published in 2002 was evaluated for country income class at year of publication) or settings of active violence in non-high-income countries including international armed conflict, non-international armed conflict, and other armed violence.^23^ Reports describing injuries of civilians or local non-NATO (North Atlantic Treaty Organization; Brussels, Belgium) coalition combatants were eligible for inclusion, consistent with previous comprehensive reports on this topic, as both populations are unlikely to be protected by body armor and may experience similar injury patterns.^3,24^ Reports describing care rendered to military service members of high-income countries were not eligible. Due to limited data on the topic of interest, no studies were excluded based on study design, assessment of data quality, or risk of potential bias in keeping with WHO Rapid Review procedures.^25^ Date restrictions limited results to those published in or after the year 2000 to maintain relevance to modern conflict dynamics coinciding with the onset of the United States Global War on Terror in Afghanistan in 2001. Reports exclusively describing mental health and late rehabilitation interventions were excluded. Cross-sectional reports on injury epidemiology that did not describe an intervention were excluded. No language restrictions were applied. Google Translate was used for non-English language reports in which authors were not proficient.

### Data Management

**Figure 1.**
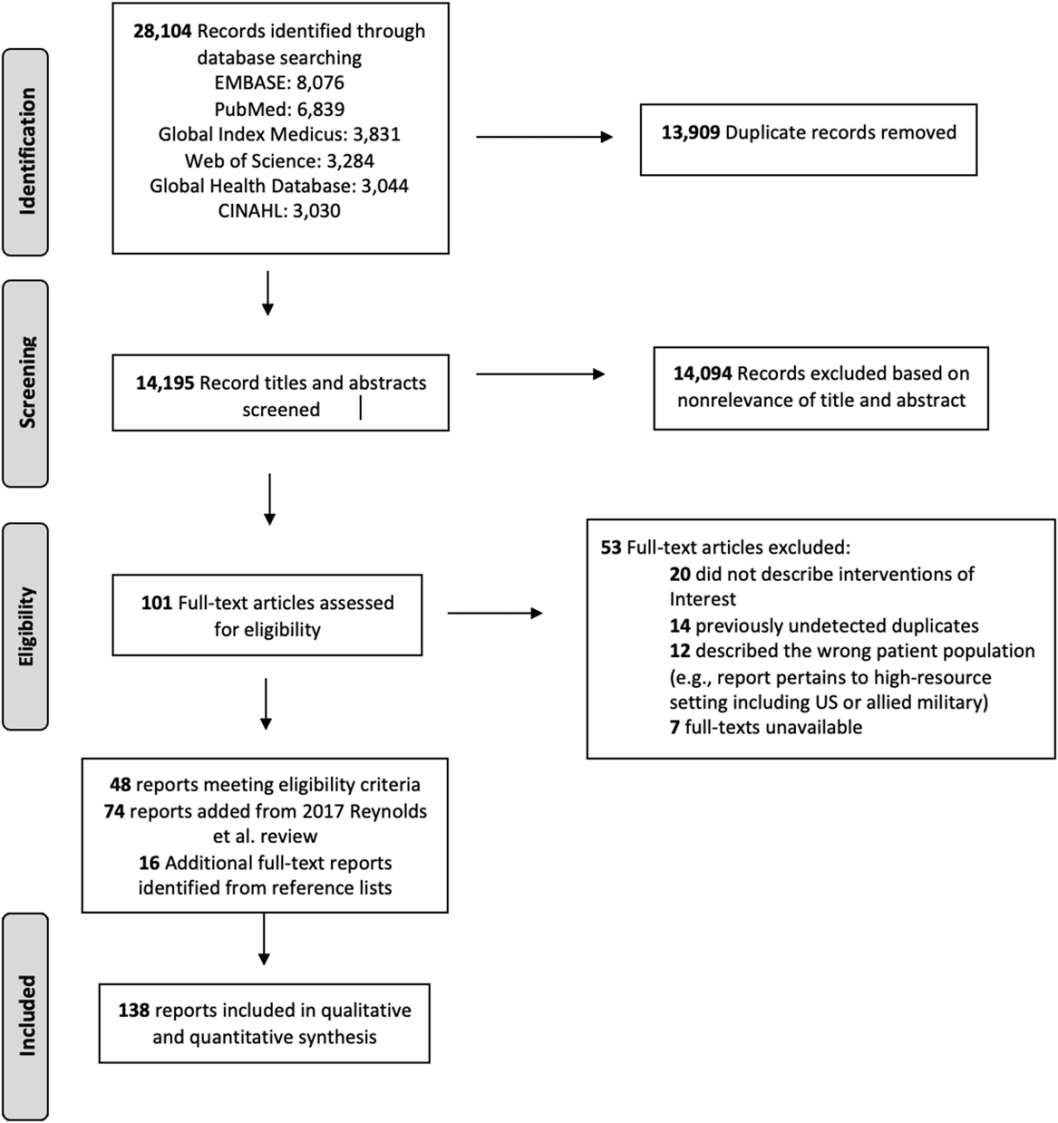
PRISMA-ScR Diagram of Selected Reports.

Records were maintained using Covidence Review Software (Veritas Health Innovation; Melbourne, Australia). Two independent reviewers screened all records identified by title and abstract to determine relevance to eligibility criteria. A senior reviewer arbitrated discrepancies. Full-text reports of eligible records were retrieved and screened by two independent reviewers. Preferred Reporting Items for Systematic Reviews and Meta-Analyses Extension for Scoping Reviews (PRISMA-ScR) methodology was followed and search results were reported accordingly (Figure 1; Supplement 3 - available online only).^26^

### Data Extraction and Analysis

A standardized extraction database was developed to capture elements including study information (eg, year, location, and study design); intervention details (eg, target population, duration, format, and resource requirements); and outcome measures (eg, mortality and clinical performance improvement). Given the range of methodologies and metrics of intervention efficacy presented, structured qualitative analysis of reports was conducted and data were synthesized in narrative format.^27^ Study quality was assessed using the Quality Assessment with Diverse Studies (QUADS) score (Supplement 4; available online only).^28^

### Intervention Synthesis and Coordination Opportunities

To present data in a manner harmonized with the WHO Emergency Care System Framework (ECSF), interventions were categorized by phase of care: (1) layperson first response (LFR - community members without formal medical training; ECSF “scene”), (2) prehospital (providers with medical training providing care in the prehospital setting; ESCF “transport”), and (3) facility-based (trauma care rendered by providers in a health facility; ESCF “facility”).^29^ Formal framework synthesis methodology such as Best Fit Framework Synthesis was not adopted due to a lack of appropriate candidates for pre-existing models against which to code. Specifically, data could not be coded against existing emergency care frameworks, including the WHO ECSF, as these did not take into account the need to encompass only those interventions that could be feasibly supported by HMA stakeholders through existing operations. Two key interventions were selected from each phase that: (1) had been successfully implemented in resource-constrained settings, (2) possessed potential to reduce trauma-related mortality among EO/EW casualties, (3) had high or very-high level quality evidence as assessed by QUADS score, and (4) could feasibly be supported by HMA stakeholders. Criteria (1)-(3) were evaluated through standard scoping review methodology while criterion (4) was evaluated qualitatively by coauthors within the HMA sector and through a separate process of semi-structured interviews with HMA sector experts.^30^ In domains where more than two interventions met all of the above criteria, priority was given to those with the most robust QUADS scores or those described in multiple reports, as well as compatibility with integration in existing HMA activities. These interventions were then synthesized into an over-arching series of potential opportunities for HMA stakeholders to engage in emergency system strengthening to improve trauma care for civilian EO/EW casualties in a manner consistent with the WHO ECSF and Trauma Pathway.^31^ These interventions were designated as links in a Civilian Casualty Care Chain (C-CCC).

## Report: Results

### Search Results

A total of 28,104 records were identified by this search strategy with 13,909 duplicate records excluded (Figure 1). The remaining 14,195 records were screened for relevance, 14,094 of which were excluded. Full-text reports of the remaining 101 records were evaluated with 53 reports excluded: 20 did not describe interventions of interest, 14 were previously undetected duplicates, 12 did not describe the patient population of interest, and seven full-texts were unavailable. Forty-eight reports met eligibility criteria. The 2017 WHO review contained 74 additional reports. Sixteen additional reports were identified from reference lists of eligible reports. In total, 138 reports met eligibility criteria and were included in analysis. Quality of evidence varied widely as assessed by QUADS criteria, with 25 reports qualitatively evaluated as very low, 17 as low, 50 as moderate, 27 as high, and 19 very high (Supplement 3; available online only).^28^

### Geographic Distribution

**Figure 2.**
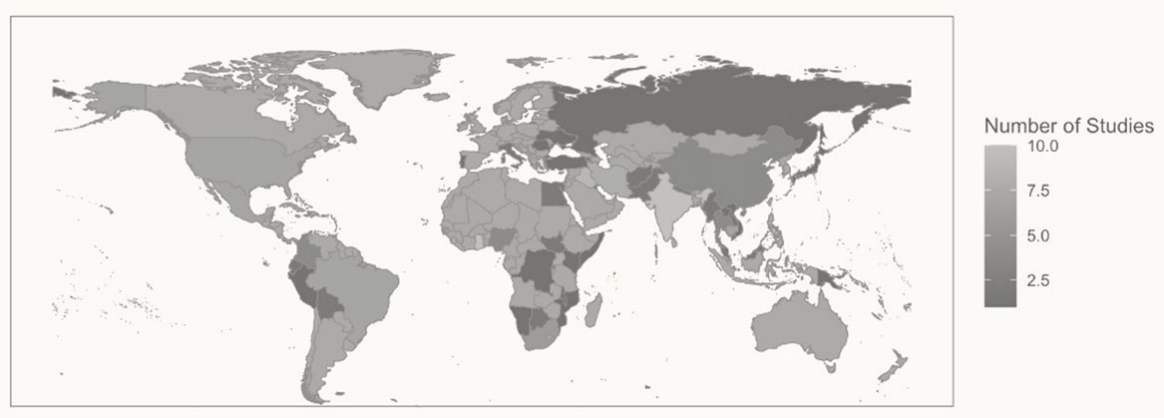
Geographic Distribution of Interventions Included in Analysis.

Interventions from 47 countries were described (Figure 2). The most frequent countries were Ghana and India (n = 10 each), Iraq (n = 9), Iran and Uganda (n = 8 each), and Brazil, Cambodia, and Mexico (n = 7 each). Remaining reports were broadly distributed including 45 in Africa, 36 in Asia, 23 in Latin America, and 22 in the Middle East. Fifteen reports represented interventions deployed in multiple countries not adequately specified for disaggregation.

### Intervention Types and Phase of Care

Reports included in analysis described 40 LFR interventions, 35 prehospital interventions, 62 facility-based interventions, and one that could not be categorized. Trauma care training courses (TCTCs) were the dominant intervention type (n = 84). For LFR, TCTCs were most frequent (n = 36; eg, emergency first aid training provided to commercial drivers in Ghana and Nigeria),^32,33^ followed by layperson transport systems (n = 2). For prehospital interventions, TCTCs targeting prehospital personnel were most frequent (n = 18; eg, trauma training for prehospital personnel in landmine-contaminated regions of Iraq and Cambodia),^34^ followed by Emergency Medical Services (EMS) coordination (n = 12; eg, notification systems to alert receiving facility of incoming patients).^35^ Facility-based interventions included TCTCs for health facility clinical personnel (n = 30), trauma systems organization initiatives (n = 17; eg, establishment of designated trauma teams with standardized roles in Pakistan),^36,37^ and hospital-based clinical care protocols (n = 13; eg, standardized trauma protocols for management of patients with traumatic brain injury [TBI] in Colombia).^38^ Cost effectiveness and data collection were the primary focus of six and three reports, respectively.^39–47^ Interventions focused on pediatric trauma and burn care were described by only two reports each.^48–51^ Intervention details are highlighted in Table 2 ^32–36,38,52–66^ and summarized completely in Supplement 3.

**Table 2.** Selected Interventions by Phase of Care

Intervention Type	**Example Reports** **(Author, Year)**	Target Population	Intervention Description	Intervention Duration	Intervention Format	Intervention Content	Intervention Outcomes
**Phase 1: Layperson First Response**							
Layperson First Responder Training	Capone 2000^56^	Factory workers in Brazil	LFSA was taught to factory employees in Brazil via television demonstrations	7-minute video on LFSA was used to train factory works in first aid skills; post-course skills evaluations occurred at 1 week, 1 month, and 13 months	Televised video on commercial network with simulated skill assessment on manikins	Hemorrhage control; fracture immobilization; burn treatment; victim positioning; airway management; CPR (steps A–B–C)	A statistically significant increase in skills performance was observed among television trainee group compared to control, with over 50% performing over 5/8 skills (eg, hemorrhage control, patient positioning) correctly
	Mock 2002^32^	Commercial drivers in Accra, Kumasi, and Brong-Ahafo, Ghana	First-aid training course for commercial drivers in Ghana was administered; instructors were physicians, nurses, and local Ghana Red Cross first aid instructors	6-hour basic first aid course was designed for commercial drivers assuming a low-level of education	In-person didactics with a combination of lectures and hands-on skills sessions	Course content included scene management, hemorrhage control, airway management, splinting/fracture immobilization, basic spinal precautions (not including cervical collars/backboards), triage	Improvement in first aid included: scene management (7% pre- vs 35% post-), airway management (2% vs 35%), hemorrhage control (4% vs 42%), and splinting of injured extremities (1 vs 16%)
	Murad 2012^59^	Lay-person trauma first responders and paramedics in villages in Iraq	Basic Life Support training was provided to villagers and paramedics in regions of Iraq affected by conflict and landmine contamination	Village first responders were trained in 2-day courses with 1-day rehearsal courses after 6-8 months	In-person didactics; training curriculum emphasized local real-life case stories and hands-on training	Content included patient positioning, transport/evacuation, hemorrhage control, airway management, splinting/fracture immobilization, prevention of hypothermia	The outcomes of patients initially managed in-field by first responders prior to paramedic arrival were compared to patients not receiving first responder support: prehospital care reduced the mortality rate to 8% in the treatment group compared to 44% in the control group
	Olumide 2015^33^	Commercial drivers in Nigeria	First aid training was taught to commercial drivers in Nigeria	2 days	In-person training; mixed didactics and practical exercises	Course covered life-saving emergency principles including: scene safety, transport and evacuation, initial victim assessment/rapid survey, hemorrhage control, fracture management, and HIV prevention; course materials were adapted from the BTLS manual	The effect of first aid training on the first aid knowledge and skills of participants was assessed with scenarios: first aid knowledge scores improved from 49% to 59% pre- and post-course, and skills sores improved from 17.5% to 81%
Layperson Transport Systems	Sharma 2013^57^	Motorcycle owner volunteers in Nepal	Volunteer transport system for snake bite victims combined with community health education in rural Nepal was assessed	Not specified	Program relied on network of motorcycle owners who volunteered to transport snake bite victims; snake bite awareness sessions were conducted in each village development community	Course content covered snake bite awareness including prevention, immediate care, and victim transport	The case-fatality rate was reduced from 11% to <1% post-intervention
	Shehu 1997^58^	Private commercial drivers in Nigeria	Emergency transport network for women with obstetric complications in Nigeria was established employing commercial drivers in combination with training	Not specified	Network of private commercial drivers was established to transport women with obstetric emergencies to a health facility; emergency fuel fund was established to cover costs	Course curriculum covered a range of obstetric complications with didactic and practical components and addressed behavioral aspects to make these services culturally acceptable	Time from onset of labor to treatment ranged from 3 to 36 hours, with a mean of 9 hours for the 2 years
**Phase 2: Prehospital**							
Prehospital Provider Trauma Training	Arreola-Risa 2007^53^	Prehospital medics in Mexico	EMT certification was provided to all prehospital personnel working for an EMS service in Mexico	14-month EMT certification consisting of 2-3 hours of class daily was provided to students	In-person; mix of didactic lectures and hands-on sessions	Course content included extrication, scene management, spinal immobilization, airway maneuvers, IV fluid resuscitation	Course impact was assessed based on process measures and patient outcomes: mortality decreased from 1.8% to 0.5% after the training; 3-month program cost US$200-600 per medic compared to 2–3-day BTLS courses that are approximately US$150
	Husum 1999^34^, 2003a^54^, 2003b^60^; Wisborg 2008^61^	Local health care workers in Cambodia and Iraq	Low-cost trauma training for non-graduate village health care workers in Cambodia and Northern Iraq was deployed and assessed prospectively over 5 years	Village health care workers were trained with a low-cost ATLS training consisting of 3 150-hour courses held in makeshift training camps with 6-12-month periods between course sessions; between course sessions, health care workers themselves trained layperson village first-responders during 2-day courses	In-person; predominantly hands-on skills sessions and workshops	Students were supplied with a medical backpack containing basic equipment and medications; course content included a wide-range of resuscitative principles around trauma care including hemorrhage control and airway management	Intervention efficacy was assessed based on patient outcomes at nearby referral surgical hospitals and injury severity scores/physiological severity scores; trauma mortality rate was reduced from pre-intervention level at 40% to 15% over the study period
	Saghafinia 2008^62^, 2009^63^	Rural health care workers and community members in Iran	BTLS training was provided to rural health workers and prehospital medics in Iran; trainings were coupled with first-aid courses for laypersons and nomads as well as ALS for nurses and physicians	20-hour course in basic trauma care was provided to rural health care workers and EMTs; coupled with first aid (15-hour for laypersons with a higher degree of education, 12-hour for people with minimal education/high school students, 8-hour brief courses for people with no education and nomadic populations) trauma training for laypersons as well as a 24-hour advanced trauma training for physicians and nurses at the local trauma hospital	In-person; consisting of didactic lectures and hands-on training with animal models	Basic trauma care course covered content including hemorrhage control, CPR, IV access and fluid resuscitation, airway management	Course efficacy was assessed using patient ISS and PSS: significant reduction in mortality was observed pre- and post-training from 7.3% to 3%; mean PSS among patients who received prehospital care was higher (7.4) compared to 5.9 among those who did not
EMS Coordination	Mitra 2020^35^	Prehospital personnel and ED clinicians in India	Prehospital notification system for ambulance personnel to notify EDs of incoming trauma patients was examined	N/A	Mobile phone application was developed for prehospital personnel to alert ED clinicians of incoming trauma casualties	Transmitted data included information on patient demographics, mechanism of injury, and vital signs with embedded GPS data to generate an estimated time of arrival; alert-initiated activation of multi-disciplinary trauma team in the receiving ED	More patients were managed with a trauma notification (RR 1.30; 95% CI, 1.10 to 1.52) and trauma team leader presence (RR 1.50; 95% CI, 1.07 to 2.10); reduced risk of death in the ED (RR 0.11; 95% CI, 0.03 to 0.39).
**Phase 3: Facility-Based**							
Hospital-Based Trauma Trainings	Aboutanos 2007^64^	Rural physicians in Ecuador	Basic trauma care course using local resources was administered to rural physicians in Ecuador	3-day course taught by local surgeons and physicians, some of whom were ATLS instructors	In-person; comprised of didactic lectures and practical skills sessions	Lecture topics included introduction to trauma and EMS (airway management, hemorrhage control, musculoskeletal injuries, thoracoabdominal and pelvic injuries, radiographic diagnoses), triage, burns, and snake bites	Course effectiveness was evaluated by a comparison of pre- and post-course test scores as well as OSCE performance: test scores significantly improved from pre- to post-test (72% to 79%); in rudimentary health posts, 76% of physicians passed, management was adequate for hemorrhage control (65%), immobilization (77%), and early transfer (92%)
	Bertol 2014^55^	MSF clinicians in DRC, Haiti, Afghanistan	Protocol for open fracture management using external fixation was evaluated at MSF surgical programs in 3 countries	1-year quality improvement initiative	Training on the use of external fixation was provided to local national staff by expatriate orthopedic and general surgeons as part of a clinical algorithm for management of open fracture	Protocol included administration of prophylactic antibiotics, irrigation, debridement, and immobilization	Limb salvage rates following these trainings were assessed; program demonstrated a decrease in amputation rates among open fractures from 100% to 21% in the DRC and 20% to <10% in Haiti and Afghanistan
	Kesinger 2014a^38^, 2014b^65^	Patients with severe TBI in Colombia	STP based on best practices in management of TBI was implemented at a Level-1 trauma center in Colombia	N/A	Electronic database was established and implemented to facilitate clinical quality improvement based on an STP for management of TBI	STP was implemented with process measures including blood transfusions, arterial blood gas draws, bladder catheterization, use of hypertonic fluids, use of prophylactic antibiotics, administration of tetanus vaccine, and spinal immobilization	Electronic database was developed to capture clinical data necessary to assess intervention impact using outcomes including mortality, GCS, length of hospital and ICU stay; post-STP in-hospital mortality decreased (38% vs 18%), and discharge GCS increased (median 10 vs 14); odds of in-hospital mortality post- compared to pre-STP were 0.248; ED interventions increased, including bladder catheterization (49% vs 73), hypertonic saline (38% vs 63), arterial blood gas draws (25% vs 43%), and blood transfusions (3% vs 18%)
	Mock 2005^52^	GPs at rural hospitals in Ghana	Trauma course including essential trauma surgery skills was administered to GPs at rural hospitals in Ghana	40-hour long course was designed based on an initial needs assessment	Course focused primarily on hands-on skills stations	Course taught skills including trauma management within the purview of GPs in rural hospitals, recognition of injuries warranting referral, management of such injuries when referral is impossible or delayed; clinical content ranged from primary survey to head and thoracoabdominal trauma, burns, and extremity injury, with procedures including venous cutdown, thoracostomy tube placement, diagnostic peritoneal lavage, cricothyroidotomy, small bowel resection, splenectomy, repair of complex laceration, open fracture management	Test scores improved from 69% to 80% with improvements in all major categories with high level of utilization of basic airway maneuvers (93%) and chest tube insertion (67%)
	Tolppa 2020^66^	Health care workers in Democratic Republic of Congo (DRC)	PTC course was introduced for health care workers in the DRC	PTC is a 2-day course; first course was run by UK volunteers; Congolese participants were subsequently trained as instructors	Course consists of lectures, skills workshops, and case scenarios	Content covers the primary and secondary surveys, airway management, head/spine/chest/abdominal trauma, and shock as well as the management of pediatric and pregnant patients with practical stations on airway and cervical spine management, spinal immobilization, and thoracostomy tube placement	There was an increase of 4.8 in test scores and 9.6 in confidence scores (P < .01) post-course
Trauma Systems Organization	Hashmi 2013^36^	Residents and faculty at the Aga Khan University Hospital in Karachi, Pakistan	Specialized trauma team was formed and trained to conduct trauma resuscitations with designated roles	2-day modified ATLS training for residents and faculty was conducted	Multi-disciplinary trauma team was formed; trauma team protocol activation was developed in conjunction with the creation of a trauma registry and quality improvement program	Fundamental principles of trauma care including hemorrhage control, airway management, resuscitation, and targeted quality improvement initiatives	Injured patients were 4.9 times less likely to die and 2.6 times less likely to have a complication compared with those cared for before the interventions

Note: For complete summary of reports included in analysis, see Supplement 3 (available online).

Abbreviations: ALS, Advanced Life Support; ATLS, Advanced Trauma Life Support; BEST, Better and Systematic Team Training; BTLS, Basic Trauma Life Support; CPR, Cardiopulmonary Resuscitation; DRC, Democratic Republic of Congo; ED, Emergency Department; EFAR, Emergency First Aid Responders; EMS, Emergency Medical Services; EMT, Emergency Medical Technician; EMWT, Emergency Ward Management of Trauma; GCS, Glasgow Coma Scale; GETC, Guidelines for Essential Trauma Care; GP, General Practitioner; GPS, Global Positioning System; ICU, Intensive Care Unit; ISS, Injury Severity Score; HIV, Human Immunodeficiency Virus; IV, Intravenous; LFSA, Life-Supporting First Aid; LMIC, Low- and middle-income country; MSF, Médecins sans Frontières; NGO, Nongovernmental Organization; OSCE, Objective Structured Clinical Examination; PSS, Physiologic Severity Score; SMART, Surgical Management and Reconstruction Training; STaRTLE, Surgical Techniques and Repairs in Trauma for the Low-resource Environment; STP, Standardized Trauma Protocol; TACS, Trauma and Acute Care Surgery; TBI, Traumatic Brain Injury; TFRC, Trauma First Responder Course; US, United States; WHO, World Health Organization.

### Intervention Format and Resource Requirements

The TCTC format and duration ranged widely. Most incorporated both didactic and hands-on skills components. Course duration was one-to-two days in most LFR trainings.^52,67,68^ While some prehospital and facility-based TCTCs were conducted in one-to-two days (eg, specialized flap techniques for reconstruction of soft tissue defects),^69^ others extended over months such as an emergency medical technician (EMT) certification program in Mexico and the highly impactful “Village University” program, which adopted an intensive format with three 150-hour courses.^53,54^ Seven reports presented data on intervention cost effectiveness.^40–44,70,71^

### Outcomes

Heterogenous outcome measures were utilized to assess intervention efficacy. Varied assessment strategies were reported for TCTCs including pre- and post-course skills and knowledge, as well as self-assessed participant confidence. Thirty-nine reports presented mortality as an outcome with variable definitions (eg, mortality in the emergency department [ED] versus in-hospital mortality). Significant reductions in trauma-related mortality were observed for interventions at all phases of care (eg, the “Village University” LFR and prehospital trauma care trainings [40% to 15% post-intervention];^34^ a trauma team activation and prehospital notification intervention in India [relative risk {RR} 0.11 for death in the ED];^35^ and a standardized protocol for management of patients with thoracic trauma in Thailand [25% to approximately 15% post-intervention mortality]).^72^ Five reports presented data on functional outcomes at discharge.^38,55,73–75^ Other outcomes included process measures such as clinical (eg, blood transfusion and procedural interventions) as well as systems measures (eg, prehospital transport times and time to the operating room); resource utilization (eg, length of hospital stay and intensive care unit admission); and complications (eg, surgical site infection and venous thromboembolism).

### Synthesis into a Bundle of Interventions: Civilian Casualty Care Chain (C-CCC)

**Figure 3.**
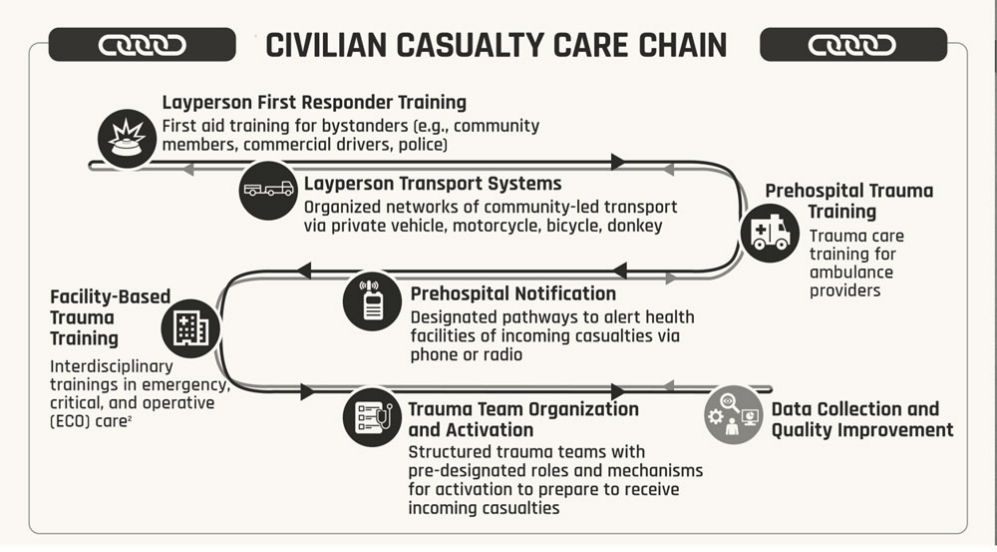
Links in the Civilian Casualty Care Chain (C-CCC) Note: 1. The C-CCC outlines a selected set of interventions that represent opportunities for HMA stakeholders to engage in health sector initiatives to improve emergency care of EW casualties. The C-CCC is not itself an emergency care pathway, as it lacks many of the emergency care system components needed for a continuum of response. Rather, it highlights specific areas of targeted action in which HMA stakeholders might leverage their existing capabilities, infrastructure, and operations to support local emergency care systems to improve trauma care for EW casualties. 2. Interdisciplinary refers to the interprofessional nature of robust ECO care, engaging all relevant health care providers including physicians, surgeons, nurses, and health officers.

At each phase, two key interventions were selected with evidence of successful implementation in resource-constrained settings and potential to reduce mortality that could feasibly be supported by HMA stakeholders through existing operations and capabilities. These interventions were concatenated into a series of opportunities for HMA engagement with emergency care systems strengthening to improve trauma outcomes among EO/EW casualties structured across a continuum of care from point-of-injury to treatment at a health facility. These interventions were designated links in the C-CCC (Figure 3.) By phase of care, interventions included: LFR training and organized layperson casualty transport systems; prehospital TCTCs and notification systems for prehospital providers to alert facilities of incoming casualties; and facility-based TCTCs as well as trauma team organization and activation protocols. The C-CCC does not itself represent an emergency care framework as it lacks many of the elements needed for a complete continuum of response. Rather, this structure highlights selected areas of targeted intervention where HMA stakeholders can leverage their expertise to support health stakeholders with a shared goal of reducing preventable death and disability among EO/EW casualties.

## Discussion

In this review, identified were trauma interventions conducted in LMICs with applicability to the care of civilian EO/EW casualties in resource-constrained settings. The objective was to synthesize the literature on existing interventions to inform enhanced coordination between HMA and health stakeholders to strengthen emergency care systems to improve trauma outcomes among EO/EW casualties. This analysis demonstrates a heterogenous but comprehensive evidence base for interventions at each phase of care that can feasibly be supported through existing HMA capabilities and operations. Main findings included: (1) a high degree of variability in format, quality of monitoring, and evaluation strategies/outcomes reported within each intervention type; (2) significant gaps in the domains of pediatrics and burns, both highly relevant to the context of blast injury; and (3) opportunities exist for HMA stakeholders to engage in numerous interventions across a continuum of care from point-of-injury to treatment at a health facility in a manner supportive of existing frameworks such as the WHO ECSF and Trauma Pathway (Table 3).

**Table 3.** Implementation Strategies and Research Gaps

Phase of Care	Intervention	Implementation Opportunities	Research Gaps	WHO Emergency Care System Framework
Layperson First Response	Layperson Transport	Utilize EORE community liaisons to identify context-specific opportunities for the coordination of layperson transport networks to decrease time from point-of-injury to point-of-contact with trained provider	Sustainable funding models for layperson transport systems; optimal mode of transport by setting; evaluation strategies to demonstrate impact of reducing prehospital transport times on patient outcomes and mortality	Scene: Bystander Response & Dispatch
	LFR Trainings	Utilize EORE community liaisons, de-mining medics and paramedics to lead ToT model LFR trainings for communities affected by explosive violence, including dissemination of the WHO CFAR training	Evaluation strategies demonstrating impact of LFR trainings on patient outcomes at receiving facilities; cost efficacy of LFR trainings; sustainability of ToT model	
Prehospital	Prehospital Notification Systems	Improved coordination between mine action medical teams, WHO, UN OCHA Health Cluster Tool/ HeRAMS for trauma capacity actor mapping with harmonized pathways for notification and casualty routing	Protocols for coordination between mine action medics and prehospital personnel/ WHO EMTs; clinical guidance on tourniquet application in prolonged prehospital transport times; translation of clinical advances in high-resource settings such as hemostatic agents and the use of whole blood	Transport: Patient Transport & Transport Care
	Prehospital Trauma Trainings	De-mining team medics and paramedics can contribute to trauma care trainings for local prehospital personnel, particularly through enhanced coordination with the WHO EMT Initiative		
Facility-Based	Trauma Team Organization/Activation	Bidirectional trainings can be conducted at health facilities receiving civilian casualties of EW wherein de-mining team medics/paramedics receive refresher trainings and participate in clinical capacity-building; HMA stakeholders can contribute to advocacy for targeted subspecialty trainings for trauma and surgical providers around injury patterns affecting EW casualties (eg, thermal injuries, traumatic amputation)	Clinical gaps exist with respect to blast-injury-specific trainings such as burns, mangled extremity/ compartment syndrome, and vulnerable subpopulations such as pediatrics	Facility: Emergency, Critical, and Operative Care
	Facility-Based Trauma Trainings			

Note: WHO Emergency Care System Framework: https://www.who.int/publications/i/item/who-emergency-care-system-framework.

Abbreviations: CFAR, Community First Aid Responder; EMT, Emergency Medical Teams; EORE, Explosive Ordnance Risk Education; EW, Explosive Weapons; HeRAMS, Health Resources and Services Availability Monitoring System; LFR, Layperson First Responder; ToT, Training of Trainers; WHO, World Health Organization.

### Heterogeneity in Intervention Format, Oversight, and Outcomes Reporting

Even in the absence of well-resourced formal emergency care systems, a coordinated approach engaging local health actors in the implementation of evidence-based trauma care practices can save lives. This was demonstrated by the Tromsø Mine Victim Center’s “Village University” where trauma care training for LFRs and health care workers in areas of Iraq and Cambodia contaminated with mines reduced mortality from 40% to 15% over a five-year period.^34,76^ Yet, LFRs and prehospital TCTCs are not equivalent and vary widely with respect to course format, duration, resource intensiveness, and monitoring and evaluation strategies. For example, in contrast with the intensive format and five-year prospective follow-up of the “Village University” program, one LFR training program in Brazil was disseminated via television demonstration to reach a wide audience of commercial viewers.^56^ Such variation is under-studied but can be assumed to yield significant implications for intervention efficacy and outcomes. Further, engagement of local communities in intervention design varied by report. Community participation was a core tenet of the “Village University” program and is a recognized pillar of effective mine action.^77^ Context-appropriate adaptations included initial needs assessment, use of translators in all local languages, measures to account for varying levels of participant education and literacy, and use of inexpensive and locally available materials such as cardboard, strings, paper towels, and goat carcasses.^78–81^ Adaptation of trauma care interventions in conflict requires even greater attention to contextual variability, mandating close engagement with local actors. Overall, the heterogeneity observed highlights an opportunity to create best practice standards including monitoring and evaluation strategies for TCTC design in conflict-affected and resource-constrained settings more broadly.^82,83^

### Evidence Gaps

This review identified numerous gaps and opportunities for further research and targeted quality improvement initiatives. Concerningly, these coincide with some of the most vulnerable patient populations demonstrated to have disproportionately high mortality in conflict.^84^ Only two reports each were described in the areas of pediatrics and burn care.^48–51^ Multi-dimensional injuries (eg, combined blunt, penetrating, and/or burn injuries) commonly result from EO/EWs. The vulnerability of children to the impact of EWs in conflict has been examined in detail elsewhere.^84,85^ Several existing initiatives may catalyze advances in pediatric trauma care in conflict, including the Global Society for Humanitarian Pediatrics and the Pediatric Blast Injury Partnership.^86,87^ A dedicated Pediatric Trauma Resuscitation Course is in development by the latter group and should be promulgated as a supplemental module to existing TCTCs. Burn care guidelines and training initiatives exist, led by organizations including International Society for Burn Injuries (League City, Texas USA), Interburns (Cardiff, United Kingdom), and WHO.^88–91^ These efforts should be scaled and integrated with existing TCTCs focused on EO/EW casualties as thermal injury is one sequela of blast injury with particularly severe associated morbidity and far-reaching consequences for resource utilization, rehabilitation, and functional outcomes.

### C-CCC: Selected Interventions and Potential Implementation Strategies through Coordination with HMA

The HMA stakeholders have significant potential to contribute to support emergency care systems strengthening in settings affected by EO/EWs given the medical resources of demining teams and well-established operational presence in many conflict and post-conflict settings. Within the HMA sector, improved engagement in trauma care capacity building represents a concrete opportunity for implementation of victim assistance strategies as outlined in three critical mine action treaties (the APMBC, Convention on Cluster Munitions, and Protocol V of the Convention on Certain Conventional Weapons), as well as the recently adopted IMAS 13.10 on Victim Assistance in Mine Action.^92–94^ The following interventions do not represent a complete casualty care pathway, but rather highlight a series of selected areas in which HMA capabilities could be leveraged to significantly enhance health sector initiatives to improve the outcomes of civilians injured by EO/EWs.

At the LFR phase, HMA stakeholders can engage in LFR trainings in the EO/EW-affected communities where they work. Given the lack of organized prehospital transport systems in many resource-constrained conflict settings, linking this initiative with the development of organized layperson transport mechanisms (eg, motorcycles, bicycles, and donkeys) holds potential to reduce care delays.^57,58,95,96^ The impact of transport times on mortality is well-described, and prolonged prehospital times are a significant barrier to improving survivability of EO/EW-related injury.^97–99^ Building on existing HMA infrastructure, potential implementation strategies for LFR trainings would include an enhanced EORE package with trained community liaison staff. Key existing resources include the “Village University” curriculum and the WHO Community First Aid Responder (CFAR) training. Alternative implementation strategies could involve demining staff within local communities or at a safe periphery from a worksite, as these individuals are all trained to a level of basic trauma care provision in accordance with IMAS 10.40.^100^ Consideration must be given to adequate guidance to protect trained LFRs from sustaining injury if responding to EO/EW casualties and supporting their mental health after administering aid. In conjunction with these efforts, given the exemplary precedent set by the HMA sector for standardized data collection practices in challenging operational environments, layperson data enumerators could be trained to further strengthen surveillance.^101–103^

At the prehospital phase, HMA stakeholders can support TCTCs with more advanced content for prehospital medical personnel (eg, multiple casualty triage, care of multi-dimensional injuries/complex blast wounding patterns such as burns, mangled extremities/compartment syndrome, tympanic membrane rupture/ocular injuries, blast lung, and TBI), as well as prehospital notification systems. Key existing resources for prehospital TCTCs focused on blast injury include ICRC’s Blast Trauma Care Course and the WHO-ICRC Basic Emergency Care module on Conflict-Related Injuries.^91,104^ Prehospital notification systems were described by only one report included in this review, but in high-resource settings, have been identified as one of the trauma system components most associated with decreased mortality.^105^ Though such systems may face implementation challenges in areas of limited telecommunications, they likely represent an under-explored opportunity to improve patient outcomes. In other contexts, village radios have been used for emergency preparedness in remote settings and could be applied in the context of EO/EW casualty evacuation.^106^

Opportunities for HMA engagement at the facility-based phase are fewer due to multiple factors, including the diversity of stakeholders at this level as well as the complexity of clinical care provided. Nonetheless, actors within the HMA sector can contribute to clinical capacity-building by supporting trauma team organization (ie, the establishment of structured trauma teams with designated roles that engage in resuscitation rehearsals) and activation protocols (ie, mechanisms whereby a trauma activation is triggered by prehospital notification of incoming casualties).^36,55,69^ Resource requirements necessary to organize trauma teams among health facility staff, to establish protocols for trauma team activation, use checklists, and conduct trauma resuscitation rehearsals are relatively minimal with potentially significant impact on patient outcomes.^107^ For example, the use of a standardized trauma intake form even in the absence of formal trauma teams was found to reduce trauma-related mortality from 17.7% to 12.1% at one hospital in Ghana.^108^ The HMA stakeholders can also help advocate for the importance of specialized facility-based TCTCs addressing key issues affecting EO/EW casualties (eg, amputation techniques and soft tissue reconstruction), which have demonstrated significant capacity to improve rates of limb salvage.^55,69^ Such initiatives must be contextualized within broader emergency care systems strengthening led by national and multi-lateral health stakeholders, and require close coordination with Ministries of Health and the WHO.^109^

Potential HMA implementation partners at the prehospital and facility-based phases include paramedics (designated intermediate care providers [ICPs]), with training delivered either for additional incentives during off-duty hours at a safe periphery from a worksite so long as they remained within the worksite’s designated fixed response time, or separately arranged to coincide with their own refresher trainings at a health facility. Providing training in such an arrangement is in accordance with IMAS 10.40 3.2.2, which stipulates that ICPs may “fulfill a dual role in low-risk clerical duties outside the active worksite.”^100^ In instances where ICPs were accredited through their affiliated mine action organization in a process separate from local health institutions, memoranda of understanding with local authorities would need to be instituted to formalize this role and address liability concerns. Increased coordination with local Ministries of Health for facility-based interventions would be mutually beneficial for HMA stakeholders. Such engagement would provide refresher training opportunities for HMA ICPs as well as ensure that an adequate level of care is provided should they need to evacuate their own casualties to these sites, such as the tragic attack on the HALO compound in Afghanistan.^110^

## Limitations

This review had several limitations. First, to capture a range of trauma care interventions in settings applicable to civilian EO/EW casualties, no studies were excluded based on study quality. While study quality was descriptively assessed using a QUADS scoring framework, these evaluations were not used as exclusion criteria. Second, since study quality was utilized as a criterion to select interventions as links in the CCCC, and since training initiatives tended to have higher QUADS scores, training initiatives were favored in this synthesis. Other interventions exist with potential opportunities for HMA engagement, particularly at the level of coordination, readiness, preparedness, and response in resource-constrained conflict and post-conflict settings. Finally, significant heterogeneity exists in the data presented by included reports, which precluded pooled analysis. Nonetheless, this review conducts a comprehensive synthesis of the evidence base for trauma care interventions that may inform resource-feasible solutions to improving the care of EO/EW casualties.

## Conclusion

As indiscriminate use of landmines, cluster munitions, IEDs, and other EWs disproportionately affects civilians in conflict settings globally with reverberating consequences for decades from explosive remnants of war, improving the quality of trauma care for casualties is essential. This review synthesized the evidence base on trauma care interventions in resource-constrained settings applicable to the care of EO/EW casualties to propose a strategy for improved care and coordination between HMA stakeholders and trauma care providers. By linking the global and country-level humanitarian Health and Protection Clusters, HMA stakeholders can potentiate efforts to build capacity among trauma care providers in conflict-affected settings, reducing preventable death and disability caused by EWs.

## Supporting information

Wild et al. supplementary material Wild et al. supplementary material

## Supplementary Materials

To view supplementary material for this article, please visit https://doi.org/10.1017/S1049023X24000669
