# Supplementary material for: Synthesizing the Evidence Base to Enhance Coordination between Humanitarian Mine Action and Emergency Care for Casualties of Explosive Ordnance and Explosive Weapons: A Scoping Review
Source: Prehosp Disaster Med. 2025 Jan 24;39(6):421–35. doi: 10.1017/S1049023X24000669 (PMC11821299; doi:10.1017/S1049023X24000669)
Supplement: Wild et al. supplementary material [file S1049023X24000669sup001.docx]

**Supplemental Information**

Supplement 1. Search strings by database

Supplement 2. Review protocol

Supplement 3. PRISMA-SCr checklist

Supplement 4. Summary table of reports included in analysis

**Supplement 1. Search strings by database**

**PubMed**

|  | **Concept: trauma centers** | **Concept: injuries** |
| --- | --- | --- |
| Subject Headings  (MeSH) | "Emergency Medical Services"[Mesh]  "Emergency Service, Hospital"[Mesh]  "trauma centers"[Mesh] | "Wounds and Injuries"[Mesh:noexp]  "Abdominal Injuries"[Mesh]  "Accidental Injuries"[Mesh]  "Amputation, Traumatic"[Mesh]  "Arm Injuries"[Mesh]  "Asphyxia"[Mesh]  "Back Injuries"[Mesh]  "Barotrauma"[Mesh]  "Birth Injuries"[Mesh]  "Burns"[Mesh]  "Cold Injury"[Mesh]  "Contrecoup Injury"[Mesh]  "Crush Injuries"[Mesh]  "Drowning"[Mesh]  "Electric Injuries"[Mesh]  "Esophageal Perforation"[Mesh]  "Foreign Bodies"[Mesh]  "Fractures, Bone"[Mesh]  "Fractures, Cartilage"[Mesh]  "Frostbite"[Mesh]  "Hand Injuries"[Mesh]  "Heat Stress Disorders"[Mesh]  "Hip Injuries"[Mesh]  "Joint Dislocations"[Mesh]  "Lacerations"[Mesh]  "Leg Injuries"[Mesh]  "Microtrauma, Physical"[Mesh]  "Multiple Trauma"[Mesh]  "Nasal Septal Perforation"[Mesh]  "Neck Injuries"[Mesh]  "Occupational Injuries"[Mesh]  "Radiation Injuries"[Mesh]  "Reinjuries"[Mesh]  "Retropneumoperitoneum"[Mesh]  "Rupture"[Mesh]  "Self Mutilation"[Mesh]  "Shock, Traumatic"[Mesh]  "Shoulder Injuries"[Mesh]  "Soft Tissue Injuries"[Mesh]  "Spinal Cord Injuries"[Mesh]  "Sprains and Strains"[Mesh]  "Surgical Wound"[Mesh]  "Tendon Injuries"[Mesh]  "Thoracic Injuries"[Mesh]  "Tooth Injuries"[Mesh]  "Trauma, Nervous System"[Mesh]  "Tympanic Membrane Perforation"[Mesh]  "Vascular System Injuries"[Mesh]  "War-Related Injuries"[Mesh]  "Wounds, Nonpenetrating"[Mesh]  "Wounds, Penetrating"[Mesh]  "injuries"[Subheading] |
| Free text terms  (searched in [text words](https://pubmed.ncbi.nlm.nih.gov/help/#tw) [tw]) | "Emergency Medical Service*"  "Emergency Service*"  "emergency center*"  "emergency centre*"  "emergency health care service*"  "emergency healthcare service*"  "trauma centre*"  "trauma center*"  "trauma system"  "trauma systems"  "trauma care"  "casualty care"  "acute care"  "acute medical care"  "emergency health care"  "emergency medical care"  Emergicent*  **(**(prehospital OR "pre-hospital") AND (intervention* OR procedure* OR treatment* OR "care" OR management OR aid OR program* OR training*)**)** | trauma  wound*  injur*  fractur*  lacerat*  rupture*  perforat*  avulsion*  contusion*  barotrauma*  burn*  concussion*  hemorrhage*  hematoma* |

|  | **Concept: explosive ordnance** | **Concept: injuries** | **Concept: Medical management** |
| --- | --- | --- | --- |
| Subject Headings  (MeSH) | "Explosive agents"[Mesh]  "Bombs"[Mesh]  "Blast Injuries"[Mesh]  "Explosions"[Mesh] | "Wounds and Injuries"[Mesh:noexp]  "Abdominal Injuries"[Mesh]  "Accidental Injuries"[Mesh]  "Amputation, Traumatic"[Mesh]  "Arm Injuries"[Mesh]  "Asphyxia"[Mesh]  "Back Injuries"[Mesh]  "Barotrauma"[Mesh]  "Birth Injuries"[Mesh]  "Burns"[Mesh]  "Cold Injury"[Mesh]  "Contrecoup Injury"[Mesh]  "Crush Injuries"[Mesh]  "Drowning"[Mesh]  "Electric Injuries"[Mesh]  "Esophageal Perforation"[Mesh]  "Foreign Bodies"[Mesh]  "Fractures, Bone"[Mesh]  "Fractures, Cartilage"[Mesh]  "Frostbite"[Mesh]  "Hand Injuries"[Mesh]  "Heat Stress Disorders"[Mesh]  "Hip Injuries"[Mesh]  "Joint Dislocations"[Mesh]  "Lacerations"[Mesh]  "Leg Injuries"[Mesh]  "Microtrauma, Physical"[Mesh]  "Multiple Trauma"[Mesh]  "Nasal Septal Perforation"[Mesh]  "Neck Injuries"[Mesh]  "Occupational Injuries"[Mesh]  "Radiation Injuries"[Mesh]  "Reinjuries"[Mesh]  "Retropneumoperitoneum"[Mesh]  "Rupture"[Mesh]  "Self Mutilation"[Mesh]  "Shock, Traumatic"[Mesh]  "Shoulder Injuries"[Mesh]  "Soft Tissue Injuries"[Mesh]  "Spinal Cord Injuries"[Mesh]  "Sprains and Strains"[Mesh]  "Surgical Wound"[Mesh]  "Tendon Injuries"[Mesh]  "Thoracic Injuries"[Mesh]  "Tooth Injuries"[Mesh]  "Trauma, Nervous System"[Mesh]  "Tympanic Membrane Perforation"[Mesh]  "Vascular System Injuries"[Mesh]  "War-Related Injuries"[Mesh]  "Wounds, Nonpenetrating"[Mesh]  "Wounds, Penetrating"[Mesh]  "injuries"[Subheading] |  |
| Free text terms  (searched in [text words](https://pubmed.ncbi.nlm.nih.gov/help/#tw) [tw]) | APM  blast  bomb*  detonation*  EED  ERW  explosion*  explosive*  IED  grenade*  landmine*  "mine"  "mines"  missile*  mortar*  ordnance  rocket*  UXB  UXO | trauma  wound*  injur*  fractur*  lacerat*  rupture*  perforat*  avulsion*  contusion*  barotrauma*  burn*  concussion*  hemorrhage*  hematoma* | intervention*  procedure*  treatment*  "care"  management  aid  program*  training* |

**((**("Emergency Medical Services"[Mesh] OR "emergency service, hospital"[Mesh] OR "trauma centers"[Mesh] OR "Emergency Medical Service*"[tw] OR "Emergency Service*"[tw] OR "emergency center*"[tw] OR "emergency centre*"[tw] OR "emergency health care service*"[tw] OR "emergency healthcare service*"[tw] OR "trauma centre*"[tw] OR "trauma center*"[tw] OR "trauma system"[tw] OR "trauma systems"[tw] OR "trauma care"[tw] OR "casualty care"[tw] OR "acute care"[tw] OR "acute medical care"[tw] OR "emergency health care"[tw] OR "emergency medical care"[tw] OR Emergicent*[tw] OR ((prehospital[tw] OR "pre-hospital"[tw]) AND (intervention*[tw] OR procedure*[tw] OR treatment*[tw] OR "care"[tw] OR management[tw] OR aid[tw] OR program*[tw] OR training*[tw]))) **AND** ("Wounds and Injuries"[Mesh:noexp] OR "Abdominal Injuries"[Mesh] OR "Accidental Injuries"[Mesh] OR "Amputation, Traumatic"[Mesh] OR "Arm Injuries"[Mesh] OR "Asphyxia"[Mesh] OR "Back Injuries"[Mesh] OR "Barotrauma"[Mesh] OR "Birth Injuries"[Mesh] OR "Burns"[Mesh] OR "Cold Injury"[Mesh] OR "Contrecoup Injury"[Mesh] OR "Crush Injuries"[Mesh] OR "Drowning"[Mesh] OR "Electric Injuries"[Mesh] OR "Esophageal Perforation"[Mesh] OR "Foreign Bodies"[Mesh] OR "Fractures, Bone"[Mesh] OR "Fractures, Cartilage"[Mesh] OR "Frostbite"[Mesh] OR "Hand Injuries"[Mesh] OR "Heat Stress Disorders"[Mesh] OR "Hip Injuries"[Mesh] OR "Joint Dislocations"[Mesh] OR "Lacerations"[Mesh] OR "Leg Injuries"[Mesh] OR "Microtrauma, Physical"[Mesh] OR "Multiple Trauma"[Mesh] OR "Nasal Septal Perforation"[Mesh] OR "Neck Injuries"[Mesh] OR "Occupational Injuries"[Mesh] OR "Radiation Injuries"[Mesh] OR "Reinjuries"[Mesh] OR "Retropneumoperitoneum"[Mesh] OR "Rupture"[Mesh] OR "Self Mutilation"[Mesh] OR "Shock, Traumatic"[Mesh] OR "Shoulder Injuries"[Mesh] OR "Soft Tissue Injuries"[Mesh] OR "Spinal Cord Injuries"[Mesh] OR "Sprains and Strains"[Mesh] OR "Surgical Wound"[Mesh] OR "Tendon Injuries"[Mesh] OR "Thoracic Injuries"[Mesh] OR "Tooth Injuries"[Mesh] OR "Trauma, Nervous System"[Mesh] OR "Tympanic Membrane Perforation"[Mesh] OR "Vascular System Injuries"[Mesh] OR "War-Related Injuries"[Mesh] OR "Wounds, Nonpenetrating"[Mesh] OR "Wounds, Penetrating"[Mesh] OR "injuries"[Subheading] OR trauma[tw] OR wound*[tw] OR injur*[tw] OR fractur*[tw] OR lacerat*[tw] OR rupture*[tw] OR perforat*[tw] OR avulsion*[tw] OR contusion*[tw] OR barotrauma*[tw] OR burn*[tw] OR concussion*[tw] OR hemorrhage*[tw] OR hematoma*[tw])**)**

**OR**

**(**("Explosive agents"[Mesh] OR "Bombs"[Mesh] OR "Blast Injuries"[Mesh] OR "Explosions"[Mesh] OR APM[tw] OR blast[tw] OR bomb*[tw] OR detonation*[tw] OR EED[tw] OR ERW[tw] OR explosion*[tw] OR explosive*[tw] OR IED[tw] OR grenade*[tw] OR landmine*[tw] OR "mine"[tw] OR "mines"[tw] OR missile*[tw] OR mortar*[tw] OR ordnance[tw] OR rocket*[tw] OR UXB[tw] OR UXO[tw]) **AND** ("Wounds and Injuries"[Mesh:noexp] OR "Abdominal Injuries"[Mesh] OR "Accidental Injuries"[Mesh] OR "Amputation, Traumatic"[Mesh] OR "Arm Injuries"[Mesh] OR "Asphyxia"[Mesh] OR "Back Injuries"[Mesh] OR "Barotrauma"[Mesh] OR "Birth Injuries"[Mesh] OR "Burns"[Mesh] OR "Cold Injury"[Mesh] OR "Contrecoup Injury"[Mesh] OR "Crush Injuries"[Mesh] OR "Drowning"[Mesh] OR "Electric Injuries"[Mesh] OR "Esophageal Perforation"[Mesh] OR "Foreign Bodies"[Mesh] OR "Fractures, Bone"[Mesh] OR "Fractures, Cartilage"[Mesh] OR "Frostbite"[Mesh] OR "Hand Injuries"[Mesh] OR "Heat Stress Disorders"[Mesh] OR "Hip Injuries"[Mesh] OR "Joint Dislocations"[Mesh] OR "Lacerations"[Mesh] OR "Leg Injuries"[Mesh] OR "Microtrauma, Physical"[Mesh] OR "Multiple Trauma"[Mesh] OR "Nasal Septal Perforation"[Mesh] OR "Neck Injuries"[Mesh] OR "Occupational Injuries"[Mesh] OR "Radiation Injuries"[Mesh] OR "Reinjuries"[Mesh] OR "Retropneumoperitoneum"[Mesh] OR "Rupture"[Mesh] OR "Self Mutilation"[Mesh] OR "Shock, Traumatic"[Mesh] OR "Shoulder Injuries"[Mesh] OR "Soft Tissue Injuries"[Mesh] OR "Spinal Cord Injuries"[Mesh] OR "Sprains and Strains"[Mesh] OR "Surgical Wound"[Mesh] OR "Tendon Injuries"[Mesh] OR "Thoracic Injuries"[Mesh] OR "Tooth Injuries"[Mesh] OR "Trauma, Nervous System"[Mesh] OR "Tympanic Membrane Perforation"[Mesh] OR "Vascular System Injuries"[Mesh] OR "War-Related Injuries"[Mesh] OR "Wounds, Nonpenetrating"[Mesh] OR "Wounds, Penetrating"[Mesh] OR "Hemorrhage"[Mesh] OR "injuries"[Subheading] OR trauma[tw] OR wound*[tw] OR injur*[tw] OR fractur*[tw] OR lacerat*[tw] OR rupture*[tw] OR perforat*[tw] OR avulsion*[tw] OR contusion*[tw] OR barotrauma*[tw] OR burn*[tw] OR concussion*[tw] OR hemorrhage*[tw] OR hematoma*[tw]) **AND** (intervention*[tw] OR procedure*[tw] OR treatment*[tw] OR "care"[tw] OR management[tw] OR aid[tw])**))**

**AND**

**((Afghanistan[Mesh] OR Albania[Mesh] OR Algeria[Mesh] OR American Samoa[Mesh] OR Angola[Mesh] OR Antigua and Barbuda[Mesh] OR Argentina[Mesh] OR Armenia[Mesh] OR Aruba[Mesh] OR Azerbaijan[Mesh] OR Bahrain[Mesh] OR Bangladesh[Mesh] OR Barbados[Mesh] OR Republic of Belarus[Mesh] OR Belize[Mesh] OR Benin[Mesh] OR Bhutan[Mesh] OR Bolivia[Mesh] OR Bosnia and Herzegovina[Mesh] OR Botswana[Mesh] OR Brazil[Mesh] OR Bulgaria[Mesh] OR Burkina Faso[Mesh] OR Burundi[Mesh] OR Cabo Verde[Mesh] OR Cambodia[Mesh] OR Cameroon[Mesh] OR Central African Republic[Mesh] OR Chad[Mesh] OR Chile[Mesh] OR Colombia[Mesh] OR Comoros[Mesh] OR Democratic Republic of The Congo[Mesh] OR Congo[Mesh] OR Costa Rica[Mesh] OR Cote D’ivoire[Mesh] OR Croatia[Mesh] OR Cuba[Mesh] OR Cyprus[Mesh] OR Czech Republic[Mesh] OR Djibouti[Mesh] OR Dominica[Mesh] OR Dominican Republic[Mesh] OR Ecuador[Mesh] OR Egypt[Mesh] OR El Salvador[Mesh] OR Equatorial Guinea[Mesh] OR Eritrea[Mesh] OR Estonia[Mesh] OR Swaziland[Mesh] OR Ethiopia[Mesh] OR Fiji[Mesh] OR Gabon[Mesh] OR Gambia[Mesh] OR "Georgia (Republic)"[Mesh] OR Ghana[Mesh] OR Gibraltar[Mesh] OR Greece[Mesh] OR Grenada[Mesh] OR Guam[Mesh] OR Guatemala[Mesh] OR Guinea[Mesh] OR Guinea Bissau[Mesh] OR Guyana[Mesh] OR Haiti[Mesh] OR Honduras[Mesh] OR India[Mesh] OR Indonesia[Mesh] OR Iran[Mesh] OR Iraq[Mesh] OR Jamaica[Mesh] OR Jordan[Mesh] OR Kazakhstan[Mesh] OR Kenya[Mesh] OR Democratic People’s Republic of Korea[Mesh] OR Republic of Korea[Mesh] OR Kosovo[Mesh] OR Kyrgyzstan[Mesh] OR Laos[Mesh] OR Latvia[Mesh] OR Lebanon[Mesh] OR Lesotho[Mesh] OR Liberia[Mesh] OR Libya[Mesh] OR Lithuania[Mesh] OR Macau[Mesh] OR Republic of North Macedonia[Mesh] OR Madagascar[Mesh] OR Malawi[Mesh] OR Malaysia[Mesh] OR Indian Ocean Islands[Mesh] OR Mali[Mesh] OR Malta[Mesh] OR Micronesia[Mesh] OR Palau[Mesh] OR Mauritania[Mesh] OR Mauritius[Mesh] OR Mexico[Mesh] OR Moldova[Mesh] OR Mongolia[Mesh] OR Montenegro[Mesh] OR Morocco[Mesh] OR Mozambique[Mesh] OR Myanmar[Mesh] OR Namibia[Mesh] OR Nepal[Mesh] OR Netherlands Antilles[Mesh] OR Nicaragua[Mesh] OR Niger[Mesh] OR Nigeria[Mesh] OR Oman[Mesh] OR Pakistan[Mesh] OR Panama[Mesh] OR Papua New Guinea[Mesh] OR Paraguay[Mesh] OR Peru[Mesh] OR Philippines[Mesh] OR Portugal[Mesh] OR Puerto Rico[Mesh] OR Romania[Mesh] OR Rwanda[Mesh] OR Samoa[Mesh] OR Sao Tome and Principe[Mesh] OR Senegal[Mesh] OR Seychelles[Mesh] OR Sierra Leone[Mesh] OR Melanesia[Mesh] OR Somalia[Mesh] OR South Africa[Mesh] OR South Sudan[Mesh] OR Sri Lanka[Mesh] OR Saint Kitts and Nevis[Mesh] OR Saint Lucia[Mesh] OR Saint Vincent and The Grenadines[Mesh] OR Sudan[Mesh] OR Suriname[Mesh] OR Syria[Mesh] OR Tajikistan[Mesh] OR Tanzania[Mesh] OR Thailand[Mesh] OR Timor Leste[Mesh] OR Togo[Mesh] OR Tonga[Mesh] OR Trinidad and Tobago[Mesh] OR Tunisia[Mesh] OR Turkmenistan[Mesh] OR Uganda[Mesh] OR Ukraine[Mesh] OR Uruguay[Mesh] OR Uzbekistan[Mesh] OR Vanuatu[Mesh] OR Venezuela[Mesh] OR Vietnam[Mesh] OR Yemen[Mesh] OR Yugoslavia[Mesh] OR Zambia[Mesh] OR Zimbabwe[Mesh] OR Africa South of The Sahara[Mesh] OR Africa, Central[Mesh] OR Africa, Northern[Mesh] OR Africa, Southern[Mesh] OR Africa, Eastern[Mesh] OR Africa, Western[Mesh] OR West Indies[Mesh] OR Indian Ocean Islands[Mesh] OR Caribbean Region[Mesh] OR Central America[Mesh] OR Latin America[Mesh] OR South America[Mesh] OR Asia, Central[Mesh] OR Asia, Northern[Mesh:noexp] OR Asia, Southeastern[Mesh] OR** Asia, Western[Mesh:noexp] OR Middle East[Mesh:noexp] OR Europe, Eastern[Mesh:noexp**])**

OR

(Afghanistan[tw] OR Albania[tw] OR Algeria[tw] OR American Samoa[tw] OR Angola[tw] OR Antigua[tw] OR Barbuda[tw] OR Argentina[tw] OR "Argentine Republic"[tw] OR Armenia[tw] OR Armenian[tw] OR Aruba[tw] OR Azerbaijan[tw] OR Bahrain[tw] OR Bangladesh[tw] OR Barbados[tw] OR Belarus[tw] OR Byelarus[tw] OR Belorussia[tw] OR Byelorussian[tw] OR Belize[tw] OR British Honduras[tw] OR Benin[tw] OR Dahomey[tw] OR Bhutan[tw] OR Bolivia[tw] OR Bosnia[tw] OR Hercegovina[tw] OR Herzegovina[tw] OR Botswana[tw] OR Bechuanaland[tw] OR Brazil[tw] OR Brasil[tw] OR Bulgaria[tw] OR Burkina Faso[tw] OR Burkina Fasso[tw] OR Upper Volta[tw] OR Burundi[tw] OR Urundi[tw] OR Cabo Verde[tw] OR Cape Verde[tw] OR Cambodia[tw] OR Kampuchea[tw] OR Khmer Republic[tw] OR Cameroon[tw] OR Cameron[tw] OR Cameroun[tw] OR Central African Republic[tw] OR Ubangi Shari[tw] OR Chad[tw] OR Chile[tw] OR Colombia[tw] OR Comoros[tw] OR Comores[tw] OR Comoro Islands[tw] OR Mayotte[tw] OR Congo[tw] OR Zaire[tw] OR Costa Rica[tw] OR Cote D’ivoire[tw] OR Cote D’ Ivoire[tw] OR Cote Divoire[tw] OR Cote D Ivoire[tw] OR Ivory Coast[tw] OR Croatia[tw] OR Cuba[tw] OR Cyprus[tw] OR Czech Republic[tw] OR Czechoslovakia[tw] OR Djibouti[tw] OR French Somaliland[tw] OR Dominica[tw] OR Dominican Republic[tw] OR Ecuador[tw] OR Egypt[tw] OR United Arab Republic[tw] OR El Salvador[tw] OR Equatorial Guinea[tw] OR Spanish Guinea[tw] OR Eritrea[tw] OR Estonia[tw] OR Eswatini[tw] OR Swaziland[tw] OR Ethiopia[tw] OR Fiji[tw] OR Gabon[tw] OR Gabonese Republic[tw] OR Gambia[tw] OR Georgia[tw] OR Georgian[tw] OR Ghana[tw] OR Gold Coast[tw] OR Gibraltar[tw] OR Greece[tw] OR Grenada[tw] OR Guam[tw] OR Guatemala[tw] OR Guinea[tw] OR Guyana[tw] OR Guiana[tw] OR Haiti[tw] OR Hispaniola[tw] OR Honduras[tw] OR India[tw] OR Indonesia[tw] OR Timor[tw] OR Iran[tw] OR Iraq[tw] OR Isle Of Man[tw] OR Jamaica[tw] OR Jordan[tw] OR Kazakhstan[tw] OR Kazakh[tw] OR Kenya[tw] OR Korea[tw] OR Kosovo[tw] OR Kyrgyzstan[tw] OR Kirghizia[tw] OR Kirgizstan[tw] OR Kyrgyz Republic[tw] OR Kirghiz[tw] OR Laos[tw] OR Lao Pdr[tw] OR Lao People's Democratic Republic[tw] OR Latvia[tw] OR Lebanon[tw] OR Lesotho[tw] OR Basutoland[tw] OR Liberia[tw] OR Libya[tw] OR Libyan Arab Jamahiriya[tw] OR Lithuania[tw] OR Macau[tw] OR Macao[tw] OR Macedonia[tw] OR Madagascar[tw] OR Malagasy Republic[tw] OR Malawi[tw] OR Nyasaland[tw] OR Malaysia[tw] OR Maldives[tw] OR Indian Ocean[tw] OR Mali[tw] OR Malta[tw] OR Micronesia[tw] OR Kiribati[tw] OR Marshall Islands[tw] OR Nauru[tw] OR Northern Mariana Islands[tw] OR Palau[tw] OR Tuvalu[tw] OR Mauritania[tw] OR Mauritius[tw] OR Mexico[tw] OR Moldova[tw] OR Moldovian[tw] OR Mongolia[tw] OR Montenegro[tw] OR Morocco[tw] OR Ifni[tw] OR Mozambique[tw] OR Portuguese East Africa[tw] OR Myanmar[tw] OR Burma[tw] OR Namibia[tw] OR Nepal[tw] OR Netherlands Antilles[tw] OR Nicaragua[tw] OR Niger[tw] OR Nigeria[tw] OR Oman[tw] OR Muscat[tw] OR Pakistan[tw] OR Panama[tw] OR Papua New Guinea[tw] OR Paraguay[tw] OR Peru[tw] OR Philippines[tw] OR Philipines[tw] OR Phillipines[tw] OR Phillippines[tw] OR Portugal[tw] OR Portuguese Republic[tw] OR Puerto Rico[tw] OR Romania[tw] OR Rwanda[tw] OR Ruanda[tw] OR Samoa[tw] OR Pacific Islands[tw] OR Polynesia[tw] OR Samoan Islands[tw] OR Sao Tome And Principe[tw] OR Senegal[tw] OR Seychelles[tw] OR Sierra Leone[tw] OR Melanesia[tw] OR Solomon Island[tw] OR Solomon Islands[tw] OR Norfolk Island[tw] OR Somalia[tw] OR South Africa[tw] OR South Sudan[tw] OR Sri Lanka[tw] OR Ceylon[tw] OR Saint Kitts And Nevis[tw] OR St Kitts And Nevis[tw] OR Saint Lucia[tw] OR St Lucia[tw] OR Saint Vincent[tw] OR St Vincent[tw] OR Grenadines[tw] OR Sudan[tw] OR Suriname[tw] OR Surinam[tw] OR Syria[tw] OR Syrian Arab Republic[tw] OR Tajikistan[tw] OR Tadjikistan[tw] OR Tadzhikistan[tw] OR Tadzhik[tw] OR Tanzania[tw] OR Tanganyika[tw] OR Thailand[tw] OR Siam[tw] OR Timor Leste[tw] OR East Timor[tw] OR Togo[tw] OR Togolese Republic[tw] OR Tonga[tw] OR Trinidad[tw] OR Tobago[tw] OR Tunisia[tw] OR Turkmenistan[tw] OR Turkmen[tw] OR Uganda[tw] OR Ukraine[tw] OR Uruguay[tw] OR Uzbekistan[tw] OR Uzbek[tw] OR Vanuatu[tw] OR New Hebrides[tw] OR Venezuela[tw] OR Vietnam[tw] OR Viet Nam[tw] OR West Bank[tw] OR Gaza[tw] OR Palestine[tw] OR Yemen[tw] OR Yugoslavia[tw] OR Zambia[tw] OR Zimbabwe[tw] OR Northern Rhodesia[tw])

OR

("global south"[tw] OR Africa*[tw] OR Magreb[tw] OR Maghreb[tw] OR Sahara[tw] OR Sahel[tw] OR "West Indies"[tw] OR "Indian Ocean islands"[tw] OR Caribbean[tw] OR "Central America*"[tw] OR "Latin America*"[tw] OR "South America*"[tw] OR "Central Asia*"[tw] OR "north Asia*"[tw] OR "northern Asia*"[tw] OR "southeastern Asia*"[tw] OR "south eastern Asia*"[tw] OR "southeast Asia*"[tw] OR "south east Asia*"[tw] OR "western Asia*"[tw] OR "Middle East*"[tw] OR "east Europe*"[tw] OR "eastern Europe*"[tw])

OR

(**Developing Countries[Mesh] OR** developing country[tw] OR developing countries[tw] OR developing nation[tw] OR developing nations[tw] OR developing population[tw] OR developing populations[tw] OR developing world[tw] OR less developed country[tw] OR less developed countries[tw] OR less developed nation[tw] OR less developed nations[tw] OR less developed world[tw] OR lesser developed countries[tw] OR lesser developed nations[tw] OR under developed country[tw] OR under developed countries[tw] OR under developed nations[tw] OR under developed world[tw] OR underdeveloped country[tw] OR underdeveloped countries[tw] OR underdeveloped nation[tw] OR underdeveloped nations[tw] OR underdeveloped population[tw] OR underdeveloped populations[tw] OR underdeveloped world[tw] OR middle income country[tw] OR middle income countries[tw] OR middle income nation[tw] OR middle income nations[tw] OR middle income population[tw] OR middle income populations[tw] OR low income country[tw] OR low income countries[tw] OR low income nation[tw] OR low income nations[tw] OR low income population[tw] OR low income populations[tw] OR lower income country[tw] OR lower income countries[tw] OR lower income nations[tw] OR lower income population[tw] OR lower income populations[tw] OR underserved countries[tw] OR underserved nations[tw] OR underserved population[tw] OR underserved populations[tw] OR under served population[tw] OR under served populations[tw] OR deprived countries[tw] OR deprived population[tw] OR deprived populations[tw] OR poor country[tw] OR poor countries[tw] OR poor nation[tw] OR poor nations[tw] OR poor population[tw] OR poor populations[tw] OR poor world[tw] OR poorer countries[tw] OR poorer nations[tw] OR poorer population[tw] OR poorer populations[tw] OR developing economy[tw] OR developing economies[tw] OR less developed economy[tw] OR less developed economies[tw] OR underdeveloped economies[tw] OR middle income economy[tw] OR middle income economies[tw] OR low income economy[tw] OR low income economies[tw] OR lower income economies[tw] OR low gdp[tw] OR low gnp[tw] OR low gross domestic[tw] OR low gross national[tw] OR lower gdp[tw] OR lower gross domestic[tw] OR lmic[tw] OR lmics[tw] OR third world[tw] OR lami country[tw] OR lami countries[tw] OR transitional country[tw] OR transitional countries[tw] OR emerging economies[tw] OR emerging nation[tw] OR emerging nations[tw] OR "low resource" [tw] OR austere[tw])

OR

(Afghan[tw] OR Afghans[tw] OR Afghani[tw] OR Albanian[tw] OR Albanians[tw] OR Algerian[tw] OR Algerians[tw] OR American Samoan[tw] OR American Samoans[tw] OR Angolan[tw] OR Angolans[tw] OR Antiguan[tw] OR Antiguans[tw] OR Barbudan[tw] OR Berbudans[tw] OR Argentine[tw] OR Argentines[tw] OR Argentinian[tw] OR Argentinians[tw] OR Argentinean[tw] OR Argentineans[tw] OR Armenian[tw] OR Armenians[tw] OR Aruban[tw] OR Arubans[tw] OR Azerbaijani[tw] OR Azerbaijanis[tw] OR Bahraini[tw] OR Bahrainis[tw] OR Bangladeshi[tw] OR Bangladeshis[tw] OR Bangalees[tw] OR Bajan[tw] OR Bajans[tw] OR Barbadian[tw] OR Barbadians[tw] OR Belarusian[tw] OR Belarusians[tw] OR Byelorussian[tw] OR Byelorussians[tw] OR Belizean[tw] OR Belizeans[tw] OR Beninese[tw] OR Benineses[tw] OR Bhutanese[tw] OR Bolivian[tw] OR Bolivians[tw] OR Bosnian[tw] OR Bosnians[tw] OR Botswana[tw] OR Batswana[tw] OR Brazilian[tw] OR Brazilians[tw] OR Brasilian[tw] OR Brasilians[tw] OR Bulgarian[tw] OR Bulgarians[tw] OR Burkinabe[tw] OR Burkinese[tw] OR Burundian[tw] OR Burundians[tw] OR Cape Verdean[tw] OR Cape Verdeans[tw] OR Cabo Verdean[tw] OR Cabo Verdeans[tw] OR Cambodian[tw] OR Cambodians[tw] OR Khmer[tw] OR Cameroonian[tw] OR Cameroonians[tw] OR Central African[tw] OR Central Africans[tw] OR Chadian[tw] OR Chadians[tw] OR Chilean[tw] OR Chileans[tw] OR Colombian[tw] OR Colombians[tw] OR Comorian[tw] OR Comorians[tw] OR Congolese[tw] OR Costa Rican[tw] OR Costa Ricans[tw] OR Ivorian[tw] OR Ivorians[tw] OR Croatian[tw] OR Croatians[tw] OR Cuban[tw] OR Cubans[tw] OR Cypriot[tw] OR Cypriots[tw] OR Czech[tw] OR Czechs[tw] OR Djiboutian[tw] OR Djiboutians[tw] OR Dominican[tw] OR Dominicans[tw] OR Ecuadorian[tw] OR Ecuadorians[tw] OR Egyptian[tw] OR Egyptians[tw] OR Salvadoran[tw] OR Salvadorans[tw] OR Equatorial Guinean[tw] OR Equatorial Guineans[tw] OR Equatoguinean[tw] OR Equatoguineans[tw] OR Eritrean[tw] OR Eritreans[tw] OR Estonian[tw] OR Estonians[tw] OR Swazi[tw] OR Swazis[tw] OR Swati[tw] OR Swatis[tw] OR Ethiopian[tw] OR Ethiopians[tw] OR Fijian[tw] OR Fijians[tw] OR Gabonese[tw] OR Gabonaise[tw] OR Gambian[tw] OR Gambians[tw] OR Georgian[tw] OR Georgians[tw] OR Ghanaian[tw] OR Ghanaians[tw] OR Gibraltarian[tw] OR Gibraltarians[tw] OR Greek[tw] OR Greeks[tw] OR Grenadian[tw] OR Grenadians[tw] OR Guamanian[tw] OR Guamanians[tw] OR Guatemalan[tw] OR Guatemalans[tw] OR Guinean[tw] OR Guineans[tw] OR Bissau Guinean[tw] OR Bissau Guineans[tw] OR Guyanese[tw] OR Haitian[tw] OR Haitians[tw] OR Honduran[tw] OR Hondurans[tw] OR Indian[tw] OR Indians[tw] OR Indonesian[tw] OR Indonesians[tw] OR Iranian[tw] OR Iranians[tw] OR Iraqian[tw] OR Iraqians[tw] OR Iraqi[tw] OR Iraqis[tw] OR Manx[tw] OR Jamaican[tw] OR Jamaicans[tw] OR Jordanian[tw] OR Jordanians[tw] OR Kazakhstani[tw] OR Kazakhstanis[tw] OR Kenyan[tw] OR Kenyans[tw] OR Kirabati[tw] OR Kirabatian[tw] OR Kirabatians[tw] OR Korean[tw] OR Koreans[tw] OR Kosovar[tw] OR Kosovars[tw] OR Kosovan[tw] OR Kosovans[tw] OR Kyrgyzstani[tw] OR Kyrgyzstanis[tw] OR Kyrgyz[tw] OR Lao[tw] OR Laotian[tw] OR Laotians[tw] OR Latvian[tw] OR Latvians[tw] OR Lebanese[tw] OR Lesothan[tw] OR Lesothans[tw] OR Lesothonian[tw] OR Lesothonians[tw] OR Mosotho[tw] OR Basotho[tw] OR Liberian[tw] OR Liberians[tw] OR Libyan[tw] OR Libyans[tw] OR Lithuanian[tw] OR Lithuanians[tw] OR Macanese[tw] OR Macedonian[tw] OR Macedonians[tw] OR Malagasy[tw] OR Madagascan[tw] OR Madagascans[tw] OR Malawian[tw] OR Malawians[tw] OR Malay[tw] OR Malaya[tw] OR Malaysian[tw] OR Malaysians[tw] OR Maldivian[tw] OR Maldivians[tw] OR Malian[tw] OR Malians[tw] OR Maltese[tw] OR Marshallese[tw] OR Marshalleses[tw] OR Mauritanian[tw] OR Mauritanians[tw] OR Mauritian[tw] OR Mauritians[tw] OR Mexican[tw] OR Mexicans[tw] OR Micronesian[tw] OR Micronesians[tw] OR Moldovan[tw] OR Moldovans[tw] OR Mongolian[tw] OR Mongolians[tw] OR Mongol[tw] OR Montenegrin[tw] OR Montenegrins[tw] OR Moroccan[tw] OR Moroccans[tw] OR Mozambican[tw] OR Mozambicans[tw] OR Burmese[tw] OR Myanma[tw] OR Namibian[tw] OR Namibians[tw] OR Nauruan[tw] OR Nauruans[tw] OR Nepali[tw] OR Nepalese[tw] OR Netherlands Antillean[tw] OR Netherlands Antilleans[tw] OR Nicaraguan[tw] OR Nicaraguans[tw] OR Nigerien[tw] OR Nigeriens[tw] OR Nigerian[tw] OR Nigerians[tw] OR Northern Mariana Islander[tw] OR Northern Mariana Islanders[tw] OR Mariana[tw] OR Marianas[tw] OR Omani[tw] OR Omanis[tw] OR Pakistani[tw] OR Pakistanis[tw] OR Palauan[tw] OR Palauans[tw] OR Palestinian[tw] OR Palestinians[tw] OR Panamanian[tw] OR Panamanians[tw] OR Papua New Guinean[tw] OR Papua New Guineans[tw] OR Paraguayan[tw] OR Paraguayans[tw] OR Peruvian[tw] OR Peruvians[tw] OR Philippine[tw] OR Philippines[tw] OR Philipine[tw] OR Philipines[tw] OR Phillipine[tw] OR Phillipines[tw] OR Phillippine[tw] OR Phillippines[tw] OR Filipino[tw] OR Filipinos[tw] OR Filipina[tw] OR Filipinas[tw] OR Portuguese[tw] OR Puerto Rican[tw] OR Puerto Ricans[tw] OR Romanian[tw] OR Romanians[tw] OR Rwandan[tw] OR Rwandans[tw] OR Rwandese[tw] OR Ruandan[tw] OR Ruandans[tw] OR Ruandese[tw] OR Samoan[tw] OR Samoans[tw] OR Sao Tomean[tw] OR Sao Tomeans[tw] OR Santomean[tw] OR Santomeans[tw] OR Senegalese[tw] OR Montenegrin[tw] OR Montenegrins[tw] OR Seychellois[tw] OR Seychelloise[tw] OR Seychelloises[tw] OR Sierra Leonean[tw] OR Sierra Leoneans[tw] OR Solomon Islander[tw] OR Solomon Islanders[tw] OR Somali[tw] OR Somalis[tw] OR South African[tw] OR South Africans[tw] OR South Sudanese[tw] OR Sri Lankan[tw] OR Sri Lankans[tw] OR Ceylonese[tw] OR Kittitian[tw] OR Kittitians[tw] OR Nevisian[tw] OR Nevisians[tw] OR Saint Lucian[tw] OR Saint Lucians[tw] OR Vincentian[tw] OR Vincentians[tw] OR Sudanese[tw] OR Surinamese[tw] OR Surinameses[tw] OR Syrian[tw] OR Syrians[tw] OR Tajik[tw] OR Tajiks[tw] OR Tajikistani[tw] OR Tajikistanis[tw] OR Tanzanian[tw] OR Tanzanians[tw] OR Tanganyikan[tw] OR Tanganyikans[tw] OR Thai[tw] OR Timorese[tw] OR Timoreses[tw] OR Togolese[tw] OR Tongan[tw] OR Tongans[tw] OR Trinidadian[tw] OR Trinidadians[tw] OR Tobagonian[tw] OR Tobagonians[tw] OR Tunisian[tw] OR Tunisians[tw] OR Turkmen[tw] OR Turkmens[tw] OR Tuvaluan[tw] OR Tuvaluans[tw] OR Ugandan[tw] OR Ugandans[tw] OR Ukrainian[tw] OR Ukrainians[tw] OR Uruguayan[tw] OR Uruguayans[tw] OR Uzbek[tw] OR Uzbeks[tw] OR Vanuatu[tw] OR Vanuatuan[tw] OR Vanuatuans[tw] OR Venezuelan[tw] OR Venezuelans[tw] OR Vietnamese[tw] OR Yemeni[tw] OR Yemenis[tw] OR Yemenite[tw] OR Yemenites[tw] OR Yemenese[tw] OR Yugoslav[tw] OR Yugoslavs[tw] OR Yugoslavian[tw] OR Yugoslavians[tw] OR Zambian[tw] OR Zambians[tw] OR Zimbabwean[tw] OR Zimbabweans[tw])**)**

**NOT ("Plant Dis"[jour]) NOT (Animal[Mesh] NOT Human[Mesh])**

AND (2000/1/1:2021/12/31[pdat])

**Embase; Elsevier**

|  | **Concept: trauma centers** | **Concept: injuries** |
| --- | --- | --- |
| Subject Headings  (Emtree) | 'emergency health service'/exp  'emergency treatment'/exp  'emergency care'/exp | 'injury'/de  'abdominal injury'/exp  'abrasion'/exp  'accidental injury'/exp  'automutilation'/exp  'avulsion injury'/exp  'barotrauma'/exp  'battered child syndrome'/exp  'battle injury'/exp  'birth injury'/exp  'blood vessel injury'/exp  'blunt trauma'/exp  'breast injury'/exp  'burn'/exp  'chemical injury'/exp  'childhood injury'/exp  'concussion'/exp  'contrecoup injury'/exp  'contusion'/exp  'crush trauma'/exp  'drowning'/exp  'electric injury'/exp  'erosion'/exp  'experimental injury'/exp  'foreign body'/exp  'head and neck injury'/exp  'heart injury'/exp  'immune injury'/exp  'immune mediated injury'/exp  'injury scale'/exp  'injury severity'/exp  'limb injury'/exp  'membrane damage'/exp  'microtrauma'/exp  'multiple trauma'/exp  'musculoskeletal injury'/exp  'nervous system injury'/exp  'organ injury'/exp  'pelvis injury'/exp  'perforation'/exp  'prenatal injury'/exp  'psychotrauma'/exp  'radiation injury'/exp  'reperfusion injury'/exp  'respiratory tract injury'/exp  'rupture'/exp  'seatbelt injury'/exp  'skin injury'/exp  'soft tissue injury'/exp  'strangulation'/exp  'surgical injury'/exp  'thermal injury'/exp  'thorax injury'/exp  'tissue injury'/exp  'tooth injury'/exp  'traumatic amputation'/exp  'traumatic hematoma'/exp  'traumatic shock'/exp  'trench foot'/exp  'urogenital tract injury'/exp  'volutrauma'/exp  'wound'/exp |
| Free text terms  (searched in title, abstract, and keyword (:ti,ab,kw)) | 'Emergency Medical Service*'  'Emergency Service*'  'emergency center*'  'emergency centre*'  'emergency health care service*'  'emergency healthcare service*'  'trauma centre*'  'trauma center*'  'trauma system'  'trauma systems'  'trauma care'  'acute care'  'acute medical care'  'emergency health care'  'emergency medical care'  Emergicent*  **(**(prehospital OR 'pre-hospital') NEAR/3 (intervention* OR procedure* OR treatment* OR 'care' OR management OR aid OR program* OR training*)**)** | trauma  wound*  injur*  fractur*  lacerat*  rupture*  perforat*  avulsion*  contusion*  barotrauma*  burn*  concussion*  hemorrhage*  hematoma* |

|  | **Concept: explosive ordnance** | **Concept: injuries** | **Concept: Medical management** |
| --- | --- | --- | --- |
| Subject Headings  (Emtree) | 'explosive'/exp  'bomb'/exp  'weapon of mass destruction'/exp  'blast injury'/exp  'explosion'/exp | 'injury'/de  'abdominal injury'/exp  'abrasion'/exp  'accidental injury'/exp  'automutilation'/exp  'avulsion injury'/exp  'barotrauma'/exp  'battered child syndrome'/exp  'battle injury'/exp  'birth injury'/exp  'blood vessel injury'/exp  'blunt trauma'/exp  'breast injury'/exp  'burn'/exp  'chemical injury'/exp  'childhood injury'/exp  'concussion'/exp  'contrecoup injury'/exp  'contusion'/exp  'crush trauma'/exp  'drowning'/exp  'electric injury'/exp  'erosion'/exp  'experimental injury'/exp  'foreign body'/exp  'head and neck injury'/exp  'heart injury'/exp  'immune injury'/exp  'immune mediated injury'/exp  'injury scale'/exp  'injury severity'/exp  'limb injury'/exp  'membrane damage'/exp  'microtrauma'/exp  'multiple trauma'/exp  'musculoskeletal injury'/exp  'nervous system injury'/exp  'organ injury'/exp  'pelvis injury'/exp  'perforation'/exp  'prenatal injury'/exp  'psychotrauma'/exp  'radiation injury'/exp  'reperfusion injury'/exp  'respiratory tract injury'/exp  'rupture'/exp  'seatbelt injury'/exp  'skin injury'/exp  'soft tissue injury'/exp  'strangulation'/exp  'surgical injury'/exp  'thermal injury'/exp  'thorax injury'/exp  'tissue injury'/exp  'tooth injury'/exp  'traumatic amputation'/exp  'traumatic hematoma'/exp  'traumatic shock'/exp  'trench foot'/exp  'urogenital tract injury'/exp  'volutrauma'/exp  'wound'/exp |  |
| Free text terms  (searched in title, abstract, and keyword (:ti,ab,kw)) | APM  blast  bomb*  detonation*  EED  ERW  explosion*  explosive*  grenade*  IED  landmine*  'mine'  'mines'  missile*  mortar*  ordnance  rocket*  UXB  UXO | trauma  wound*  injur*  fractur*  lacerat*  rupture*  perforat*  avulsion*  contusion*  barotrauma*  burn*  concussion*  hemorrhage*  hematoma* | intervention*  procedure*  treatment*  "care"  management  aid  program*  training* |

**((**('emergency health service'/exp OR 'emergency treatment'/exp OR 'emergency care'/exp OR ('Emergency Medical Service*' OR 'Emergency Service*' OR 'emergency center*' OR 'emergency centre*' OR 'emergency health care service*' OR 'emergency healthcare service*' OR 'trauma centre*' OR 'trauma center*' OR 'trauma system' OR 'trauma systems' OR 'trauma care' OR 'acute care' OR 'acute medical care' OR 'emergency health care' OR 'emergency medical care'):ti,ab,kw OR ((prehospital OR 'pre-hospital') NEAR/3 (intervention* OR procedure* OR treatment* OR 'care' OR management OR aid OR program* OR training*)):ti,ab,kw) **AND** ('injury'/de OR 'abdominal injury'/exp OR 'abrasion'/exp OR 'accidental injury'/exp OR 'automutilation'/exp OR 'avulsion injury'/exp OR 'barotrauma'/exp OR 'battered child syndrome'/exp OR 'battle injury'/exp OR 'birth injury'/exp OR 'blood vessel injury'/exp OR 'blunt trauma'/exp OR 'breast injury'/exp OR 'burn'/exp OR 'chemical injury'/exp OR 'childhood injury'/exp OR 'concussion'/exp OR 'contrecoup injury'/exp OR 'contusion'/exp OR 'crush trauma'/exp OR 'drowning'/exp OR 'electric injury'/exp OR 'erosion'/exp OR 'experimental injury'/exp OR 'foreign body'/exp OR 'head and neck injury'/exp OR 'heart injury'/exp OR 'immune injury'/exp OR 'immune mediated injury'/exp OR 'injury scale'/exp OR 'injury severity'/exp OR 'limb injury'/exp OR 'membrane damage'/exp OR 'microtrauma'/exp OR 'multiple trauma'/exp OR 'musculoskeletal injury'/exp OR 'nervous system injury'/exp OR 'organ injury'/exp OR 'pelvis injury'/exp OR 'perforation'/exp OR 'prenatal injury'/exp OR 'psychotrauma'/exp OR 'radiation injury'/exp OR 'reperfusion injury'/exp OR 'respiratory tract injury'/exp OR 'rupture'/exp OR 'seatbelt injury'/exp OR 'skin injury'/exp OR 'soft tissue injury'/exp OR 'strangulation'/exp OR 'surgical injury'/exp OR 'thermal injury'/exp OR 'thorax injury'/exp OR 'tissue injury'/exp OR 'tooth injury'/exp OR 'traumatic amputation'/exp OR 'traumatic hematoma'/exp OR 'traumatic shock'/exp OR 'trench foot'/exp OR 'urogenital tract injury'/exp OR 'volutrauma'/exp OR 'wound'/exp OR (trauma OR wound* OR injur* OR fractur* OR lacerat* OR rupture* OR perforat* OR avulsion* OR contusion* OR barotrauma* OR burn* OR concussion* OR hemorrhage* OR hematoma*):ti,ab,kw)**)**

**OR**

**(**('explosive'/exp OR 'bomb'/exp OR 'weapon of mass destruction'/exp OR 'blast injury'/exp OR 'explosion'/exp OR (APM OR blast OR bomb* OR detonation* OR EED OR ERW OR explosion* OR explosive* OR grenade* OR IED OR landmine* OR 'mine' OR 'mines' OR missile* OR mortar* OR ordnance OR rocket* OR UXB OR UXO):ti,ab,kw) **AND** ((('injury'/de OR 'abdominal injury'/exp OR 'abrasion'/exp OR 'accidental injury'/exp OR 'automutilation'/exp OR 'avulsion injury'/exp OR 'barotrauma'/exp OR 'battered child syndrome'/exp OR 'battle injury'/exp OR 'birth injury'/exp OR 'blood vessel injury'/exp OR 'blunt trauma'/exp OR 'breast injury'/exp OR 'burn'/exp OR 'chemical injury'/exp OR 'childhood injury'/exp OR 'concussion'/exp OR 'contrecoup injury'/exp OR 'contusion'/exp OR 'crush trauma'/exp OR 'drowning'/exp OR 'electric injury'/exp OR 'erosion'/exp OR 'experimental injury'/exp OR 'foreign body'/exp OR 'head and neck injury'/exp OR 'heart injury'/exp OR 'immune injury'/exp OR 'immune mediated injury'/exp OR 'injury scale'/exp OR 'injury severity'/exp OR 'limb injury'/exp OR 'membrane damage'/exp OR 'microtrauma'/exp OR 'multiple trauma'/exp OR 'musculoskeletal injury'/exp OR 'nervous system injury'/exp OR 'organ injury'/exp OR 'pelvis injury'/exp OR 'perforation'/exp OR 'prenatal injury'/exp OR 'psychotrauma'/exp OR 'radiation injury'/exp OR 'reperfusion injury'/exp OR 'respiratory tract injury'/exp OR 'rupture'/exp OR 'seatbelt injury'/exp OR 'skin injury'/exp OR 'soft tissue injury'/exp OR 'strangulation'/exp OR 'surgical injury'/exp OR 'thermal injury'/exp OR 'thorax injury'/exp OR 'tissue injury'/exp OR 'tooth injury'/exp OR 'traumatic amputation'/exp OR 'traumatic hematoma'/exp OR 'traumatic shock'/exp OR 'trench foot'/exp OR 'urogenital tract injury'/exp OR 'volutrauma'/exp OR 'wound'/exp) AND (intervention* OR procedure* OR treatment* OR care OR management OR aid OR program* OR training*):ti,ab,kw) OR (('trauma' OR wound* OR injur* OR fractur* OR lacerat* OR rupture* OR perforat* OR avulsion* OR contusion* OR barotrauma* OR burn* OR concussion* OR hemorrhage* OR hematoma*) NEAR/3 (intervention* OR procedure* OR treatment* OR care OR management OR aid OR program* OR training*)):ti,ab,kw)**))**

**AND**

**(**Afghanistan/exp OR Albania/exp OR Algeria/exp OR 'American Samoa'/exp OR Angola/exp OR 'Antigua and Barbuda'/exp OR Argentina/exp OR Armenia/exp OR Botswana/exp OR Azerbaijan/exp OR Bahrain/exp OR Balkan Peninsula/exp OR Baltic States/exp OR Bangladesh/exp OR Barbados/exp OR Belarus/exp OR Belize/exp OR Benin/exp OR Bhutan/exp OR Bolivia/exp OR 'Bosnia and Herzegovina'/exp OR Botswana/exp OR Brazil/exp OR Bulgaria/exp OR 'Burkina Faso'/exp OR Burundi/exp OR 'Cape Verde'/exp OR Cambodia/exp OR Cameroon/exp OR 'Central African Republic'/exp OR Chad/exp OR Chile/exp OR Colombia/exp OR Comoros/exp OR 'Democratic Republic Congo'/exp OR Congo/exp OR 'Costa Rica'/exp OR 'Cote d`Ivoire'/exp OR Croatia/exp OR Cuba/exp OR Cyprus/exp OR 'Czech Republic'/exp OR Djibouti/exp OR Dominica/exp OR 'Dominican Republic'/exp OR Ecuador/exp OR Egypt/exp OR 'El Salvador'/exp OR 'Equatorial Guinea'/exp OR Eritrea/exp OR Estonia/exp OR Eswatini/exp OR Ethiopia/exp OR Fiji/exp OR Gabon/exp OR Gambia/exp OR 'Georgia (republic)'/exp OR Ghana/exp OR Gibraltar/exp OR Greece/exp OR Grenada/exp OR Guam/exp OR Guatemala/exp OR Guinea/exp OR 'Guinea-Bissau'/exp OR Guyana/exp OR Haiti/exp OR Honduras/exp OR India/exp OR Indonesia/exp OR Iran/exp OR Iraq/exp OR Jamaica/exp OR Jordan/exp OR Kazakhstan/exp OR Kenya/exp OR 'North Korea'/exp OR 'South Korea'/exp OR Kosovo/exp OR Kyrgyzstan/exp OR Laos/exp OR Latvia/exp OR Lebanon/exp OR Lesotho/exp OR Liberia/exp OR 'Libyan Arab Jamahiriya'/exp OR Lithuania/exp OR Macao/exp OR 'Republic of North Macedonia'/exp OR Madagascar/exp OR Malawi/exp OR Malaysia/exp OR Mali/exp OR Malta/exp OR Mayotte/exp OR 'Federated States of Micronesia'/exp OR Palau/exp OR Mauritania/exp OR Mauritius/exp OR Mexico/exp OR Moldova/exp OR Mongolia/exp OR 'Montenegro (republic)'/exp OR Morocco/exp OR Mozambique/exp OR Myanmar/exp OR Namibia/exp OR Nepal/exp OR 'Netherlands Antilles'/exp OR Nicaragua/exp OR Niger/exp OR Nigeria/exp OR Oman/exp OR Pakistan/exp OR Palestine/exp OR Panama/exp OR 'Papua New Guinea'/exp OR Paraguay/exp OR Peru/exp OR Philippines/exp OR Portugal/exp OR 'Puerto Rico'/exp OR Romania/exp OR Rwanda/exp OR Samoa/exp OR 'Sao Tome and Principe'/exp OR Senegal/exp OR Seychelles/exp OR 'Sierra Leone'/exp OR Melanesia/exp OR Somalia/exp OR 'South Africa'/exp OR 'South Sudan'/exp OR Sri Lanka/exp OR 'Saint Kitts and Nevis'/exp OR 'Saint Lucia'/exp OR 'Saint Vincent and the Grenadines'/exp OR Sudan/exp OR Suriname/exp OR Syrian Arab Republic/exp OR Tajikistan/exp OR Tanzania/exp OR Thailand/exp OR 'Timor-Leste'/exp OR Togo/exp OR Tonga/exp OR 'Trinidad and Tobago'/exp OR Tunisia/exp OR Turkmenistan/exp OR Uganda/exp OR Ukraine/exp OR Uruguay/exp OR Uzbekistan/exp OR Vanuatu/exp OR Venezuela/exp OR 'Viet Nam'/exp OR Yemen/exp OR Yugoslavia/exp OR Zambia/exp OR Zimbabwe/exp OR 'Africa south of the Sahara'/exp OR Sahel/exp OR 'Western Sahara'/exp OR 'Central Africa'/exp OR 'North Africa'/exp OR 'Caribbean Islands'/exp OR 'Indian Ocean'/exp OR Caribbean/exp OR 'Caribbean Netherlands'/exp OR 'Central America'/exp OR 'South America'/exp OR 'Central Asia'/exp OR 'Northern Asia'/exp OR 'Southeast Asia'/exp OR 'Western Asia'/de OR 'Middle East'/de OR 'Eastern Europe'/de

OR

(Afghanistan OR Albania OR Algeria OR 'American Samoa' OR Angola OR Antigua OR Barbuda OR Argentina OR 'Argentine Republic' OR Armenia OR Armenian OR Aruba OR Azerbaijan OR Bahrain OR Bangladesh OR Barbados OR Belarus OR Byelarus OR Belorussia OR Byelorussian OR Belize OR 'British Honduras' OR Benin OR Dahomey OR Bhutan OR Bolivia OR Bosnia OR Hercegovina OR Herzegovina OR Botswana OR Bechuanaland OR Brazil OR Brasil OR Bulgaria OR 'Burkina Faso' OR 'Burkina Fasso' OR 'Upper Volta' OR Burundi OR Urundi OR 'Cabo Verde' OR 'Cape Verde' OR Cambodia OR Kampuchea OR 'Khmer Republic' OR Cameroon OR Cameron OR Cameroun OR 'Central African Republic' OR 'Ubangi Shari' OR Chad OR Chile OR Colombia OR Comoros OR Comores OR 'Comoro Islands' OR Mayotte OR Congo OR Zaire OR 'Costa Rica' OR 'Cote D`ivoire' OR 'Cote D` Ivoire' OR 'Cote Divoire' OR 'Cote D Ivoire' OR 'Ivory Coast' OR Croatia OR Cuba OR Cyprus OR 'Czech Republic' OR Czechoslovakia OR Djibouti OR 'French Somaliland' OR Dominica OR 'Dominican Republic' OR Ecuador OR Egypt OR 'United Arab Republic' OR 'El Salvador' OR 'Equatorial Guinea' OR 'Spanish Guinea' OR Eritrea OR Estonia OR Eswatini OR Swaziland OR Ethiopia OR Fiji OR Gabon OR 'Gabonese Republic' OR Gambia OR Georgia OR Georgian OR Ghana OR 'Gold Coast' OR Gibraltar OR Greece OR Grenada OR Guam OR Guatemala OR Guinea OR Guyana OR Guiana OR Haiti OR Hispaniola OR Honduras OR India OR Indonesia OR Timor OR Iran OR Iraq OR 'Isle Of Man' OR Jamaica OR Jordan OR Kazakhstan OR Kazakh OR Kenya OR Korea OR Kosovo OR Kyrgyzstan OR Kirghizia OR Kirgizstan OR 'Kyrgyz Republic' OR Kirghiz OR Laos OR 'Lao Pdr' OR 'Lao People`s Democratic Republic' OR 'Lao Peoples Democratic Republic' OR Latvia OR Lebanon OR Lesotho OR Basutoland OR Liberia OR Libya OR 'Libyan Arab Jamahiriya' OR Lithuania OR Macau OR Macao OR Macedonia OR Madagascar OR 'Malagasy Republic' OR Malawi OR Nyasaland OR Malay OR Malaya OR Malaysia OR Maldives OR 'Indian Ocean' OR Mali OR Malta OR Micronesia OR Kiribati OR 'Marshall Islands' OR Nauru OR 'Northern Mariana Islands' OR Palau OR Tuvalu OR Mauritania OR Mauritius OR Mexico OR Moldova OR Moldovian OR Mongolia OR Montenegro OR Morocco OR Ifni OR Mozambique OR 'Portuguese East Africa' OR Myanmar OR Burma OR Namibia OR Nepal OR 'Netherlands Antilles' OR Nicaragua OR Niger OR Nigeria OR Oman OR Muscat OR Pakistan OR Panama OR 'Papua New Guinea' OR Paraguay OR Peru OR Philippines OR Philipines OR Phillipines OR Phillippines OR Portugal OR 'Portuguese Republic' OR 'Puerto Rico' OR Romania OR Rwanda OR Ruanda OR Samoa OR 'Pacific Islands' OR Polynesia OR 'Samoan Islands' OR 'Sao Tome And Principe' OR Senegal OR Seychelles OR 'Sierra Leone' OR Melanesia OR 'Solomon Island' OR 'Solomon Islands' OR 'Norfolk Island' OR Somalia OR 'South Africa' OR 'South Sudan' OR 'Sri Lanka' OR Ceylon OR 'Saint Kitts And Nevis' OR 'St Kitts And Nevis' OR 'Saint Lucia' OR 'St Lucia' OR 'Saint Vincent' OR 'St Vincent' OR Grenadines OR Sudan OR Suriname OR Surinam OR Syria OR 'Syrian Arab Republic' OR Tajikistan OR Tadjikistan OR Tadzhikistan OR Tadzhik OR Tanzania OR Tanganyika OR Thailand OR Siam OR 'Timor Leste' OR 'East Timor' OR Togo OR 'Togolese Republic' OR Tonga OR Trinidad OR Tobago OR Tunisia OR Turkmenistan OR Turkmen OR Uganda OR Ukraine OR Uruguay OR Uzbekistan OR Uzbek OR Vanuatu OR 'New Hebrides' OR Venezuela OR Vietnam OR 'Viet Nam' OR 'Middle East' OR 'West Bank' OR Gaza OR Palestine OR Yemen OR Yugoslavia OR Zambia OR Zimbabwe OR 'Northern Rhodesia'):ti,ab,kw

OR

('global south' OR 'Africa south of the sahara' OR 'Saharan Africa' OR 'subSaharan Africa' OR 'sub-Saharan Africa' OR 'central Africa' OR 'north Africa' OR 'northern Africa' OR Magreb OR Maghreb OR Sahara OR Sahel OR 'southern Africa' OR 'east Africa' OR 'eastern Africa' OR 'west Africa' OR 'western Africa' OR 'West Indies' OR 'Indian Ocean islands' OR 'Caribbean' OR 'Central America' OR 'Latin America' OR 'South America' OR 'Central Asia' OR 'north Asia' OR 'northern Asia' OR 'southeastern Asia' OR 'south eastern Asia' OR 'southeast Asia' OR 'south east Asia' OR 'western Asia' OR 'east Europe' OR 'eastern Europe'):ti,ab,kw

OR

'developing country'/exp OR 'low income country'/exp OR 'middle income country'/exp OR (((developing OR 'less developed' OR 'lesser developed' OR 'under developed' OR underdeveloped OR 'middle income' OR 'low income' OR 'lower income' OR underserved OR 'under-served' OR deprived OR poor OR poorer) NEAR/1 (country OR countries OR nation OR nations OR world)) OR ((developing OR underdeveloped OR 'middle income' OR 'low income' OR 'lower income' OR underserved OR 'under-served' OR deprived OR poor OR poorer) NEAR/1 (population OR populations)) OR ((developing OR 'less developed' OR underdeveloped OR 'middle income' OR 'low income' OR 'lower income' OR emerging) NEAR/1 (economy OR economies)) OR 'low gdp' OR 'low gnp' OR 'low gross domestic' OR 'low gross national' OR 'lower gdp' OR 'lower gross domestic' OR lmic OR lmics OR 'third world' OR 'lami country' OR 'lami countries' OR 'transitional country' OR 'transitional countries' OR 'emerging nation' OR 'emerging nations'):ti,ab,kw

OR

African/exp OR 'Central Asian'/exp OR 'South Asian'/exp OR 'Southeast Asian'/exp OR 'Caribbean (person)'/exp OR 'Central American'/exp OR 'Melanesian'/exp OR 'Micronesian'/exp OR 'South American'/exp OR

(Afghan OR Afghans OR Afghani OR Albanian OR Albanians OR Algerian OR Algerians OR 'American Samoan' OR 'American Samoans' OR Angolan OR Angolans OR Antiguan OR Antiguans OR Barbudan OR Berbudans OR Argentine OR Argentines OR Argentinian OR Argentinians OR Argentinean OR Argentineans OR Armenian OR Armenians OR Aruban OR Arubans OR Azerbaijani OR Azerbaijanis OR Bahraini OR Bahrainis OR Bangladeshi OR Bangladeshis OR Bangalees OR Bajan OR Bajans OR Barbadian OR Barbadians OR Belarusian OR Belarusians OR Byelorussian OR Byelorussians OR Belizean OR Belizeans OR Beninese OR Benineses OR Bhutanese OR Bolivian OR Bolivians OR Bosnian OR Bosnians OR Botswana OR Batswana OR Brazilian OR Brazilians OR Brasilian OR Brasilians OR Bulgarian OR Bulgarians OR Burkinabe OR Burkinese OR Burundian OR Burundians OR 'Cape Verdean' OR 'Cape Verdeans' OR 'Cabo Verdean' OR 'Cabo Verdeans' OR Cambodian OR Cambodians OR Khmer OR Cameroonian OR Cameroonians OR 'Central African' OR 'Central Africans' OR Chadian OR Chadians OR Chilean OR Chileans OR Colombian OR Colombians OR Comorian OR Comorians OR Congolese OR 'Costa Rican' OR 'Costa Ricans' OR Ivorian OR Ivorians OR Croatian OR Croatians OR Cuban OR Cubans OR Cypriot OR Cypriots OR Czech OR Czechs OR Djiboutian OR Djiboutians OR Dominican OR Dominicans OR Ecuadorian OR Ecuadorians OR Egyptian OR Egyptians OR Salvadoran OR Salvadorans OR 'Equatorial Guinean' OR 'Equatorial Guineans' OR Equatoguinean OR Equatoguineans OR Eritrean OR Eritreans OR Estonian OR Estonians OR Swazi OR Swazis OR Swati OR Swatis OR Ethiopian OR Ethiopians OR Fijian OR Fijians OR Gabonese OR Gabonaise OR Gambian OR Gambians OR Georgian OR Georgians OR Ghanaian OR Ghanaians OR Gibraltarian OR Gibraltarians OR Greek OR Greeks OR Grenadian OR Grenadians OR Guamanian OR Guamanians OR Guatemalan OR Guatemalans OR Guinean OR Guineans OR 'Bissau Guinean' OR 'Bissau Guineans' OR Guyanese OR Haitian OR Haitians OR Honduran OR Hondurans OR Indian OR Indians OR Indonesian OR Indonesians OR Iranian OR Iranians OR Iraqian OR Iraqians OR Iraqi OR Iraqis OR Manx OR Jamaican OR Jamaicans OR Jordanian OR Jordanians OR Kazakhstani OR Kazakhstanis OR Kenyan OR Kenyans OR Kirabati OR Kirabatian OR Kirabatians OR Korean OR Koreans OR Kosovar OR Kosovars OR Kosovan OR Kosovans OR Kyrgyzstani OR Kyrgyzstanis OR Kyrgyz OR Lao OR Laotian OR Laotians OR Latvian OR Latvians OR Lebanese OR Lesothan OR Lesothans OR Lesothonian OR Lesothonians OR Mosotho OR Basotho OR Liberian OR Liberians OR Libyan OR Libyans OR Lithuanian OR Lithuanians OR Macanese OR Macedonian OR Macedonians OR Malagasy OR Madagascan OR Madagascans OR Malawian OR Malawians OR Malay OR Malaya OR Malaysian OR Malaysians OR Maldivian OR Maldivians OR Malian OR Malians OR Maltese OR Marshallese OR Marshalleses OR Mauritanian OR Mauritanians OR Mauritian OR Mauritians OR Mexican OR Mexicans OR Micronesian OR Micronesians OR Moldovan OR Moldovans OR Mongolian OR Mongolians OR Mongol OR Montenegrin OR Montenegrins OR Moroccan OR Moroccans OR Mozambican OR Mozambicans OR Burmese OR Myanma OR Namibian OR Namibians OR Nauruan OR Nauruans OR Nepali OR Nepalese OR 'Netherlands Antillean' OR 'Netherlands Antilleans' OR Nicaraguan OR Nicaraguans OR Nigerien OR Nigeriens OR Nigerian OR Nigerians OR 'Northern Mariana Islander' OR 'Northern Mariana Islanders' OR Mariana OR Marianas OR Omani OR Omanis OR Pakistani OR Pakistanis OR Palauan OR Palauans OR Palestinian OR Palestinians OR Panamanian OR Panamanians OR 'Papua New Guinean' OR 'Papua New Guineans' OR Paraguayan OR Paraguayans OR Peruvian OR Peruvians OR Philippine OR Philippines OR Philipine OR Philipines OR Phillipine OR Phillipines OR Phillippine OR Phillippines OR Filipino OR Filipinos OR Filipina OR Filipinas OR Portuguese OR 'Puerto Rican' OR 'Puerto Ricans' OR Romanian OR Romanians OR Rwandan OR Rwandans OR Rwandese OR Ruandan OR Ruandans OR Ruandese OR Samoan OR Samoans OR Sao Tomean OR Sao Tomeans OR Santomean OR Santomeans OR Senegalese OR Montenegrin OR Montenegrins OR Seychellois OR Seychelloise OR Seychelloises OR 'Sierra Leonean' OR 'Sierra Leoneans' OR 'Solomon Islander' OR 'Solomon Islanders' OR Somali OR Somalis OR 'South African' OR 'South Africans' OR 'South Sudanese' OR 'Sri Lankan' OR 'Sri Lankans' OR Ceylonese OR Kittitian OR Kittitians OR Nevisian OR Nevisians OR 'Saint Lucian' OR 'Saint Lucians' OR Vincentian OR Vincentians OR Sudanese OR Surinamese OR Surinameses OR Syrian OR Syrians OR Tajik OR Tajiks OR Tajikistani OR Tajikistanis OR Tanzanian OR Tanzanians OR Tanganyikan OR Tanganyikans OR Thai OR Timorese OR Timoreses OR Togolese OR Tongan OR Tongans OR Trinidadian OR Trinidadians OR Tobagonian OR Tobagonians OR Tunisian OR Tunisians OR Turkmen OR Turkmens OR Tuvaluan OR Tuvaluans OR Ugandan OR Ugandans OR Ukrainian OR Ukrainians OR Uruguayan OR Uruguayans OR Uzbek OR Uzbeks OR Vanuatu OR Vanuatuan OR Vanuatuans OR Venezuelan OR Venezuelans OR Vietnamese OR Yemeni OR Yemenis OR Yemenite OR Yemenites OR Yemenese OR Yugoslav OR Yugoslavs OR Yugoslavian OR Yugoslavians OR Zambian OR Zambians OR Zimbabwean OR Zimbabweans):ti,ab,kw,exp

**)**

NOT 'plant disease':jt NOT ([animals]/lim NOT [humans]/lim)

AND [2000-2021]/py

**Cumulative Index to Nursing and Allied Health Literature (CINAHL); EBSCO**

|  | **Concept: trauma centers** | **Concept: injuries** |
| --- | --- | --- |
| Subject Headings (MH) | "Emergency Medical Services"  "Emergency Medical Service Communication Systems"  "Emergency Service"  "Poison Control Centers"  "Rescue Work+"  "Sexual Assault Examination"  "Transportation of Patients+"  "Trauma Centers"  "Triage" | "Trauma+"  "Wounds and Injuries"  "Abdominal Injuries+"  "Accidental Injuries"  "Amputation, Traumatic"  "Arm Injuries+"  "Asphyxia"  "Back Injuries+"  "Barotrauma+"  "Birth Injuries+"  "Blast Injuries"  "Burns+"  "Contusions and Abrasions+"  "Crush Injuries"  "Dislocations+"  "Drowning+"  "Electric Injuries+"  "Esophageal Perforation"  "Foreign Bodies+"  "Fractures+"  "Frostbite"  "Hand Injuries+"  "Head Injuries+"  "Heart Injuries+"  "Heat Stress Disorders+"  "Injuries, Self-Inflicted"  "Leg Injuries+"  "Ligament Injuries+"  "Nasal Septal Perforation"  "Neck Injuries+"  "Radiation Injuries+"  "Reinjury"  "Rupture+"  "Shock, Traumatic+"  "Soft Tissue Injuries+"  "Spinal Cord Injuries+"  "Spinal Injuries+"  "Sprains and Strains+"  "Surgical Wound"  "Subluxation"  "Tears and Lacerations+"  "Tendon Injuries+"  "Thoracic Injuries+"  "Tooth Injuries+"  "Toxic Inhalation"  "Tympanic Membrane Perforation"  "Wounds, Nonpenetrating+"  "Wounds, Penetrating+" |
| Free text terms  (searched in Title & Abstract; keyword not a searchable field) | "Emergency Medical Service*"  "Emergency Service*"  "emergency center*"  "emergency centre*"  "emergency health care service*"  "emergency healthcare service*"  "trauma centre*"  "trauma center*"  "trauma system"  "trauma systems"  "trauma care"  "casualty care"  "acute care"  "acute medical care"  "emergency health care"  "emergency medical care"  Emergicent*  **(**(prehospital OR "pre-hospital") N2 (intervention* OR procedure* OR treatment* OR "care" OR management OR aid OR program* OR training*)**)** | trauma  wound*  injur*  fractur*  lacerat*  rupture*  perforat*  avulsion*  contusion*  barotrauma*  burn*  concussion*  hemorrhage*  hematoma* |

|  | **Concept: explosive ordnance** | **Concept: injuries** | **Concept: Medical management** |
| --- | --- | --- | --- |
| Subject Headings (MH) | "Weapons"  "Blast Injuries" | "Trauma+"  "Wounds and Injuries"  "Abdominal Injuries+"  "Accidental Injuries"  "Amputation, Traumatic"  "Arm Injuries+"  "Asphyxia"  "Back Injuries+"  "Barotrauma+"  "Birth Injuries+"  "Blast Injuries"  "Burns+"  "Contusions and Abrasions+"  "Crush Injuries"  "Dislocations+"  "Drowning+"  "Electric Injuries+"  "Esophageal Perforation"  "Foreign Bodies+"  "Fractures+"  "Frostbite"  "Hand Injuries+"  "Head Injuries+"  "Heart Injuries+"  "Heat Stress Disorders+"  "Injuries, Self-Inflicted"  "Leg Injuries+"  "Ligament Injuries+"  "Nasal Septal Perforation"  "Neck Injuries+"  "Occupational-Related Injuries"  "Radiation Injuries+"  "Reinjury"  "Rupture+"  "Shock, Traumatic+"  "Soft Tissue Injuries+"  "Spinal Cord Injuries+"  "Spinal Injuries+"  "Sprains and Strains+"  "Surgical Wound"  "Subluxation"  "Tears and Lacerations+"  "Tendon Injuries+"  "Thoracic Injuries+"  "Tooth Injuries+"  "Toxic Inhalation"  "Tympanic Membrane Perforation"  "Wounds, Nonpenetrating+"  "Wounds, Penetrating+" | (MH "Nursing Interventions") |
| Free text terms  (searched in Title & Abstract; keyword not a searchable field) | APM  blast  bomb*  detonation*  EED  ERW  explosion*  explosive*  IED  grenade*  landmine*  "mine"  "mines"  missile*  mortar*  ordnance  rocket*  UXB  UXO | trauma  wound*  injur*  fractur*  lacerat*  rupture*  perforat*  avulsion*  contusion*  barotrauma*  burn*  concussion*  hemorrhage*  hematoma* | intervention*  procedure*  treatment*  "care"  management  aid  program*  training* |

**((**(MH("Emergency Medical Services" OR "Emergency Medical Service Communication Systems" OR "Emergency Service" OR "Poison Control Centers" OR "Rescue Work+" OR "Sexual Assault Examination" OR "Transportation of Patients+" OR "Trauma Centers" OR "Triage") OR TI ("Emergency Medical Service*" OR "Emergency Service*" OR "emergency center*" OR "emergency centre*" OR "emergency health care service*" OR "emergency healthcare service*" OR "trauma centre*" OR "trauma center*" OR "trauma system" OR "trauma systems" OR "trauma care" OR "casualty care" OR "acute care" OR "acute medical care" OR "emergency health care" OR "emergency medical care" OR Emergicent* OR **(**(prehospital OR "pre-hospital") N2 (intervention* OR procedure* OR treatment* OR "care" OR management OR aid OR program* OR training*)**)**) OR AB ("Emergency Medical Service*" OR "Emergency Service*" OR "emergency center*" OR "emergency centre*" OR "emergency health care service*" OR "emergency healthcare service*" OR "trauma centre*" OR "trauma center*" OR "trauma system" OR "trauma systems" OR "trauma care" OR "casualty care" OR "acute care" OR "acute medical care" OR "emergency health care" OR "emergency medical care" OR Emergicent* OR **(**(prehospital OR "pre-hospital") N2 (intervention* OR procedure* OR treatment* OR "care" OR management OR aid OR program* OR training*)**)**)) **AND** (MH ("Trauma+" OR "Wounds and Injuries" OR "Abdominal Injuries+" OR "Accidental Injuries" OR "Amputation, Traumatic" OR "Arm Injuries+" OR "Asphyxia" OR "Back Injuries+" OR "Barotrauma+" OR "Birth Injuries+" OR "Blast Injuries" OR "Burns+" OR "Contusions and Abrasions+" OR "Crush Injuries" OR "Dislocations+" OR "Drowning+" OR "Electric Injuries+" OR "Esophageal Perforation" OR "Foreign Bodies+" OR "Fractures+" OR "Frostbite" OR "Hand Injuries+" OR "Head Injuries+" OR "Heart Injuries+" OR "Heat Stress Disorders+" OR "Injuries, Self-Inflicted" OR "Leg Injuries+" OR "Ligament Injuries+" OR "Nasal Septal Perforation" OR "Neck Injuries+" OR "Occupational-Related Injuries" OR "Radiation Injuries+" OR "Reinjury" OR "Rupture+" OR "Shock, Traumatic+" OR "Soft Tissue Injuries+" OR "Spinal Cord Injuries+" OR "Spinal Injuries+" OR "Sprains and Strains+" OR "Surgical Wound" OR "Subluxation" OR "Tears and Lacerations+" OR "Tendon Injuries+" OR "Thoracic Injuries+" OR "Tooth Injuries+" OR "Toxic Inhalation" OR "Tympanic Membrane Perforation" OR "Wounds, Nonpenetrating+" OR "Wounds, Penetrating+") OR TI (trauma OR wound* OR injur* OR fractur* OR lacerat* OR rupture* OR perforat* OR avulsion* OR contusion* OR barotrauma* OR burn* OR concussion* OR hemorrhage* OR hematoma*) OR AB (trauma OR wound* OR injur* OR fractur* OR lacerat* OR rupture* OR perforat* OR avulsion* OR contusion* OR barotrauma* OR burn* OR concussion* OR hemorrhage* OR hematoma*))**)**

**OR**

**(**(MH ("Weapons" OR "Blast Injuries") OR TI (APM OR blast OR bomb* OR detonation* OR EED OR ERW OR explosion* OR explosive* OR IED OR grenade* OR landmine* OR "mine" OR "mines" OR missile* OR mortar* OR ordnance OR rocket* OR UXB OR UXO) OR AB (APM OR blast OR bomb* OR detonation* OR EED OR ERW OR explosion* OR explosive* OR IED OR grenade* OR landmine* OR "mine" OR "mines" OR missile* OR mortar* OR ordnance OR rocket* OR UXB OR UXO)) **AND** (MH ("Trauma+" OR "Wounds and Injuries" OR "Abdominal Injuries+" OR "Accidental Injuries" OR "Amputation, Traumatic" OR "Arm Injuries+" OR "Asphyxia" OR "Back Injuries+" OR "Barotrauma+" OR "Birth Injuries+" OR "Blast Injuries" OR "Burns+" OR "Contusions and Abrasions+" OR "Crush Injuries" OR "Dislocations+" OR "Drowning+" OR "Electric Injuries+" OR "Esophageal Perforation" OR "Foreign Bodies+" OR "Fractures+" OR "Frostbite" OR "Hand Injuries+" OR "Head Injuries+" OR "Heart Injuries+" OR "Heat Stress Disorders+" OR "Injuries, Self-Inflicted" OR "Leg Injuries+" OR "Ligament Injuries+" OR "Nasal Septal Perforation" OR "Neck Injuries+" OR "Occupational-Related Injuries" OR "Radiation Injuries+" OR "Reinjury" OR "Rupture+" OR "Shock, Traumatic+" OR "Soft Tissue Injuries+" OR "Spinal Cord Injuries+" OR "Spinal Injuries+" OR "Sprains and Strains+" OR "Surgical Wound" OR "Subluxation" OR "Tears and Lacerations+" OR "Tendon Injuries+" OR "Thoracic Injuries+" OR "Tooth Injuries+" OR "Toxic Inhalation" OR "Tympanic Membrane Perforation" OR "Wounds, Nonpenetrating+" OR "Wounds, Penetrating+") OR TI (trauma OR wound* OR injur* OR fractur* OR lacerat* OR rupture* OR perforat* OR avulsion* OR contusion* OR barotrauma* OR burn* OR concussion* OR hemorrhage* OR hematoma*) OR AB (trauma OR wound* OR injur* OR fractur* OR lacerat* OR rupture* OR perforat* OR avulsion* OR contusion* OR barotrauma* OR burn* OR concussion* OR hemorrhage* OR hematoma*)) **AND** ((MH "Nursing Interventions") OR TI (intervention* OR procedure* OR treatment* OR "care" OR management OR aid OR program* OR training*) OR AB (intervention* OR procedure* OR treatment* OR "care" OR management OR aid OR program* OR training*))**))**

**AND**

**(**MH ("Afghanistan" OR "Albania" OR "Algeria" OR "American Samoa" OR "Angola" OR "Antigua" OR "Argentina" OR "Armenia" OR "Aruba" OR "Azerbaijan" OR "Bahrain" OR "Bangladesh" OR "Barbados" OR "Byelarus" OR "Belize" OR "Benin" OR "Bhutan" OR "Bolivia" OR "Bosnia-Herzegovina" OR "Botswana" OR "Brazil" OR "Bulgaria" OR "Burkina Faso" OR "Burundi" OR "Cape Verde" OR "Cambodia" OR "Cameroon" OR "Central African Republic" OR "Chad" OR "Chile" OR "Colombia" OR "Democratic Republic of the Congo" OR "Congo" OR "Costa Rica" OR "Cote d'Ivoire" OR "Croatia" OR "Cuba" OR "Czech Republic" OR "Djibouti" OR "Dominica" OR "Dominican Republic" OR "Ecuador" OR "Egypt" OR "El Salvador" OR "Equatorial Guinea" OR "Eritrea" OR "Estonia" OR "Swaziland" OR "Ethiopia" OR "Gabon" OR "Gambia" OR "Georgia (Republic)" OR "Ghana" OR "Gibraltar" OR "Greece" OR "Guam" OR "Guatemala" OR "Guinea" OR "Guinea-Bissau" OR "Guyana" OR "Haiti" OR "Honduras" OR "India" OR "Indonesia" OR "Iran" OR "Iraq" OR "Jamaica" OR "Jordan" OR "Kazakhstan" OR "Kenya" OR "North Korea" OR "South Korea" OR "Kyrgyzstan" OR "Laos" OR "Latvia" OR "Lebanon" OR "Lesotho" OR "Liberia" OR "Libya" OR "Lithuania" OR "Macao" OR "Macedonia (Republic)" OR "Madagascar" OR "Malawi" OR "Malaysia" OR "Mali" OR "Melanesia+" OR "Micronesia" OR "Mauritania" OR "Mexico" OR "Moldova" OR "Mongolia" OR "Morocco" OR "Mozambique" OR "Myanmar" OR "Namibia" OR "Nepal" OR "Netherlands Antilles" OR "Nicaragua" OR "Niger" OR "Nigeria" OR "Oman" OR "Pakistan" OR "Panama+" OR "Papua New Guinea" OR "Paraguay" OR "Peru" OR "Philippines" OR "Portugal" OR "Puerto Rico" OR "Romania" OR "Rwanda" OR "Independent State of Samoa" OR "Senegal" OR "Sierra Leone" OR "Somalia" OR "South Africa" OR "Sri Lanka" OR "Sudan" OR "Suriname" OR "Syria" OR "Tajikistan" OR "Tanzania" OR "Thailand" OR "Timor" OR "East Timor" OR "Togo" OR "Trinidad and Tobago" OR "Tunisia" OR "Turkmenistan" OR "Uganda" OR "Ukraine" OR "Uruguay" OR "Uzbekistan" OR "Venezuela" OR "Vietnam" OR "Yemen" OR "Yugoslavia+" OR "Zambia" OR "Zimbabwe" OR "Africa+" OR "Africa South of the Sahara" OR "Africa, Central+" OR "Africa, Northern+" OR "Africa, Southern+" OR "Africa, Eastern+" OR "Africa, Western+" OR "West Indies+" OR "Indian Ocean Islands+" OR "Central America+" OR "Latin America" OR "South America" OR "Asia, Central+" OR "Asia, Southeastern" OR "Asia, Western" OR "Middle East" OR "Europe, Eastern" OR "Baltic States+" OR "Mediterranean Islands" OR "Pacific Islands")

OR

TI (Afghanistan OR Albania OR Algeria OR American Samoa OR Angola OR Antigua OR Barbuda OR Argentina OR "Argentine Republic" OR Armenia OR Armenian OR Aruba OR Azerbaijan OR Bahrain OR Bangladesh OR Barbados OR Belarus OR Byelarus OR Belorussia OR Byelorussian OR Belize OR British Honduras OR Benin OR Dahomey OR Bhutan OR Bolivia OR Bosnia OR Hercegovina OR Herzegovina OR Botswana OR Bechuanaland OR Brazil OR Brasil OR Bulgaria OR Burkina Faso OR Burkina Fasso OR Upper Volta OR Burundi OR Urundi OR Cabo Verde OR Cape Verde OR Cambodia OR Kampuchea OR Khmer Republic OR Cameroon OR Cameron OR Cameroun OR Central African Republic OR Ubangi Shari OR Chad OR Chile OR Colombia OR Comoros OR Comores OR Comoro Islands OR Mayotte OR Congo OR Zaire OR Costa Rica OR Cote D’ivoire OR Cote D’ Ivoire OR Cote Divoire OR Cote D Ivoire OR **Côte d'Ivoire** OR Ivory Coast OR Croatia OR Cuba OR Cyprus OR Czech Republic OR Czechoslovakia OR Djibouti OR French Somaliland OR Dominica OR Dominican Republic OR Ecuador OR Egypt OR United Arab Republic OR El Salvador OR Equatorial Guinea OR Spanish Guinea OR Eritrea OR Estonia OR Eswatini OR Swaziland OR Ethiopia OR Fiji OR Gabon OR Gabonese Republic OR Gambia OR Georgia OR Georgian OR Ghana OR Gold Coast OR Gibraltar OR Greece OR Grenada OR Guam OR Guatemala OR Guinea OR Guyana OR Guiana OR Haiti OR Hispaniola OR Honduras OR India OR Indonesia OR Timor OR Iran OR Iraq OR Isle Of Man OR Jamaica OR Jordan OR Kazakhstan OR Kazakh OR Kenya OR Korea OR Kosovo OR Kyrgyzstan OR Kirghizia OR Kirgizstan OR Kyrgyz Republic OR Kirghiz OR Laos OR Lao Pdr OR Lao People's Democratic Republic OR Latvia OR Lebanon OR Lesotho OR Basutoland OR Liberia OR Libya OR Libyan Arab Jamahiriya OR Lithuania OR Macau OR Macao OR Macedonia OR Madagascar OR Malagasy Republic OR Malawi OR Nyasaland OR Malaysia OR Maldives OR Indian Ocean OR Mali OR Malta OR Micronesia OR Kiribati OR Marshall Islands OR Nauru OR Northern Mariana Islands OR Palau OR Tuvalu OR Mauritania OR Mauritius OR Mexico OR Moldova OR Moldovian OR Mongolia OR Montenegro OR Morocco OR Ifni OR Mozambique OR Portuguese East Africa OR Myanmar OR Burma OR Namibia OR Nepal OR Netherlands Antilles OR Nicaragua OR Niger OR Nigeria OR Oman OR Muscat OR Pakistan OR Panama OR Papua New Guinea OR Paraguay OR Peru OR Philippines OR Philipines OR Phillipines OR Phillippines OR Portugal OR Portuguese Republic OR Puerto Rico OR Romania OR Rwanda OR Ruanda OR Samoa OR Pacific Islands OR Polynesia OR Samoan Islands OR Sao Tome And Principe OR Senegal OR Seychelles OR Sierra Leone OR Melanesia OR Solomon Island OR Solomon Islands OR Norfolk Island OR Somalia OR South Africa OR South Sudan OR Sri Lanka OR Ceylon OR Saint Kitts And Nevis OR St Kitts And Nevis OR Saint Lucia OR St Lucia OR Saint Vincent OR St Vincent OR Grenadines OR Sudan OR Suriname OR Surinam OR Syria OR Syrian Arab Republic OR Tajikistan OR Tadjikistan OR Tadzhikistan OR Tadzhik OR Tanzania OR Tanganyika OR Thailand OR Siam OR Timor Leste OR East Timor OR Togo OR Togolese Republic OR Tonga OR Trinidad OR Tobago OR Tunisia OR Turkmenistan OR Turkmen OR Uganda OR Ukraine OR Uruguay OR Uzbekistan OR Uzbek OR Vanuatu OR New Hebrides OR Venezuela OR Vietnam OR Viet Nam OR West Bank OR Gaza OR Palestine OR Yemen OR Yugoslavia OR Zambia OR Zimbabwe OR Northern Rhodesia)

OR

AB (Afghanistan OR Albania OR Algeria OR American Samoa OR Angola OR Antigua OR Barbuda OR Argentina OR "Argentine Republic" OR Armenia OR Armenian OR Aruba OR Azerbaijan OR Bahrain OR Bangladesh OR Barbados OR Belarus OR Byelarus OR Belorussia OR Byelorussian OR Belize OR British Honduras OR Benin OR Dahomey OR Bhutan OR Bolivia OR Bosnia OR Hercegovina OR Herzegovina OR Botswana OR Bechuanaland OR Brazil OR Brasil OR Bulgaria OR Burkina Faso OR Burkina Fasso OR Upper Volta OR Burundi OR Urundi OR Cabo Verde OR Cape Verde OR Cambodia OR Kampuchea OR Khmer Republic OR Cameroon OR Cameron OR Cameroun OR Central African Republic OR Ubangi Shari OR Chad OR Chile OR Colombia OR Comoros OR Comores OR Comoro Islands OR Mayotte OR Congo OR Zaire OR Costa Rica OR Cote D’ivoire OR Cote D’ Ivoire OR Cote Divoire OR Cote D Ivoire OR **Côte d'Ivoire** OR Ivory Coast OR Croatia OR Cuba OR Cyprus OR Czech Republic OR Czechoslovakia OR Djibouti OR French Somaliland OR Dominica OR Dominican Republic OR Ecuador OR Egypt OR United Arab Republic OR El Salvador OR Equatorial Guinea OR Spanish Guinea OR Eritrea OR Estonia OR Eswatini OR Swaziland OR Ethiopia OR Fiji OR Gabon OR Gabonese Republic OR Gambia OR Georgia OR Georgian OR Ghana OR Gold Coast OR Gibraltar OR Greece OR Grenada OR Guam OR Guatemala OR Guinea OR Guyana OR Guiana OR Haiti OR Hispaniola OR Honduras OR India OR Indonesia OR Timor OR Iran OR Iraq OR Isle Of Man OR Jamaica OR Jordan OR Kazakhstan OR Kazakh OR Kenya OR Korea OR Kosovo OR Kyrgyzstan OR Kirghizia OR Kirgizstan OR Kyrgyz Republic OR Kirghiz OR Laos OR Lao Pdr OR Lao People's Democratic Republic OR Latvia OR Lebanon OR Lesotho OR Basutoland OR Liberia OR Libya OR Libyan Arab Jamahiriya OR Lithuania OR Macau OR Macao OR Macedonia OR Madagascar OR Malagasy Republic OR Malawi OR Nyasaland OR Malaysia OR Maldives OR Indian Ocean OR Mali OR Malta OR Micronesia OR Kiribati OR Marshall Islands OR Nauru OR Northern Mariana Islands OR Palau OR Tuvalu OR Mauritania OR Mauritius OR Mexico OR Moldova OR Moldovian OR Mongolia OR Montenegro OR Morocco OR Ifni OR Mozambique OR Portuguese East Africa OR Myanmar OR Burma OR Namibia OR Nepal OR Netherlands Antilles OR Nicaragua OR Niger OR Nigeria OR Oman OR Muscat OR Pakistan OR Panama OR Papua New Guinea OR Paraguay OR Peru OR Philippines OR Philipines OR Phillipines OR Phillippines OR Portugal OR Portuguese Republic OR Puerto Rico OR Romania OR Rwanda OR Ruanda OR Samoa OR Pacific Islands OR Polynesia OR Samoan Islands OR Sao Tome And Principe OR Senegal OR Seychelles OR Sierra Leone OR Melanesia OR Solomon Island OR Solomon Islands OR Norfolk Island OR Somalia OR South Africa OR South Sudan OR Sri Lanka OR Ceylon OR Saint Kitts And Nevis OR St Kitts And Nevis OR Saint Lucia OR St Lucia OR Saint Vincent OR St Vincent OR Grenadines OR Sudan OR Suriname OR Surinam OR Syria OR Syrian Arab Republic OR Tajikistan OR Tadjikistan OR Tadzhikistan OR Tadzhik OR Tanzania OR Tanganyika OR Thailand OR Siam OR Timor Leste OR East Timor OR Togo OR Togolese Republic OR Tonga OR Trinidad OR Tobago OR Tunisia OR Turkmenistan OR Turkmen OR Uganda OR Ukraine OR Uruguay OR Uzbekistan OR Uzbek OR Vanuatu OR New Hebrides OR Venezuela OR Vietnam OR Viet Nam OR West Bank OR Gaza OR Palestine OR Yemen OR Yugoslavia OR Zambia OR Zimbabwe OR Northern Rhodesia)

OR

TI ("global south" OR Africa* OR Magreb OR Maghreb OR Sahara OR Sahel OR "West Indies" OR "Indian Ocean islands" OR Caribbean OR "Central America*" OR "Latin America*" OR "South America*" OR "Central Asia*" OR "north Asia*" OR "northern Asia*" OR "southeastern Asia*" OR "south eastern Asia*" OR "southeast Asia*" OR "south east Asia*" OR "western Asia*" OR "Middle East*" OR "east Europe*" OR "eastern Europe*")

OR

AB ("global south" OR Africa* OR Magreb OR Maghreb OR Sahara OR Sahel OR "West Indies" OR "Indian Ocean islands" OR Caribbean OR "Central America*" OR "Latin America*" OR "South America*" OR "Central Asia*" OR "north Asia*" OR "northern Asia*" OR "southeastern Asia*" OR "south eastern Asia*" OR "southeast Asia*" OR "south east Asia*" OR "western Asia*" OR "Middle East*" OR "east Europe*" OR "eastern Europe*")

OR

MH ("Developing Countries" OR "Low and Middle Income Countries")

OR

AB ("global south" OR Africa* OR Magreb OR Maghreb OR Sahara OR Sahel OR "West Indies" OR "Indian Ocean islands" OR Caribbean OR "Central America*" OR "Latin America*" OR "South America*" OR "Central Asia*" OR "north Asia*" OR "northern Asia*" OR "southeastern Asia*" OR "south eastern Asia*" OR "southeast Asia*" OR "south east Asia*" OR "western Asia*" OR "Middle East*" OR "east Europe*" OR "eastern Europe*")

OR

TI ("developing country" OR "developing countries" OR "developing nation" OR "developing nations" OR "developing population" OR "developing populations" OR "developing world" OR "less developed country" OR "less developed countries" OR "less developed nation" OR "less developed nations" OR "less developed world" OR "lesser developed countries" OR "lesser developed nations" OR "under developed country" OR "under developed countries" OR "under developed nations" OR "under developed world" OR "underdeveloped country" OR "underdeveloped countries" OR "underdeveloped nation" OR "underdeveloped nations" OR "underdeveloped population" OR "underdeveloped populations" OR "underdeveloped world" OR "middle income country" OR "middle income countries" OR "middle income nation" OR "middle income nations" OR "middle income population" OR "middle income populations" OR "low income country" OR "low income countries" OR "low income nation" OR "low income nations" OR "low income population" OR "low income populations" OR "lower income country" OR "lower income countries" OR "lower income nations" OR "lower income population" OR "lower income populations" OR "underserved countries" OR "underserved nations" OR "underserved population" OR "underserved populations" OR "under served population" OR "under served populations" OR "deprived countries" OR "deprived population" OR "deprived populations" OR "poor country" OR "poor countries" OR "poor nation" OR "poor nations" OR "poor population" OR "poor populations" OR "poor world" OR "poorer countries" OR "poorer nations" OR "poorer population" OR "poorer populations" OR "developing economy" OR "developing economies" OR "less developed economy" OR "less developed economies" OR "underdeveloped economies" OR "middle income economy" OR "middle income economies" OR "low income economy" OR "low income economies" OR "lower income economies" OR "low gdp" OR "low gnp" OR "low gross domestic" OR "low gross national" OR "lower gdp" OR "lower gross domestic" OR lmic OR lmics OR "third world" OR "lami country" OR "lami countries" OR "transitional country" OR "transitional countries" OR "emerging economies" OR "emerging nation" OR "emerging nations" OR "low resource" OR austere)

OR

AB ("developing country" OR "developing countries" OR "developing nation" OR "developing nations" OR "developing population" OR "developing populations" OR "developing world" OR "less developed country" OR "less developed countries" OR "less developed nation" OR "less developed nations" OR "less developed world" OR "lesser developed countries" OR "lesser developed nations" OR "under developed country" OR "under developed countries" OR "under developed nations" OR "under developed world" OR "underdeveloped country" OR "underdeveloped countries" OR "underdeveloped nation" OR "underdeveloped nations" OR "underdeveloped population" OR "underdeveloped populations" OR "underdeveloped world" OR "middle income country" OR "middle income countries" OR "middle income nation" OR "middle income nations" OR "middle income population" OR "middle income populations" OR "low income country" OR "low income countries" OR "low income nation" OR "low income nations" OR "low income population" OR "low income populations" OR "lower income country" OR "lower income countries" OR "lower income nations" OR "lower income population" OR "lower income populations" OR "underserved countries" OR "underserved nations" OR "underserved population" OR "underserved populations" OR "under served population" OR "under served populations" OR "deprived countries" OR "deprived population" OR "deprived populations" OR "poor country" OR "poor countries" OR "poor nation" OR "poor nations" OR "poor population" OR "poor populations" OR "poor world" OR "poorer countries" OR "poorer nations" OR "poorer population" OR "poorer populations" OR "developing economy" OR "developing economies" OR "less developed economy" OR "less developed economies" OR "underdeveloped economies" OR "middle income economy" OR "middle income economies" OR "low income economy" OR "low income economies" OR "lower income economies" OR "low gdp" OR "low gnp" OR "low gross domestic" OR "low gross national" OR "lower gdp" OR "lower gross domestic" OR lmic OR lmics OR "third world" OR "lami country" OR "lami countries" OR "transitional country" OR "transitional countries" OR "emerging economies" OR "emerging nation" OR "emerging nations" OR "low resource" OR austere)

OR

MH ("Cambodians" OR "Filipinos" OR "Koreans" OR "Laotians" OR "Thais" OR "Vietnamese")

OR

TI ("Afghan" OR "Afghans" OR "Afghani" OR "Albanian" OR "Albanians" OR "Algerian" OR "Algerians" OR "American Samoan" OR "American Samoans" OR "Angolan" OR "Angolans" OR "Antiguan" OR "Antiguans" OR "Barbudan" OR "Berbudans" OR "Argentine" OR "Argentines" OR "Argentinian" OR "Argentinians" OR "Argentinean" OR "Argentineans" OR "Armenian" OR "Armenians" OR "Aruban" OR "Arubans" OR "Azerbaijani" OR "Azerbaijanis" OR "Bahraini" OR "Bahrainis" OR "Bangladeshi" OR "Bangladeshis" OR "Bangalees" OR "Bajan" OR "Bajans" OR "Barbadian" OR "Barbadians" OR "Belarusian" OR "Belarusians" OR "Byelorussian" OR "Byelorussians" OR "Belizean" OR "Belizeans" OR "Beninese" OR "Benineses" OR "Bhutanese" OR "Bolivian" OR "Bolivians" OR "Bosnian" OR "Bosnians" OR "Botswana" OR "Batswana" OR "Brazilian" OR "Brazilians" OR "Brasilian" OR "Brasilians" OR "Bulgarian" OR "Bulgarians" OR "Burkinabe" OR "Burkinese" OR "Burundian" OR "Burundians" OR "Cape Verdean" OR "Cape Verdeans" OR "Cabo Verdean" OR "Cabo Verdeans" OR "Cambodian" OR "Cambodians" OR "Khmer" OR "Cameroonian" OR "Cameroonians" OR "Central African" OR "Central Africans" OR "Chadian" OR "Chadians" OR "Chilean" OR "Chileans" OR "Colombian" OR "Colombians" OR "Comorian" OR "Comorians" OR "Congolese" OR "Costa Rican" OR "Costa Ricans" OR "Ivorian" OR "Ivorians" OR "Croatian" OR "Croatians" OR "Cuban" OR "Cubans" OR "Cypriot" OR "Cypriots" OR "Czech" OR "Czechs" OR "Djiboutian" OR "Djiboutians" OR "Dominican" OR "Dominicans" OR "Ecuadorian" OR "Ecuadorians" OR "Egyptian" OR "Egyptians" OR "Salvadoran" OR "Salvadorans" OR "Equatorial Guinean" OR "Equatorial Guineans" OR "Equatoguinean" OR "Equatoguineans" OR "Eritrean" OR "Eritreans" OR "Estonian" OR "Estonians" OR "Swazi" OR "Swazis" OR "Swati" OR "Swatis" OR "Ethiopian" OR "Ethiopians" OR "Fijian" OR "Fijians" OR "Gabonese" OR "Gabonaise" OR "Gambian" OR "Gambians" OR "Georgian" OR "Georgians" OR "Ghanaian" OR "Ghanaians" OR "Gibraltarian" OR "Gibraltarians" OR "Greek" OR "Greeks" OR "Grenadian" OR "Grenadians" OR "Guamanian" OR "Guamanians" OR "Guatemalan" OR "Guatemalans" OR "Guinean" OR "Guineans" OR "Bissau Guinean" OR "Bissau Guineans" OR "Guyanese" OR "Haitian" OR "Haitians" OR "Honduran" OR "Hondurans" OR "Indian" OR "Indians" OR "Indonesian" OR "Indonesians" OR "Iranian" OR "Iranians" OR "Iraqian" OR "Iraqians" OR "Iraqi" OR "Iraqis" OR "Manx" OR "Jamaican" OR "Jamaicans" OR "Jordanian" OR "Jordanians" OR "Kazakhstani" OR "Kazakhstanis" OR "Kenyan" OR "Kenyans" OR "Kirabati" OR "Kirabatian" OR "Kirabatians" OR "Korean" OR "Koreans" OR "Kosovar" OR "Kosovars" OR "Kosovan" OR "Kosovans" OR "Kyrgyzstani" OR "Kyrgyzstanis" OR "Kyrgyz" OR "Lao" OR "Laotian" OR "Laotians" OR "Latvian" OR "Latvians" OR "Lebanese" OR "Lesothan" OR "Lesothans" OR "Lesothonian" OR "Lesothonians" OR "Mosotho" OR "Basotho" OR "Liberian" OR "Liberians" OR "Libyan" OR "Libyans" OR "Lithuanian" OR "Lithuanians" OR "Macanese" OR "Macedonian" OR "Macedonians" OR "Malagasy" OR "Madagascan" OR "Madagascans" OR "Malawian" OR "Malawians" OR "Malay" OR "Malaya" OR "Malaysian" OR "Malaysians" OR "Maldivian" OR "Maldivians" OR "Malian" OR "Malians" OR "Maltese" OR "Marshallese" OR "Marshalleses" OR "Mauritanian" OR "Mauritanians" OR "Mauritian" OR "Mauritians" OR "Mexican" OR "Mexicans" OR "Micronesian" OR "Micronesians" OR "Moldovan" OR "Moldovans" OR "Mongolian" OR "Mongolians" OR "Mongol" OR "Montenegrin" OR "Montenegrins" OR "Moroccan" OR "Moroccans" OR "Mozambican" OR "Mozambicans" OR "Burmese" OR "Myanma" OR "Namibian" OR "Namibians" OR "Nauruan" OR "Nauruans" OR "Nepali" OR "Nepalese" OR "Netherlands Antillean" OR "Netherlands Antilleans" OR "Nicaraguan" OR "Nicaraguans" OR "Nigerien" OR "Nigeriens" OR "Nigerian" OR "Nigerians" OR "Northern Mariana Islander" OR "Northern Mariana Islanders" OR "Mariana" OR "Marianas" OR "Omani" OR "Omanis" OR "Pakistani" OR "Pakistanis" OR "Palauan" OR "Palauans" OR "Palestinian" OR "Palestinians" OR "Panamanian" OR "Panamanians" OR "Papua New Guinean" OR "Papua New Guineans" OR "Paraguayan" OR "Paraguayans" OR "Peruvian" OR "Peruvians" OR "Philippine" OR "Philippines" OR "Philipine" OR "Philipines" OR "Phillipine" OR "Phillipines" OR "Phillippine" OR "Phillippines" OR "Filipino" OR "Filipinos" OR "Filipina" OR "Filipinas" OR "Portuguese" OR "Puerto Rican" OR "Puerto Ricans" OR "Romanian" OR "Romanians" OR "Rwandan" OR "Rwandans" OR "Rwandese" OR "Ruandan" OR "Ruandans" OR "Ruandese" OR "Samoan" OR "Samoans" OR "Sao Tomean" OR "Sao Tomeans" OR "Santomean" OR "Santomeans" OR "Senegalese" OR "Montenegrin" OR "Montenegrins" OR "Seychellois" OR "Seychelloise" OR "Seychelloises" OR "Sierra Leonean" OR "Sierra Leoneans" OR "Solomon Islander" OR "Solomon Islanders" OR "Somali" OR "Somalis" OR "South African" OR "South Africans" OR "South Sudanese" OR "Sri Lankan" OR "Sri Lankans" OR "Ceylonese" OR "Kittitian" OR "Kittitians" OR "Nevisian" OR "Nevisians" OR "Saint Lucian" OR "Saint Lucians" OR "Vincentian" OR "Vincentians" OR "Sudanese" OR "Surinamese" OR "Surinameses" OR "Syrian" OR "Syrians" OR "Tajik" OR "Tajiks" OR "Tajikistani" OR "Tajikistanis" OR "Tanzanian" OR "Tanzanians" OR "Tanganyikan" OR "Tanganyikans" OR "Thai" OR "Timorese" OR "Timoreses" OR "Togolese" OR "Tongan" OR "Tongans" OR "Trinidadian" OR "Trinidadians" OR "Tobagonian" OR "Tobagonians" OR "Tunisian" OR "Tunisians" OR "Turkmen" OR "Turkmens" OR "Tuvaluan" OR "Tuvaluans" OR "Ugandan" OR "Ugandans" OR "Ukrainian" OR "Ukrainians" OR "Uruguayan" OR "Uruguayans" OR "Uzbek" OR "Uzbeks" OR "Vanuatu" OR "Vanuatuan" OR "Vanuatuans" OR "Venezuelan" OR "Venezuelans" OR "Vietnamese" OR "Yemeni" OR "Yemenis" OR "Yemenite" OR "Yemenites" OR "Yemenese" OR "Yugoslav" OR "Yugoslavs" OR "Yugoslavian" OR "Yugoslavians" OR "Zambian" OR "Zambians" OR "Zimbabwean" OR "Zimbabweans")

OR

AB ("Afghan" OR "Afghans" OR "Afghani" OR "Albanian" OR "Albanians" OR "Algerian" OR "Algerians" OR "American Samoan" OR "American Samoans" OR "Angolan" OR "Angolans" OR "Antiguan" OR "Antiguans" OR "Barbudan" OR "Berbudans" OR "Argentine" OR "Argentines" OR "Argentinian" OR "Argentinians" OR "Argentinean" OR "Argentineans" OR "Armenian" OR "Armenians" OR "Aruban" OR "Arubans" OR "Azerbaijani" OR "Azerbaijanis" OR "Bahraini" OR "Bahrainis" OR "Bangladeshi" OR "Bangladeshis" OR "Bangalees" OR "Bajan" OR "Bajans" OR "Barbadian" OR "Barbadians" OR "Belarusian" OR "Belarusians" OR "Byelorussian" OR "Byelorussians" OR "Belizean" OR "Belizeans" OR "Beninese" OR "Benineses" OR "Bhutanese" OR "Bolivian" OR "Bolivians" OR "Bosnian" OR "Bosnians" OR "Botswana" OR "Batswana" OR "Brazilian" OR "Brazilians" OR "Brasilian" OR "Brasilians" OR "Bulgarian" OR "Bulgarians" OR "Burkinabe" OR "Burkinese" OR "Burundian" OR "Burundians" OR "Cape Verdean" OR "Cape Verdeans" OR "Cabo Verdean" OR "Cabo Verdeans" OR "Cambodian" OR "Cambodians" OR "Khmer" OR "Cameroonian" OR "Cameroonians" OR "Central African" OR "Central Africans" OR "Chadian" OR "Chadians" OR "Chilean" OR "Chileans" OR "Colombian" OR "Colombians" OR "Comorian" OR "Comorians" OR "Congolese" OR "Costa Rican" OR "Costa Ricans" OR "Ivorian" OR "Ivorians" OR "Croatian" OR "Croatians" OR "Cuban" OR "Cubans" OR "Cypriot" OR "Cypriots" OR "Czech" OR "Czechs" OR "Djiboutian" OR "Djiboutians" OR "Dominican" OR "Dominicans" OR "Ecuadorian" OR "Ecuadorians" OR "Egyptian" OR "Egyptians" OR "Salvadoran" OR "Salvadorans" OR "Equatorial Guinean" OR "Equatorial Guineans" OR "Equatoguinean" OR "Equatoguineans" OR "Eritrean" OR "Eritreans" OR "Estonian" OR "Estonians" OR "Swazi" OR "Swazis" OR "Swati" OR "Swatis" OR "Ethiopian" OR "Ethiopians" OR "Fijian" OR "Fijians" OR "Gabonese" OR "Gabonaise" OR "Gambian" OR "Gambians" OR "Georgian" OR "Georgians" OR "Ghanaian" OR "Ghanaians" OR "Gibraltarian" OR "Gibraltarians" OR "Greek" OR "Greeks" OR "Grenadian" OR "Grenadians" OR "Guamanian" OR "Guamanians" OR "Guatemalan" OR "Guatemalans" OR "Guinean" OR "Guineans" OR "Bissau Guinean" OR "Bissau Guineans" OR "Guyanese" OR "Haitian" OR "Haitians" OR "Honduran" OR "Hondurans" OR "Indian" OR "Indians" OR "Indonesian" OR "Indonesians" OR "Iranian" OR "Iranians" OR "Iraqian" OR "Iraqians" OR "Iraqi" OR "Iraqis" OR "Manx" OR "Jamaican" OR "Jamaicans" OR "Jordanian" OR "Jordanians" OR "Kazakhstani" OR "Kazakhstanis" OR "Kenyan" OR "Kenyans" OR "Kirabati" OR "Kirabatian" OR "Kirabatians" OR "Korean" OR "Koreans" OR "Kosovar" OR "Kosovars" OR "Kosovan" OR "Kosovans" OR "Kyrgyzstani" OR "Kyrgyzstanis" OR "Kyrgyz" OR "Lao" OR "Laotian" OR "Laotians" OR "Latvian" OR "Latvians" OR "Lebanese" OR "Lesothan" OR "Lesothans" OR "Lesothonian" OR "Lesothonians" OR "Mosotho" OR "Basotho" OR "Liberian" OR "Liberians" OR "Libyan" OR "Libyans" OR "Lithuanian" OR "Lithuanians" OR "Macanese" OR "Macedonian" OR "Macedonians" OR "Malagasy" OR "Madagascan" OR "Madagascans" OR "Malawian" OR "Malawians" OR "Malay" OR "Malaya" OR "Malaysian" OR "Malaysians" OR "Maldivian" OR "Maldivians" OR "Malian" OR "Malians" OR "Maltese" OR "Marshallese" OR "Marshalleses" OR "Mauritanian" OR "Mauritanians" OR "Mauritian" OR "Mauritians" OR "Mexican" OR "Mexicans" OR "Micronesian" OR "Micronesians" OR "Moldovan" OR "Moldovans" OR "Mongolian" OR "Mongolians" OR "Mongol" OR "Montenegrin" OR "Montenegrins" OR "Moroccan" OR "Moroccans" OR "Mozambican" OR "Mozambicans" OR "Burmese" OR "Myanma" OR "Namibian" OR "Namibians" OR "Nauruan" OR "Nauruans" OR "Nepali" OR "Nepalese" OR "Netherlands Antillean" OR "Netherlands Antilleans" OR "Nicaraguan" OR "Nicaraguans" OR "Nigerien" OR "Nigeriens" OR "Nigerian" OR "Nigerians" OR "Northern Mariana Islander" OR "Northern Mariana Islanders" OR "Mariana" OR "Marianas" OR "Omani" OR "Omanis" OR "Pakistani" OR "Pakistanis" OR "Palauan" OR "Palauans" OR "Palestinian" OR "Palestinians" OR "Panamanian" OR "Panamanians" OR "Papua New Guinean" OR "Papua New Guineans" OR "Paraguayan" OR "Paraguayans" OR "Peruvian" OR "Peruvians" OR "Philippine" OR "Philippines" OR "Philipine" OR "Philipines" OR "Phillipine" OR "Phillipines" OR "Phillippine" OR "Phillippines" OR "Filipino" OR "Filipinos" OR "Filipina" OR "Filipinas" OR "Portuguese" OR "Puerto Rican" OR "Puerto Ricans" OR "Romanian" OR "Romanians" OR "Rwandan" OR "Rwandans" OR "Rwandese" OR "Ruandan" OR "Ruandans" OR "Ruandese" OR "Samoan" OR "Samoans" OR "Sao Tomean" OR "Sao Tomeans" OR "Santomean" OR "Santomeans" OR "Senegalese" OR "Montenegrin" OR "Montenegrins" OR "Seychellois" OR "Seychelloise" OR "Seychelloises" OR "Sierra Leonean" OR "Sierra Leoneans" OR "Solomon Islander" OR "Solomon Islanders" OR "Somali" OR "Somalis" OR "South African" OR "South Africans" OR "South Sudanese" OR "Sri Lankan" OR "Sri Lankans" OR "Ceylonese" OR "Kittitian" OR "Kittitians" OR "Nevisian" OR "Nevisians" OR "Saint Lucian" OR "Saint Lucians" OR "Vincentian" OR "Vincentians" OR "Sudanese" OR "Surinamese" OR "Surinameses" OR "Syrian" OR "Syrians" OR "Tajik" OR "Tajiks" OR "Tajikistani" OR "Tajikistanis" OR "Tanzanian" OR "Tanzanians" OR "Tanganyikan" OR "Tanganyikans" OR "Thai" OR "Timorese" OR "Timoreses" OR "Togolese" OR "Tongan" OR "Tongans" OR "Trinidadian" OR "Trinidadians" OR "Tobagonian" OR "Tobagonians" OR "Tunisian" OR "Tunisians" OR "Turkmen" OR "Turkmens" OR "Tuvaluan" OR "Tuvaluans" OR "Ugandan" OR "Ugandans" OR "Ukrainian" OR "Ukrainians" OR "Uruguayan" OR "Uruguayans" OR "Uzbek" OR "Uzbeks" OR "Vanuatu" OR "Vanuatuan" OR "Vanuatuans" OR "Venezuelan" OR "Venezuelans" OR "Vietnamese" OR "Yemeni" OR "Yemenis" OR "Yemenite" OR "Yemenites" OR "Yemenese" OR "Yugoslav" OR "Yugoslavs" OR "Yugoslavian" OR "Yugoslavians" OR "Zambian" OR "Zambians" OR "Zimbabwean" OR "Zimbabweans")**)**

NOT ((MH "Animals+") NOT (MH "Human"))

NOT (SO "plant disease")

Limits: January 2000 – December 2021

**Web of Science Core Collection; Web of Science**

|  | **Concept: trauma centers** | **Concept: injuries** |
| --- | --- | --- |
| Topic  (searches title, abstract, author keywords, Keywords Plus®) | "Emergency Medical Service*"  "Emergency Service*"  "emergency center*"  "emergency centre*"  "emergency health care service*"  "emergency healthcare service*"  "trauma centre*"  "trauma center*"  "trauma system"  "trauma systems"  "trauma care"  "casualty care"  "acute care"  "acute medical care"  "emergency health care"  "emergency medical care"  Emergicent*  **(**(prehospital OR "pre-hospital") AND (intervention* OR procedure* OR treatment* OR "care" OR management OR aid OR program* OR training*)**)** | trauma  wound*  injur*  fractur*  lacerat*  rupture*  perforat*  avulsion*  contusion*  barotrauma*  burn*  concussion*  hemorrhage*  hematoma* |

|  | **Concept: explosive ordnance** | **Concept: injuries** | **Concept: Medical management** |
| --- | --- | --- | --- |
| Topic  (searches title, abstract, author keywords, Keywords Plus®) | APM  blast  bomb*  detonation*  EED  ERW  explosion*  explosive*  IED  grenade*  landmine*  "mine"  "mines"  missile*  mortar*  ordnance  rocket*  UXB  UXO | trauma  wound*  injur*  fractur*  lacerat*  rupture*  perforat*  avulsion*  contusion*  barotrauma*  burn*  concussion*  hemorrhage*  hematoma* | intervention*  procedure*  treatment*  "care"  management  aid  program*  training* |

*(To run, go to the advanced search page and paste everything between the ----- lines into the Query Preview box.)*

-----

**TS=(**

**((**(("Emergency Medical Service*" OR "Emergency Service*" OR "emergency center*" OR "emergency centre*" OR "emergency health care service*" OR "emergency healthcare service*" OR "trauma centre*" OR "trauma center*" OR "trauma system" OR "trauma systems" OR "trauma care" OR "acute care" OR "acute medical care" OR "emergency health care" OR "emergency medical care" OR ((prehospital OR "pre-hospital") NEAR/3 (intervention* OR procedure* OR treatment* OR "care" OR management OR aid OR program* OR training*))) **AND** ("trauma" OR wound* OR injur* OR fractur* OR lacerat* OR rupture* OR perforat* OR avulsion* OR contusion* OR barotrauma* OR burn* OR concussion* OR hemorrhage* OR hematoma*))**)**

**OR**

**(**(APM OR blast OR bomb* OR detonation* OR EED OR ERW OR explosion* OR explosive* OR grenade* OR IED OR landmine* OR "mine" OR "mines" OR missile* OR mortar* OR ordnance OR rocket* OR UXB OR UXO) **AND** (("trauma" OR wound* OR injur* OR fractur* OR lacerat* OR rupture* OR perforat* OR avulsion* OR contusion* OR barotrauma* OR burn* OR concussion* OR hemorrhage* OR hematoma*) NEAR/3 (intervention* OR procedure* OR treatment* OR "care" OR management OR aid OR program* OR training*))**))**

**AND**

**(**(Afghanistan OR Albania OR Algeria OR "American Samoa" OR Angola OR Antigua OR Barbuda OR Argentina OR 'Argentine Republic' OR Armenia OR Armenian OR Aruba OR Azerbaijan OR Bahrain OR Bangladesh OR Barbados OR Belarus OR Byelarus OR Belorussia OR Byelorussian OR Belize OR "British Honduras" OR Benin OR Dahomey OR Bhutan OR Bolivia OR Bosnia OR Hercegovina OR Herzegovina OR Botswana OR Bechuanaland OR Brazil OR Brasil OR Bulgaria OR "Burkina Faso" OR "Burkina Fasso" OR "Upper Volta" OR Burundi OR Urundi OR "Cabo Verde" OR "Cape Verde" OR Cambodia OR Kampuchea OR "Khmer Republic" OR Cameroon OR Cameron OR Cameroun OR "Central African Republic" OR "Ubangi Shari" OR Chad OR Chile OR Colombia OR Comoros OR Comores OR "Comoro Islands" OR Mayotte OR Congo OR Zaire OR "Costa Rica" OR "Cote D’ivoire" OR "Cote D’ Ivoire" OR "Cote Divoire" OR "Cote D Ivoire" OR "Ivory Coast" OR Croatia OR Cuba OR Cyprus OR "Czech Republic" OR Czechoslovakia OR Djibouti OR "French Somaliland" OR Dominica OR "Dominican Republic" OR Ecuador OR Egypt OR "United Arab Republic" OR "El Salvador" OR "Equatorial Guinea" OR "Spanish Guinea" OR Eritrea OR Estonia OR Eswatini OR Swaziland OR Ethiopia OR Fiji OR Gabon OR "Gabonese Republic" OR Gambia OR Georgia OR Georgian OR Ghana OR "Gold Coast" OR Gibraltar OR Greece OR Grenada OR Guam OR Guatemala OR Guinea OR Guyana OR Guiana OR Haiti OR Hispaniola OR Honduras OR India OR Indonesia OR Timor OR Iran OR Iraq OR "Isle Of Man" OR Jamaica OR Jordan OR Kazakhstan OR Kazakh OR Kenya OR Korea OR Kosovo OR Kyrgyzstan OR Kirghizia OR Kirgizstan OR "Kyrgyz Republic" OR Kirghiz OR Laos OR "Lao Pdr" OR "Lao People's Democratic Republic" OR Latvia OR Lebanon OR Lesotho OR Basutoland OR Liberia OR Libya OR "Libyan Arab Jamahiriya" OR Lithuania OR Macau OR Macao OR Macedonia OR Madagascar OR "Malagasy Republic" OR Malawi OR Nyasaland OR Malay OR Malaya OR Malaysia OR Maldives OR "Indian Ocean" OR Mali OR Malta OR Micronesia OR Kiribati OR "Marshall Islands" OR Nauru OR "Northern Mariana Islands" OR Palau OR Tuvalu OR Mauritania OR Mauritius OR Mexico OR Moldova OR Moldovian OR Mongolia OR Montenegro OR Morocco OR Ifni OR Mozambique OR "Portuguese East Africa" OR Myanmar OR Burma OR Namibia OR Nepal OR "Netherlands Antilles" OR Nicaragua OR Niger OR Nigeria OR Oman OR Muscat OR Pakistan OR Panama OR "Papua New Guinea" OR Paraguay OR Peru OR Philippines OR Philipines OR Phillipines OR Phillippines OR Portugal OR "Portuguese Republic" OR "Puerto Rico" OR Romania OR Rwanda OR Ruanda OR Samoa OR "Pacific Islands" OR Polynesia OR "Samoan Islands" OR "Sao Tome" OR Principe OR Senegal OR Seychelles OR "Sierra Leone" OR Melanesia OR "Solomon Island" OR "Solomon Islands" OR "Norfolk Island" OR Somalia OR "South Africa" OR "South Sudan" OR "Sri Lanka" OR Ceylon OR "Saint Kitts And Nevis" OR "St Kitts And Nevis" OR "Saint Lucia" OR "St Lucia" OR "Saint Vincent" OR "St Vincent" OR Grenadines OR Sudan OR Suriname OR Surinam OR Syria OR "Syrian Arab Republic" OR Tajikistan OR Tadjikistan OR Tadzhikistan OR Tadzhik OR Tanzania OR Tanganyika OR Thailand OR Siam OR "Timor Leste" OR "East Timor" OR Togo OR "Togolese Republic" OR Tonga OR Trinidad OR Tobago OR Tunisia OR Turkmenistan OR Turkmen OR Uganda OR Ukraine OR Uruguay OR Uzbekistan OR Uzbek OR Vanuatu OR "New Hebrides" OR Venezuela OR Vietnam OR "Viet Nam" OR "Middle East" OR "West Bank" OR Gaza OR Palestine OR Yemen OR Yugoslavia OR Zambia OR Zimbabwe OR "Northern Rhodesia")

OR

("global south" OR Africa* OR Magreb OR Maghreb OR Sahara OR "West Indies" OR "Indian Ocean islands" OR Caribbean OR "Central America*" OR "Latin America*" OR "South America*" OR "Central Asia*" OR "north Asia*" OR "northern Asia*" OR "southeastern Asia*" OR "south eastern Asia*" OR "southeast Asia*" OR "south east Asia*" OR "western Asia*" OR "Middle East*" OR "east Europe*" OR "eastern Europe*")

OR

("developing country" OR "developing countries" OR "developing nation" OR "developing nations" OR "developing population" OR "developing populations" OR "developing world" OR "less developed country" OR "less developed countries" OR "less developed nation" OR "less developed nations" OR "less developed world" OR "lesser developed countries" OR "lesser developed nations" OR "under developed country" OR "under developed countries" OR "under developed nations" OR "under developed world" OR "underdeveloped country" OR "underdeveloped countries" OR "underdeveloped nation" OR "underdeveloped nations" OR "underdeveloped population" OR "underdeveloped populations" OR "underdeveloped world" OR "middle income country" OR "middle income countries" OR "middle income nation" OR "middle income nations" OR "middle income population" OR "middle income populations" OR "low income country" OR "low income countries" OR "low income nation" OR "low income nations" OR "low income population" OR "low income populations" OR "lower income country" OR "lower income countries" OR "lower income nations" OR "lower income population" OR "lower income populations" OR "underserved countries" OR "underserved nations" OR "underserved population" OR "underserved populations" OR "under served population" OR "under served populations" OR "deprived countries" OR "deprived population" OR "deprived populations" OR "poor country" OR "poor countries" OR "poor nation" OR "poor nations" OR "poor population" OR "poor populations" OR "poor world" OR "poorer countries" OR "poorer nations" OR "poorer population" OR "poorer populations" OR "developing economy" OR "developing economies" OR "less developed economy" OR "less developed economies" OR "underdeveloped economies" OR "middle income economy" OR "middle income economies" OR "low income economy" OR "low income economies" OR "lower income economies" OR "low gdp" OR "low gnp" OR "low gross domestic" OR "low gross national" OR "lower gdp" OR "lower gross domestic" OR lmic OR lmics OR "third world" OR "lami country" OR "lami countries" OR "transitional country" OR "transitional countries" OR "emerging economies" OR "emerging nation" OR "emerging nations" OR "low resource" OR austere)

OR

(Afghan OR Afghans OR Afghani OR Albanian OR Albanians OR Algerian OR Algerians OR "American Samoan" OR "American Samoans" OR Angolan OR Angolans OR Antiguan OR Antiguans OR Barbudan OR Berbudans OR Argentine OR Argentines OR Argentinian OR Argentinians OR Argentinean OR Argentineans OR Armenian OR Armenians OR Aruban OR Arubans OR Azerbaijani OR Azerbaijanis OR Bahraini OR Bahrainis OR Bangladeshi OR Bangladeshis OR Bangalees OR Bajan OR Bajans OR Barbadian OR Barbadians OR Belarusian OR Belarusians OR Byelorussian OR Byelorussians OR Belizean OR Belizeans OR Beninese OR Benineses OR Bhutanese OR Bolivian OR Bolivians OR Bosnian OR Bosnians OR Botswana OR Batswana OR Brazilian OR Brazilians OR Brasilian OR Brasilians OR Bulgarian OR Bulgarians OR Burkinabe OR Burkinese OR Burundian OR Burundians OR "Cape Verdean" OR "Cape Verdeans" OR "Cabo Verdean" OR "Cabo Verdeans" OR Cambodian OR Cambodians OR Khmer OR Cameroonian OR Cameroonians OR "Central African" OR "Central Africans" OR Chadian OR Chadians OR Chilean OR Chileans OR Colombian OR Colombians OR Comorian OR Comorians OR Congolese OR "Costa Rican" OR "Costa Ricans" OR Ivorian OR Ivorians OR Croatian OR Croatians OR Cuban OR Cubans OR Cypriot OR Cypriots OR Czech OR Czechs OR Djiboutian OR Djiboutians OR Dominican OR Dominicans OR Ecuadorian OR Ecuadorians OR Egyptian OR Egyptians OR Salvadoran OR Salvadorans OR "Equatorial Guinean" OR "Equatorial Guineans" OR Equatoguinean OR Equatoguineans OR Eritrean OR Eritreans OR Estonian OR Estonians OR Swazi OR Swazis OR Swati OR Swatis OR Ethiopian OR Ethiopians OR Fijian OR Fijians OR Gabonese OR Gabonaise OR Gambian OR Gambians OR Georgian OR Georgians OR Ghanaian OR Ghanaians OR Gibraltarian OR Gibraltarians OR Greek OR Greeks OR Grenadian OR Grenadians OR Guamanian OR Guamanians OR Guatemalan OR Guatemalans OR Guinean OR Guineans OR "Bissau Guinean" OR "Bissau Guineans" OR Guyanese OR Haitian OR Haitians OR Honduran OR Hondurans OR Indian OR Indians OR Indonesian OR Indonesians OR Iranian OR Iranians OR Iraqian OR Iraqians OR Iraqi OR Iraqis OR Manx OR Jamaican OR Jamaicans OR Jordanian OR Jordanians OR Kazakhstani OR Kazakhstanis OR Kenyan OR Kenyans OR Kirabati OR Kirabatian OR Kirabatians OR Korean OR Koreans OR Kosovar OR Kosovars OR Kosovan OR Kosovans OR Kyrgyzstani OR Kyrgyzstanis OR Kyrgyz OR Lao OR Laotian OR Laotians OR Latvian OR Latvians OR Lebanese OR Lesothan OR Lesothans OR Lesothonian OR Lesothonians OR Mosotho OR Basotho OR Liberian OR Liberians OR Libyan OR Libyans OR Lithuanian OR Lithuanians OR Macanese OR Macedonian OR Macedonians OR Malagasy OR Madagascan OR Madagascans OR Malawian OR Malawians OR Malay OR Malaya OR Malaysian OR Malaysians OR Maldivian OR Maldivians OR Malian OR Malians OR Maltese OR Marshallese OR Marshalleses OR Mauritanian OR Mauritanians OR Mauritian OR Mauritians OR Mexican OR Mexicans OR Micronesian OR Micronesians OR Moldovan OR Moldovans OR Mongolian OR Mongolians OR Mongol OR Montenegrin OR Montenegrins OR Moroccan OR Moroccans OR Mozambican OR Mozambicans OR Burmese OR Myanma OR Namibian OR Namibians OR Nauruan OR Nauruans OR Nepali OR Nepalese OR "Netherlands Antillean" OR "Netherlands Antilleans" OR Nicaraguan OR Nicaraguans OR Nigerien OR Nigeriens OR Nigerian OR Nigerians OR "Northern Mariana Islander" OR "Northern Mariana Islanders" OR Mariana OR Marianas OR Omani OR Omanis OR Pakistani OR Pakistanis OR Palauan OR Palauans OR Palestinian OR Palestinians OR Panamanian OR Panamanians OR "Papua New Guinean" OR "Papua New Guineans" OR Paraguayan OR Paraguayans OR Peruvian OR Peruvians OR Philippine OR Philippines OR Philipine OR Philipines OR Phillipine OR Phillipines OR Phillippine OR Phillippines OR Filipino OR Filipinos OR Filipina OR Filipinas OR Portuguese OR "Puerto Rican" OR "Puerto Ricans" OR Romanian OR Romanians OR Rwandan OR Rwandans OR Rwandese OR Ruandan OR Ruandans OR Ruandese OR Samoan OR Samoans OR "Sao Tomean" OR "Sao Tomeans" OR Santomean OR Santomeans OR Senegalese OR Montenegrin OR Montenegrins OR Seychellois OR Seychelloise OR Seychelloises OR "Sierra Leonean" OR "Sierra Leoneans" OR "Solomon Islander" OR "Solomon Islanders" OR Somali OR Somalis OR "South African" OR "South Africans" OR "South Sudanese" OR "Sri Lankan" OR "Sri Lankans" OR Ceylonese OR Kittitian OR Kittitians OR Nevisian OR Nevisians OR "Saint Lucian" OR "Saint Lucians" OR Vincentian OR Vincentians OR Sudanese OR Surinamese OR Surinameses OR Syrian OR Syrians OR Tajik OR Tajiks OR Tajikistani OR Tajikistanis OR Tanzanian OR Tanzanians OR Tanganyikan OR Tanganyikans OR Thai OR Timorese OR Timoreses OR Togolese OR Tongan OR Tongans OR Trinidadian OR Trinidadians OR Tobagonian OR Tobagonians OR Tunisian OR Tunisians OR Turkmen OR Turkmens OR Tuvaluan OR Tuvaluans OR Ugandan OR Ugandans OR Ukrainian OR Ukrainians OR Uruguayan OR Uruguayans OR Uzbek OR Uzbeks OR Vanuatu OR Vanuatuan OR Vanuatuans OR Venezuelan OR Venezuelans OR Vietnamese OR Yemeni OR Yemenis OR Yemenite OR Yemenites OR Yemenese OR Yugoslav OR Yugoslavs OR Yugoslavian OR Yugoslavians OR Zambian OR Zambians OR Zimbabwean OR Zimbabweans)**)**

**)**

AND PY=(2000-2021)

**Global Index Medicus**

(includes African Index Medicus (AIM), Index Medicus for the Eastern Mediterranean Region (IMEMR), Index Medicus for the South-East Asia Region (IMSEAR), Latin America and the Caribbean Literature on Health Sciences (LILACS), and Western Pacific Region Index Medicus (WPRO))

|  | **Concept: trauma centers** | **Concept: injuries** |
| --- | --- | --- |
| Subject Descriptors  (MH:()) | N02.421.297*  N02.278.216.500.968.336*  MH:"trauma centers" | "Wounds and Injuries"  C26.017*  C26.040*  C26.062*  C26.088*  C23.550.260.095*  C26.117*  C26.120*  C16.614.131*  C26.200*  C26.212*  "Contrecoup Injury"  C26.257*  C23.550.260.393*  C26.324*  "Esophageal Perforation"  C26.392*  C26.404*  "Fractures, Cartilage"  C26.212.500*  C26.448*  C26.522*  C26.531*  C05.550.518*  "Lacerations"  C26.558*  "Microtrauma, Physical"  C26.640*  C08.460.595*  C26.700*  "Occupational Injuries"  C26.733*  "Reinjuries"  "Retropneumoperitoneum"  C26.761*  "Self Mutilation"  C23.550.835.888*  C26.803*  C26.808*  C10.228.854.763*  C26.844*  C26.859*  C26.874*  C26.891*  C07.793.850*  C10.900*  "Tympanic Membrane Perforation"  "Vascular System Injuries"  "War-Related Injuries"  C26.974*  C26.986* |
| Title, abstract, subject  (tw:()) | "Emergency Medical Service"  "Emergency Medical Services"  "Emergency Service"  "Emergency Services"  "emergency center"  "emergency centers"  "emergency centre"  "emergency centres"  "emergency health care service"  "emergency health care services"  "emergency healthcare service"  "emergency healthcare services"  "trauma centre"  "trauma centres"  "trauma center"  "trauma centers"  "trauma system"  "trauma systems"  "trauma care"  "casualty care"  "acute care"  "acute medical care"  "emergency health care"  "emergency medical care"  Emergicenter  Emergicenters  **(**(prehospital OR "pre-hospital")  AND  (intervention* OR procedure* OR treatment* OR "care" OR management OR aid OR program* OR training*)**)** | trauma  wound*  injur*  fractur*  lacerat*  rupture*  perforat*  avulsion*  contusion*  barotrauma*  burn*  concussion*  hemorrhage*  hematoma* |

|  | **Concept: explosive ordnance** | **Concept: injuries** | **Concept: medical management** |
| --- | --- | --- | --- |
| Subject Descriptors  (MH:()) | "Explosive agents"  J01.637.870.175*  "Blast Injuries"  "Explosions" | "Wounds and Injuries"  C26.017*  C26.040*  C26.062*  C26.088*  C23.550.260.095*  C26.117*  C26.120*  C16.614.131*  C26.200*  C26.212*  "Contrecoup Injury"  C26.257*  C23.550.260.393*  C26.324*  "Esophageal Perforation"  C26.392*  C26.404*  "Fractures, Cartilage"  C26.212.500*  C26.448*  C26.522*  C26.531*  C05.550.518*  "Lacerations"  C26.558*  "Microtrauma, Physical"  C26.640*  C08.460.595*  C26.700*  "Occupational Injuries"  C26.733*  "Reinjuries"  "Retropneumoperitoneum"  C26.761*  "Self Mutilation"  C23.550.835.888*  C26.803*  C26.808*  C10.228.854.763*  C26.844*  C26.859*  C26.874*  C26.891*  C07.793.850*  C10.900*  "Tympanic Membrane Perforation"  "Vascular System Injuries"  "War-Related Injuries"  C26.974*  C26.986* |  |
| Title, abstract, subject  (tw:()) | APM  Blast  bomb  bombs  bombing*  bombed  detonation*  EED  ERW  explosion*  explosive*  IED  grenade*  landmine*  "mine"  "mines"  missile*  mortar*  ordnance  rocket*  UXB  UXO | trauma  wound*  injur*  fractur*  lacerat*  rupture*  perforat*  avulsion*  contusion*  barotrauma*  burn*  concussion*  hemorrhage*  hematoma* | intervention*  procedure*  treatment*  "care"  management  aid  program*  training* |

**((**(MH: (N02.421.297* OR N02.278.216.500.968.336* OR "trauma centers") OR TW:( "Emergency Medical Service" OR "Emergency Medical Services" OR "Emergency Service" OR "Emergency Services" OR "emergency center" OR "emergency centers" OR "emergency centre" OR "emergency centres" OR "emergency health care service" OR "emergency health care services" OR "emergency healthcare service" OR "emergency healthcare services" OR "trauma centre" OR "trauma centres" OR "trauma center" OR "trauma centers" OR "trauma system" OR "trauma systems" OR "trauma care" OR "casualty care" OR "acute care" OR "acute medical care" OR "emergency health care" OR "emergency medical care" OR Emergicenter OR Emergicenters) OR **(**(prehospital OR "pre-hospital") AND (intervention* OR procedure* OR treatment* OR "care" OR management OR aid OR program* OR training*)**)**) **AND** (MH:("Wounds and Injuries" OR C26.017* OR C26.040* OR C26.062* OR C26.088* OR C23.550.260.095* OR C26.117* OR C26.120* OR C16.614.131* OR C26.200* OR C26.212* OR "Contrecoup Injury" OR C26.257* OR C23.550.260.393* OR C26.324* OR "Esophageal Perforation" OR C26.392* OR C26.404* OR "Fractures, Cartilage" OR C26.212.500* OR C26.448* OR C26.522* OR C26.531* OR C05.550.518* OR "Lacerations" OR C26.558* OR "Microtrauma, Physical" OR C26.640* OR C08.460.595* OR C26.700* OR "Occupational Injuries" OR C26.733* OR "Reinjuries" OR "Retropneumoperitoneum" OR C26.761* OR "Self Mutilation" OR C23.550.835.888* OR C26.803* OR C26.808* OR C10.228.854.763* OR C26.844* OR C26.859* OR C26.874* OR C26.891* OR C07.793.850* OR C10.900* OR "Tympanic Membrane Perforation" OR "Vascular System Injuries" OR "War-Related Injuries" OR C26.974* OR C26.986*) OR TW:(trauma OR wound* OR injur* OR fractur* OR lacerat* OR rupture* OR perforat* OR avulsion* OR contusion* OR barotrauma* OR burn* OR concussion* OR hemorrhage* OR hematoma*))**)**

**OR**

**(**(MH:("Explosive agents" OR J01.637.870.175* OR "Blast Injuries" OR "Explosions") OR TW:( APM OR blast OR bomb OR bombs OR bombing* OR bombed OR detonation* OR EED OR ERW OR explosion* OR explosive* OR IED OR grenade* OR landmine* OR "mine" OR "mines" OR missile* OR mortar* OR ordnance OR rocket* OR UXB OR UXO) ) **AND** (MH:("Wounds and Injuries" OR C26.017* OR C26.040* OR C26.062* OR C26.088* OR C23.550.260.095* OR C26.117* OR C26.120* OR C16.614.131* OR C26.200* OR C26.212* OR "Contrecoup Injury" OR C26.257* OR C23.550.260.393* OR C26.324* OR "Esophageal Perforation" OR C26.392* OR C26.404* OR "Fractures, Cartilage" OR C26.212.500* OR C26.448* OR C26.522* OR C26.531* OR C05.550.518* OR "Lacerations" OR C26.558* OR "Microtrauma, Physical" OR C26.640* OR C08.460.595* OR C26.700* OR "Occupational Injuries" OR C26.733* OR "Reinjuries" OR "Retropneumoperitoneum" OR C26.761* OR "Self Mutilation" OR C23.550.835.888* OR C26.803* OR C26.808* OR C10.228.854.763* OR C26.844* OR C26.859* OR C26.874* OR C26.891* OR C07.793.850* OR C10.900* OR "Tympanic Membrane Perforation" OR "Vascular System Injuries" OR "War-Related Injuries" OR C26.974* OR C26.986*) OR TW:(trauma OR wound* OR injur* OR fractur* OR lacerat* OR rupture* OR perforat* OR avulsion* OR contusion* OR barotrauma* OR burn* OR concussion* OR hemorrhage* OR hematoma*)) **AND** (TW:(intervention* OR procedure* OR treatment* OR "care" OR management OR aid OR program* OR training*))**))**

AND (year_cluster:[2000 TO 2021])

**Global Health; EBSCO**

|  | **Concept: trauma centers** | **Concept: injuries** |
| --- | --- | --- |
| Descriptors  DE:() | ((DE "emergencies") AND (DE "health services" OR DE "health centres")) | "trauma"  "injuries"  "abrasion"  "amputation"  "asphyxia"  "bone fractures"  "bruising"  "burns"  "cold injury"  "dislocations"  "fractures"  "frostbite"  "heat injury"  "heat stress"  "radiation injuries"  "rupture"  "shock"  "wounds" |
| Searched in Title & Abstract | "Emergency Medical Service*"  "Emergency Service*"  "emergency center*"  "emergency centre*"  "emergency health care service*"  "emergency healthcare service*"  "trauma centre*"  "trauma center*"  "trauma system"  "trauma systems"  "trauma care"  "casualty care"  "acute care"  "acute medical care"  "emergency health care"  "emergency medical care"  Emergicent*  **(**(prehospital OR "pre-hospital") N2 (intervention* OR procedure* OR treatment* OR "care" OR management OR aid OR program* OR training*)**)** | trauma  wound*  injur*  fractur*  lacerat*  rupture*  perforat*  avulsion*  contusion*  barotrauma*  burn*  concussion*  hemorrhage*  hematoma* |

|  | **Concept: explosive ordnance** | **Concept: injuries** | **Concept: Medical management** |
| --- | --- | --- | --- |
| Descriptors  DE:() | DE "explosive hazard"  DE "explosives"  DE "explosions" | "trauma"  "injuries"  "abrasion"  "amputation"  "asphyxia"  "bone fractures"  "bruising"  "burns"  "cold injury"  "dislocations"  "fractures"  "frostbite"  "heat injury"  "heat stress"  "radiation injuries"  "rupture"  "shock"  "wounds" | DE "intervention"  DE "medical treatment"  DE "wound treatment" |
| Searched in Title & Abstract | APM  blast  bomb*  detonation*  EED  ERW  explosion*  explosive*  IED  grenade*  landmine*  "mine"  "mines"  missile*  mortar*  ordnance  rocket*  UXB  UXO | trauma  wound*  injur*  fractur*  lacerat*  rupture*  perforat*  avulsion*  contusion*  barotrauma*  burn*  concussion*  hemorrhage*  hematoma* | intervention*  procedure*  treatment*  "care"  management  aid  program*  training* |

**((**(((DE "emergencies") AND (DE "health services" OR DE "health centres")) OR TI ("Emergency Medical Service*" OR "Emergency Service*" OR "emergency center*" OR "emergency centre*" OR "emergency health care service*" OR "emergency healthcare service*" OR "trauma centre*" OR "trauma center*" OR "trauma system" OR "trauma systems" OR "trauma care" OR "casualty care" OR "acute care" OR "acute medical care" OR "emergency health care" OR "emergency medical care" OR Emergicent* OR **(**(prehospital OR "pre-hospital") N2 (intervention* OR procedure* OR treatment* OR "care" OR management OR aid OR program* OR training*)**)**) OR AB ("Emergency Medical Service*" OR "Emergency Service*" OR "emergency center*" OR "emergency centre*" OR "emergency health care service*" OR "emergency healthcare service*" OR "trauma centre*" OR "trauma center*" OR "trauma system" OR "trauma systems" OR "trauma care" OR "casualty care" OR "acute care" OR "acute medical care" OR "emergency health care" OR "emergency medical care" OR Emergicent* OR **(**(prehospital OR "pre-hospital") N2 (intervention* OR procedure* OR treatment* OR "care" OR management OR aid OR program* OR training*)**)**) OR ((DE ("intervention" OR "medical treatment" OR "wound treatment") AND (TI (prehospital OR "pre-hospital) OR AB (prehospital OR "pre-hospital)))) **AND** (DE ("trauma" OR "injuries" OR "abrasion" OR "amputation" OR "asphyxia" OR "bone fractures" OR "bruising" OR "burns" OR "cold injury" OR "dislocations" OR "fractures" OR "frostbite" OR "heat injury" OR "heat stress" OR "radiation injuries" OR "rupture" OR "shock" OR "wounds") OR TI (trauma OR wound* OR injur* OR fractur* OR lacerat* OR rupture* OR perforat* OR avulsion* OR contusion* OR barotrauma* OR burn* OR concussion* OR hemorrhage* OR hematoma*) OR AB (trauma OR wound* OR injur* OR fractur* OR lacerat* OR rupture* OR perforat* OR avulsion* OR contusion* OR barotrauma* OR burn* OR concussion* OR hemorrhage* OR hematoma*))**)**

**OR**

**(**(DE ("explosive hazard" OR "explosives" OR "explosions") OR TI (APM OR blast OR bomb* OR detonation* OR EED OR ERW OR explosion* OR explosive* OR IED OR grenade* OR landmine* OR "mine" OR "mines" OR missile* OR mortar* OR ordnance OR rocket* OR UXB OR UXO) OR AB (APM OR blast OR bomb* OR detonation* OR EED OR ERW OR explosion* OR explosive* OR IED OR grenade* OR landmine* OR "mine" OR "mines" OR missile* OR mortar* OR ordnance OR rocket* OR UXB OR UXO)) **AND** (DE ("trauma" OR "injuries" OR "abrasion" OR "amputation" OR "asphyxia" OR "bone fractures" OR "bruising" OR "burns" OR "cold injury" OR "dislocations" OR "fractures" OR "frostbite" OR "heat injury" OR "heat stress" OR "radiation injuries" OR "rupture" OR "shock" OR "wounds") OR TI (trauma OR wound* OR injur* OR fractur* OR lacerat* OR rupture* OR perforat* OR avulsion* OR contusion* OR barotrauma* OR burn* OR concussion* OR hemorrhage* OR hematoma*) OR AB (trauma OR wound* OR injur* OR fractur* OR lacerat* OR rupture* OR perforat* OR avulsion* OR contusion* OR barotrauma* OR burn* OR concussion* OR hemorrhage* OR hematoma*)) **AND** (DE ("intervention" OR "medical treatment" OR "wound treatment") OR TI (intervention* OR procedure* OR treatment* OR "care" OR management OR aid OR program* OR training*) OR AB (intervention* OR procedure* OR treatment* OR "care" OR management OR aid OR program* OR training*))**))**

**AND**

**(**DE ("Afghanistan" OR "Albania" OR "Algeria" OR "American Samoa" OR "Angola" OR "Antigua and Barbuda" OR "Antigua" OR "Barbuda" OR "Argentina" OR "Armenia" OR "Aruba" OR "Azerbaijan" OR "Bahrain" OR "Bangladesh" OR "Barbados" OR "Belarus" OR "Belize" OR "Benin" OR "Bhutan" OR "Bolivia" OR "Bosnia-Hercegovina" OR "Botswana" OR "Brazil" OR "Bulgaria" OR "Burkina Faso" OR "Burundi" OR "Cape Verde" OR "Cambodia" OR "Cameroon" OR "Central African Republic" OR "Chad" OR "Chile" OR "Colombia" OR "Comoros" OR "Congo Democratic Republic" OR "Congo" OR "Costa Rica" OR "Cote d'Ivoire" OR "Croatia" OR "Cuba" OR "Cyprus" OR "Czech Republic" OR "Czechoslovakia" OR "Djibouti" OR "Dominica" OR "Dominican Republic" OR "Ecuador" OR "Egypt" OR "El Salvador" OR "Equatorial Guinea" OR "Eritrea" OR "Estonia" OR "Swaziland" OR "Ethiopia" OR "Fiji" OR "Gabon" OR "Gambia" OR "Republic of Georgia" OR "Ghana" OR "Gibraltar" OR "Greece" OR "Grenada" OR "Guam" OR "Guatemala" OR "Guinea" OR "Guinea-Bissau" OR "Guyana" OR "Haiti" OR "Honduras" OR "India" OR "Indochina" OR "Indonesia" OR "Iran" OR "Iraq" OR "Jamaica" OR "Jordan" OR "Kazakhstan" OR "Kenya" OR "Korea Democratic People's Republic" OR "Korea Republic" OR "Kosovo" OR "Kyrgyzstan" OR "Laos" OR "Latvia" OR "Lebanon" OR "Lesotho" OR "Liberia" OR "Libya" OR "Lithuania" OR "Macao" OR "Republic of Macedonia" OR "Madagascar" OR "Malawi" OR "Malaysia" OR "Mali" OR "Malta" OR "Melanesia" OR "Micronesia" OR "Palau" OR "Mauritania" OR "Mauritius" OR "Mexico" OR "Moldova" OR "Mongolia" OR "Montenegro" OR "Morocco" OR "Mozambique" OR "Myanmar" OR "Namibia" OR "Nepal" OR "Netherlands Antilles" OR "Nicaragua" OR "Niger" OR "Nigeria" OR "Oman" OR "Pakistan" OR "Palestine" OR "Panama" OR "Papua New Guinea" OR "Paraguay" OR "Peru" OR "Philippines" OR "Portugal" OR "Puerto Rico" OR "Romania" OR "Rwanda" OR "Samoa" OR "Sao Tome and Principe" OR "Principe" OR "Sao Tome" OR "Senegal" OR "Seychelles" OR "Sierra Leone" OR "Somalia" OR "South Africa" OR "Sri Lanka" OR "Saint Kitts and Nevis" OR "Nevis" OR "Saint Kitts" OR "Saint Lucia" OR "Saint Vincent and the Grenadines" OR "Saint Vincent" OR "Sudan" OR "Suriname" OR "Syria" OR "Tajikistan" OR "Tanzania" OR "Thailand" OR "East Timor" OR "Togo" OR "Tonga" OR "Trinidad and Tobago" OR "Tobago" OR "Trinidad" OR "Tunisia" OR "Turkmenistan" OR "Uganda" OR "Ukraine" OR "Uruguay" OR "Uzbekistan" OR "Vanuatu" OR "Venezuela" OR "Vietnam" OR "Yemen" OR "Yugoslavia" OR "Zambia" OR "Zimbabwe")

OR

TI("Afghanistan" OR "Albania" OR "Algeria" OR "American Samoa" OR "Angola" OR "Antigua" OR "Barbuda" OR "Argentina" OR "Argentine Republic" OR "Armenia" OR "Aruba" OR "Azerbaijan" OR "Bahrain" OR "Bangladesh" OR "Barbados" OR "Belarus" OR "Byelarus" OR "Belorussia" OR "Belize" OR "British Honduras" OR "Benin" OR "Dahomey" OR "Bhutan" OR "Bolivia" OR "Bosnia" OR "Hercegovina" OR "Herzegovina" OR "Botswana" OR "Bechuanaland" OR "Brazil" OR "Brasil" OR "Bulgaria" OR "Burkina Faso" OR "Burkina Fasso" OR "Upper Volta" OR "Burundi" OR "Urundi" OR "Cabo Verde" OR "Cape Verde" OR "Cambodia" OR "Kampuchea" OR "Khmer Republic" OR "Cameroon" OR "Cameron" OR "Cameroun" OR "Central African Republic" OR "Ubangi Shari" OR "Chad" OR "Chile" OR "Colombia" OR "Comoros" OR "Comores" OR "Comoro Islands" OR "Mayotte" OR "Democratic Republic of The Congo" OR "Congo" OR "Zaire" OR "Costa Rica" OR "Cote D'ivoire" OR "Cote D' Ivoire" OR "Cote Divoire" OR "Cote D Ivoire" OR "**Côte d'Ivoire" OR "**Ivory Coast" OR "Croatia" OR "Cuba" OR "Cyprus" OR "Czech Republic" OR "Czechoslovakia" OR "Djibouti" OR "French Somaliland" OR "Dominica" OR "Dominican Republic" OR "Ecuador" OR "Egypt" OR "United Arab Republic" OR "El Salvador" OR "Equatorial Guinea" OR "Spanish Guinea" OR "Eritrea" OR "Estonia" OR "Eswatini" OR "Swaziland" OR "Ethiopia" OR "Fiji" OR "Gabon" OR "Gabonese Republic" OR "Gambia" OR "Georgia (Republic)" OR "Georgia" OR "Ghana" OR "Gold Coast" OR "Gibraltar" OR "Greece" OR "Grenada" OR "Guam" OR "Guatemala" OR "Guinea" OR "Guinea Bissau" OR "Guyana" OR "Guiana" OR "Haiti" OR "Hispaniola" OR "Honduras" OR "India" OR "Indonesia" OR "Timor" OR "Iran" OR "Iraq" OR "Isle Of Man" OR "Jamaica" OR "Jordan" OR "Kazakhstan" OR "Kazakh" OR "Kenya" OR "Democratic People's Republic of Korea" OR "Republic of Korea" OR "Korea" OR "Kosovo" OR "Kyrgyzstan" OR "Kirghizia" OR "Kirgizstan" OR "Kyrgyz Republic" OR "Kirghiz" OR "Laos" OR "Lao Pdr" OR "Lao People's Democratic Republic" OR "Latvia" OR "Lebanon" OR "Lesotho" OR "Basutoland" OR "Liberia" OR "Libya" OR "Libyan Arab Jamahiriya" OR "Lithuania" OR "Macau" OR "Macao" OR "Republic of North Macedonia" OR "Macedonia" OR "Madagascar" OR "Malagasy Republic" OR "Malawi" OR "Nyasaland" OR "Malay" OR "Malaya" OR "Malaysia" OR "Maldives" OR "Indian Ocean Islands" OR "Mali" OR "Malta" OR "Melanesia" OR "Micronesia" OR "Kiribati" OR "Marshall Islands" OR "Nauru" OR "Northern Mariana Islands" OR "Palau" OR "Tuvalu" OR "Mauritania" OR "Mauritius" OR "Mexico" OR "Moldova" OR "Mongolia" OR "Montenegro" OR "Morocco" OR "Ifni" OR "Mozambique" OR "Portuguese East Africa" OR "Myanmar" OR "Burma" OR "Namibia" OR "Nepal" OR "Netherlands Antilles" OR "Nicaragua" OR "Niger" OR "Nigeria" OR "Oman" OR "Muscat" OR "Pakistan" OR "Panama" OR "Papua New Guinea" OR "Paraguay" OR "Peru" OR "Philippines" OR "Philipines" OR "Phillipines" OR "Phillippines" OR "Portugal" OR "Portuguese Republic" OR "Puerto Rico" OR "Romania" OR "Rwanda" OR "Ruanda" OR "Samoa" OR "Pacific Islands" OR "Polynesia" OR "Samoan Islands" OR "Sao Tome And Principe" OR "Senegal" OR "Seychelles" OR "Sierra Leone" OR "Solomon Island" OR "Solomon Islands" OR "Norfolk Island" OR "Somalia" OR "South Africa" OR "South Sudan" OR "Sri Lanka" OR "Ceylon" OR "Saint Kitts And Nevis" OR "St Kitts And Nevis" OR "Saint Lucia" OR "St Lucia" OR "Saint Vincent and The Grenadines" OR "Saint Vincent" OR "St Vincent" OR "Grenadines" OR "Sudan" OR "Suriname" OR "Surinam" OR "Syria" OR "Syrian Arab Republic" OR "Tajikistan" OR "Tadjikistan" OR "Tadzhikistan" OR "Tadzhik" OR "Tanzania" OR "Tanganyika" OR "Thailand" OR "Siam" OR "Timor Leste" OR "East Timor" OR "Togo" OR "Togolese Republic" OR "Tonga" OR "Trinidad and Tobago" OR "Trinidad" OR "Tobago" OR "Tunisia" OR "Turkmenistan" OR "Uganda" OR "Ukraine" OR "Uruguay" OR "Uzbekistan" OR "Uzbek" OR "Vanuatu" OR "New Hebrides" OR "Venezuela" OR "Vietnam" OR "Viet Nam" OR "West Bank" OR "Gaza" OR "Palestine" OR "Yemen" OR "Yugoslavia" OR "Zambia" OR "Zimbabwe" OR "Northern Rhodesia")

OR

AB("Afghanistan" OR "Albania" OR "Algeria" OR "American Samoa" OR "Angola" OR "Antigua" OR "Barbuda" OR "Argentina" OR "Argentine Republic" OR "Armenia" OR "Aruba" OR "Azerbaijan" OR "Bahrain" OR "Bangladesh" OR "Barbados" OR "Belarus" OR "Byelarus" OR "Belorussia" OR "Belize" OR "British Honduras" OR "Benin" OR "Dahomey" OR "Bhutan" OR "Bolivia" OR "Bosnia" OR "Hercegovina" OR "Herzegovina" OR "Botswana" OR "Bechuanaland" OR "Brazil" OR "Brasil" OR "Bulgaria" OR "Burkina Faso" OR "Burkina Fasso" OR "Upper Volta" OR "Burundi" OR "Urundi" OR "Cabo Verde" OR "Cape Verde" OR "Cambodia" OR "Kampuchea" OR "Khmer Republic" OR "Cameroon" OR "Cameron" OR "Cameroun" OR "Central African Republic" OR "Ubangi Shari" OR "Chad" OR "Chile" OR "Colombia" OR "Comoros" OR "Comores" OR "Comoro Islands" OR "Mayotte" OR "Democratic Republic of The Congo" OR "Congo" OR "Zaire" OR "Costa Rica" OR "Cote D'ivoire" OR "Cote D' Ivoire" OR "Cote Divoire" OR "Cote D Ivoire" OR "**Côte d'Ivoire" OR "**Ivory Coast" OR "Croatia" OR "Cuba" OR "Cyprus" OR "Czech Republic" OR "Czechoslovakia" OR "Djibouti" OR "French Somaliland" OR "Dominica" OR "Dominican Republic" OR "Ecuador" OR "Egypt" OR "United Arab Republic" OR "El Salvador" OR "Equatorial Guinea" OR "Spanish Guinea" OR "Eritrea" OR "Estonia" OR "Eswatini" OR "Swaziland" OR "Ethiopia" OR "Fiji" OR "Gabon" OR "Gabonese Republic" OR "Gambia" OR "Georgia (Republic)" OR "Georgia" OR "Ghana" OR "Gold Coast" OR "Gibraltar" OR "Greece" OR "Grenada" OR "Guam" OR "Guatemala" OR "Guinea" OR "Guinea Bissau" OR "Guyana" OR "Guiana" OR "Haiti" OR "Hispaniola" OR "Honduras" OR "India" OR "Indonesia" OR "Timor" OR "Iran" OR "Iraq" OR "Isle Of Man" OR "Jamaica" OR "Jordan" OR "Kazakhstan" OR "Kazakh" OR "Kenya" OR "Democratic People's Republic of Korea" OR "Republic of Korea" OR "Korea" OR "Kosovo" OR "Kyrgyzstan" OR "Kirghizia" OR "Kirgizstan" OR "Kyrgyz Republic" OR "Kirghiz" OR "Laos" OR "Lao Pdr" OR "Lao People's Democratic Republic" OR "Latvia" OR "Lebanon" OR "Lesotho" OR "Basutoland" OR "Liberia" OR "Libya" OR "Libyan Arab Jamahiriya" OR "Lithuania" OR "Macau" OR "Macao" OR "Republic of North Macedonia" OR "Macedonia" OR "Madagascar" OR "Malagasy Republic" OR "Malawi" OR "Nyasaland" OR "Malay" OR "Malaya" OR "Malaysia" OR "Maldives" OR "Indian Ocean Islands" OR "Mali" OR "Malta" OR "Melanesia" OR "Micronesia" OR "Kiribati" OR "Marshall Islands" OR "Nauru" OR "Northern Mariana Islands" OR "Palau" OR "Tuvalu" OR "Mauritania" OR "Mauritius" OR "Mexico" OR "Moldova" OR "Mongolia" OR "Montenegro" OR "Morocco" OR "Ifni" OR "Mozambique" OR "Portuguese East Africa" OR "Myanmar" OR "Burma" OR "Namibia" OR "Nepal" OR "Netherlands Antilles" OR "Nicaragua" OR "Niger" OR "Nigeria" OR "Oman" OR "Muscat" OR "Pakistan" OR "Panama" OR "Papua New Guinea" OR "Paraguay" OR "Peru" OR "Philippines" OR "Philipines" OR "Phillipines" OR "Phillippines" OR "Portugal" OR "Portuguese Republic" OR "Puerto Rico" OR "Romania" OR "Rwanda" OR "Ruanda" OR "Samoa" OR "Pacific Islands" OR "Polynesia" OR "Samoan Islands" OR "Sao Tome And Principe" OR "Senegal" OR "Seychelles" OR "Sierra Leone" OR "Solomon Island" OR "Solomon Islands" OR "Norfolk Island" OR "Somalia" OR "South Africa" OR "South Sudan" OR "Sri Lanka" OR "Ceylon" OR "Saint Kitts And Nevis" OR "St Kitts And Nevis" OR "Saint Lucia" OR "St Lucia" OR "Saint Vincent and The Grenadines" OR "Saint Vincent" OR "St Vincent" OR "Grenadines" OR "Sudan" OR "Suriname" OR "Surinam" OR "Syria" OR "Syrian Arab Republic" OR "Tajikistan" OR "Tadjikistan" OR "Tadzhikistan" OR "Tadzhik" OR "Tanzania" OR "Tanganyika" OR "Thailand" OR "Siam" OR "Timor Leste" OR "East Timor" OR "Togo" OR "Togolese Republic" OR "Tonga" OR "Trinidad and Tobago" OR "Trinidad" OR "Tobago" OR "Tunisia" OR "Turkmenistan" OR "Uganda" OR "Ukraine" OR "Uruguay" OR "Uzbekistan" OR "Uzbek" OR "Vanuatu" OR "New Hebrides" OR "Venezuela" OR "Vietnam" OR "Viet Nam" OR "West Bank" OR "Gaza" OR "Palestine" OR "Yemen" OR "Yugoslavia" OR "Zambia" OR "Zimbabwe" OR "Northern Rhodesia")

OR

DE ("Africa" OR "Africa South of Sahara" OR "Central Africa" OR "North Africa" OR "Southern Africa" OR "East Africa" OR "West Africa" OR "Maghreb" OR "Sahel" OR "Western Sahara" OR "Caribbean" OR "Indian Ocean Islands" OR "Central America" OR "Latin America" OR "South America" OR "Central Asia" OR "South Asia" OR "South East Asia" OR "West Asia" OR "Polynesia" OR "Windward Islands" OR "Middle East" OR "Central Europe")

OR

TI ("global south" OR Africa* OR Magreb OR Maghreb OR Sahara OR Sahel OR "West Indies" OR "Indian Ocean islands" OR Caribbean OR "Central America*" OR "Latin America*" OR "South America*" OR "Central Asia*" OR "north Asia*" OR "northern Asia*" OR "southeastern Asia*" OR "south eastern Asia*" OR "southeast Asia*" OR "south east Asia*" OR "western Asia*" OR "Middle East*" OR "east Europe*" OR "eastern Europe*")

OR

AB ("global south" OR Africa* OR Magreb OR Maghreb OR Sahara OR Sahel OR "West Indies" OR "Indian Ocean islands" OR Caribbean OR "Central America*" OR "Latin America*" OR "South America*" OR "Central Asia*" OR "north Asia*" OR "northern Asia*" OR "southeastern Asia*" OR "south eastern Asia*" OR "southeast Asia*" OR "south east Asia*" OR "western Asia*" OR "Middle East*" OR "east Europe*" OR "eastern Europe*")

OR

DE ("Developing Countries" OR "Least Developed Countries")

OR

TI ("developing country" OR "developing countries" OR "developing nation" OR "developing nations" OR "developing population" OR "developing populations" OR "developing world" OR "less developed country" OR "less developed countries" OR "less developed nation" OR "less developed nations" OR "less developed world" OR "lesser developed countries" OR "lesser developed nations" OR "under developed country" OR "under developed countries" OR "under developed nations" OR "under developed world" OR "underdeveloped country" OR "underdeveloped countries" OR "underdeveloped nation" OR "underdeveloped nations" OR "underdeveloped population" OR "underdeveloped populations" OR "underdeveloped world" OR "middle income country" OR "middle income countries" OR "middle income nation" OR "middle income nations" OR "middle income population" OR "middle income populations" OR "low income country" OR "low income countries" OR "low income nation" OR "low income nations" OR "low income population" OR "low income populations" OR "lower income country" OR "lower income countries" OR "lower income nations" OR "lower income population" OR "lower income populations" OR "underserved countries" OR "underserved nations" OR "underserved population" OR "underserved populations" OR "under served population" OR "under served populations" OR "deprived countries" OR "deprived population" OR "deprived populations" OR "poor country" OR "poor countries" OR "poor nation" OR "poor nations" OR "poor population" OR "poor populations" OR "poor world" OR "poorer countries" OR "poorer nations" OR "poorer population" OR "poorer populations" OR "developing economy" OR "developing economies" OR "less developed economy" OR "less developed economies" OR "underdeveloped economies" OR "middle income economy" OR "middle income economies" OR "low income economy" OR "low income economies" OR "lower income economies" OR "low gdp" OR "low gnp" OR "low gross domestic" OR "low gross national" OR "lower gdp" OR "lower gross domestic" OR lmic OR lmics OR "third world" OR "lami country" OR "lami countries" OR "transitional country" OR "transitional countries" OR "emerging economies" OR "emerging nation" OR "emerging nations" OR "low resource" OR austere)

OR

AB ("developing country" OR "developing countries" OR "developing nation" OR "developing nations" OR "developing population" OR "developing populations" OR "developing world" OR "less developed country" OR "less developed countries" OR "less developed nation" OR "less developed nations" OR "less developed world" OR "lesser developed countries" OR "lesser developed nations" OR "under developed country" OR "under developed countries" OR "under developed nations" OR "under developed world" OR "underdeveloped country" OR "underdeveloped countries" OR "underdeveloped nation" OR "underdeveloped nations" OR "underdeveloped population" OR "underdeveloped populations" OR "underdeveloped world" OR "middle income country" OR "middle income countries" OR "middle income nation" OR "middle income nations" OR "middle income population" OR "middle income populations" OR "low income country" OR "low income countries" OR "low income nation" OR "low income nations" OR "low income population" OR "low income populations" OR "lower income country" OR "lower income countries" OR "lower income nations" OR "lower income population" OR "lower income populations" OR "underserved countries" OR "underserved nations" OR "underserved population" OR "underserved populations" OR "under served population" OR "under served populations" OR "deprived countries" OR "deprived population" OR "deprived populations" OR "poor country" OR "poor countries" OR "poor nation" OR "poor nations" OR "poor population" OR "poor populations" OR "poor world" OR "poorer countries" OR "poorer nations" OR "poorer population" OR "poorer populations" OR "developing economy" OR "developing economies" OR "less developed economy" OR "less developed economies" OR "underdeveloped economies" OR "middle income economy" OR "middle income economies" OR "low income economy" OR "low income economies" OR "lower income economies" OR "low gdp" OR "low gnp" OR "low gross domestic" OR "low gross national" OR "lower gdp" OR "lower gross domestic" OR lmic OR lmics OR "third world" OR "lami country" OR "lami countries" OR "transitional country" OR "transitional countries" OR "emerging economies" OR "emerging nation" OR "emerging nations" OR "low resource" OR austere)

OR

TI ("Afghan" OR "Afghans" OR "Afghani" OR "Albanian" OR "Albanians" OR "Algerian" OR "Algerians" OR "American Samoan" OR "American Samoans" OR "Angolan" OR "Angolans" OR "Antiguan" OR "Antiguans" OR "Barbudan" OR "Berbudans" OR "Argentine" OR "Argentines" OR "Argentinian" OR "Argentinians" OR "Argentinean" OR "Argentineans" OR "Armenian" OR "Armenians" OR "Aruban" OR "Arubans" OR "Azerbaijani" OR "Azerbaijanis" OR "Bahraini" OR "Bahrainis" OR "Bangladeshi" OR "Bangladeshis" OR "Bangalees" OR "Bajan" OR "Bajans" OR "Barbadian" OR "Barbadians" OR "Belarusian" OR "Belarusians" OR "Byelorussian" OR "Byelorussians" OR "Belizean" OR "Belizeans" OR "Beninese" OR "Benineses" OR "Bhutanese" OR "Bolivian" OR "Bolivians" OR "Bosnian" OR "Bosnians" OR "Botswana" OR "Batswana" OR "Brazilian" OR "Brazilians" OR "Brasilian" OR "Brasilians" OR "Bulgarian" OR "Bulgarians" OR "Burkinabe" OR "Burkinese" OR "Burundian" OR "Burundians" OR "Cape Verdean" OR "Cape Verdeans" OR "Cabo Verdean" OR "Cabo Verdeans" OR "Cambodian" OR "Cambodians" OR "Khmer" OR "Cameroonian" OR "Cameroonians" OR "Central African" OR "Central Africans" OR "Chadian" OR "Chadians" OR "Chilean" OR "Chileans" OR "Colombian" OR "Colombians" OR "Comorian" OR "Comorians" OR "Congolese" OR "Costa Rican" OR "Costa Ricans" OR "Ivorian" OR "Ivorians" OR "Croatian" OR "Croatians" OR "Cuban" OR "Cubans" OR "Cypriot" OR "Cypriots" OR "Czech" OR "Czechs" OR "Djiboutian" OR "Djiboutians" OR "Dominican" OR "Dominicans" OR "Ecuadorian" OR "Ecuadorians" OR "Egyptian" OR "Egyptians" OR "Salvadoran" OR "Salvadorans" OR "Equatorial Guinean" OR "Equatorial Guineans" OR "Equatoguinean" OR "Equatoguineans" OR "Eritrean" OR "Eritreans" OR "Estonian" OR "Estonians" OR "Swazi" OR "Swazis" OR "Swati" OR "Swatis" OR "Ethiopian" OR "Ethiopians" OR "Fijian" OR "Fijians" OR "Gabonese" OR "Gabonaise" OR "Gambian" OR "Gambians" OR "Georgian" OR "Georgians" OR "Ghanaian" OR "Ghanaians" OR "Gibraltarian" OR "Gibraltarians" OR "Greek" OR "Greeks" OR "Grenadian" OR "Grenadians" OR "Guamanian" OR "Guamanians" OR "Guatemalan" OR "Guatemalans" OR "Guinean" OR "Guineans" OR "Bissau Guinean" OR "Bissau Guineans" OR "Guyanese" OR "Haitian" OR "Haitians" OR "Honduran" OR "Hondurans" OR "Indian" OR "Indians" OR "Indonesian" OR "Indonesians" OR "Iranian" OR "Iranians" OR "Iraqian" OR "Iraqians" OR "Iraqi" OR "Iraqis" OR "Manx" OR "Jamaican" OR "Jamaicans" OR "Jordanian" OR "Jordanians" OR "Kazakhstani" OR "Kazakhstanis" OR "Kenyan" OR "Kenyans" OR "Kirabati" OR "Kirabatian" OR "Kirabatians" OR "Korean" OR "Koreans" OR "Kosovar" OR "Kosovars" OR "Kosovan" OR "Kosovans" OR "Kyrgyzstani" OR "Kyrgyzstanis" OR "Kyrgyz" OR "Lao" OR "Laotian" OR "Laotians" OR "Latvian" OR "Latvians" OR "Lebanese" OR "Lesothan" OR "Lesothans" OR "Lesothonian" OR "Lesothonians" OR "Mosotho" OR "Basotho" OR "Liberian" OR "Liberians" OR "Libyan" OR "Libyans" OR "Lithuanian" OR "Lithuanians" OR "Macanese" OR "Macedonian" OR "Macedonians" OR "Malagasy" OR "Madagascan" OR "Madagascans" OR "Malawian" OR "Malawians" OR "Malay" OR "Malaya" OR "Malaysian" OR "Malaysians" OR "Maldivian" OR "Maldivians" OR "Malian" OR "Malians" OR "Maltese" OR "Marshallese" OR "Marshalleses" OR "Mauritanian" OR "Mauritanians" OR "Mauritian" OR "Mauritians" OR "Mexican" OR "Mexicans" OR "Micronesian" OR "Micronesians" OR "Moldovan" OR "Moldovans" OR "Mongolian" OR "Mongolians" OR "Mongol" OR "Montenegrin" OR "Montenegrins" OR "Moroccan" OR "Moroccans" OR "Mozambican" OR "Mozambicans" OR "Burmese" OR "Myanma" OR "Namibian" OR "Namibians" OR "Nauruan" OR "Nauruans" OR "Nepali" OR "Nepalese" OR "Netherlands Antillean" OR "Netherlands Antilleans" OR "Nicaraguan" OR "Nicaraguans" OR "Nigerien" OR "Nigeriens" OR "Nigerian" OR "Nigerians" OR "Northern Mariana Islander" OR "Northern Mariana Islanders" OR "Mariana" OR "Marianas" OR "Omani" OR "Omanis" OR "Pakistani" OR "Pakistanis" OR "Palauan" OR "Palauans" OR "Palestinian" OR "Palestinians" OR "Panamanian" OR "Panamanians" OR "Papua New Guinean" OR "Papua New Guineans" OR "Paraguayan" OR "Paraguayans" OR "Peruvian" OR "Peruvians" OR "Philippine" OR "Philippines" OR "Philipine" OR "Philipines" OR "Phillipine" OR "Phillipines" OR "Phillippine" OR "Phillippines" OR "Filipino" OR "Filipinos" OR "Filipina" OR "Filipinas" OR "Portuguese" OR "Puerto Rican" OR "Puerto Ricans" OR "Romanian" OR "Romanians" OR "Rwandan" OR "Rwandans" OR "Rwandese" OR "Ruandan" OR "Ruandans" OR "Ruandese" OR "Samoan" OR "Samoans" OR "Sao Tomean" OR "Sao Tomeans" OR "Santomean" OR "Santomeans" OR "Senegalese" OR "Montenegrin" OR "Montenegrins" OR "Seychellois" OR "Seychelloise" OR "Seychelloises" OR "Sierra Leonean" OR "Sierra Leoneans" OR "Solomon Islander" OR "Solomon Islanders" OR "Somali" OR "Somalis" OR "South African" OR "South Africans" OR "South Sudanese" OR "Sri Lankan" OR "Sri Lankans" OR "Ceylonese" OR "Kittitian" OR "Kittitians" OR "Nevisian" OR "Nevisians" OR "Saint Lucian" OR "Saint Lucians" OR "Vincentian" OR "Vincentians" OR "Sudanese" OR "Surinamese" OR "Surinameses" OR "Syrian" OR "Syrians" OR "Tajik" OR "Tajiks" OR "Tajikistani" OR "Tajikistanis" OR "Tanzanian" OR "Tanzanians" OR "Tanganyikan" OR "Tanganyikans" OR "Thai" OR "Timorese" OR "Timoreses" OR "Togolese" OR "Tongan" OR "Tongans" OR "Trinidadian" OR "Trinidadians" OR "Tobagonian" OR "Tobagonians" OR "Tunisian" OR "Tunisians" OR "Turkmen" OR "Turkmens" OR "Tuvaluan" OR "Tuvaluans" OR "Ugandan" OR "Ugandans" OR "Ukrainian" OR "Ukrainians" OR "Uruguayan" OR "Uruguayans" OR "Uzbek" OR "Uzbeks" OR "Vanuatu" OR "Vanuatuan" OR "Vanuatuans" OR "Venezuelan" OR "Venezuelans" OR "Vietnamese" OR "Yemeni" OR "Yemenis" OR "Yemenite" OR "Yemenites" OR "Yemenese" OR "Yugoslav" OR "Yugoslavs" OR "Yugoslavian" OR "Yugoslavians" OR "Zambian" OR "Zambians" OR "Zimbabwean" OR "Zimbabweans")

OR

AB ("Afghan" OR "Afghans" OR "Afghani" OR "Albanian" OR "Albanians" OR "Algerian" OR "Algerians" OR "American Samoan" OR "American Samoans" OR "Angolan" OR "Angolans" OR "Antiguan" OR "Antiguans" OR "Barbudan" OR "Berbudans" OR "Argentine" OR "Argentines" OR "Argentinian" OR "Argentinians" OR "Argentinean" OR "Argentineans" OR "Armenian" OR "Armenians" OR "Aruban" OR "Arubans" OR "Azerbaijani" OR "Azerbaijanis" OR "Bahraini" OR "Bahrainis" OR "Bangladeshi" OR "Bangladeshis" OR "Bangalees" OR "Bajan" OR "Bajans" OR "Barbadian" OR "Barbadians" OR "Belarusian" OR "Belarusians" OR "Byelorussian" OR "Byelorussians" OR "Belizean" OR "Belizeans" OR "Beninese" OR "Benineses" OR "Bhutanese" OR "Bolivian" OR "Bolivians" OR "Bosnian" OR "Bosnians" OR "Botswana" OR "Batswana" OR "Brazilian" OR "Brazilians" OR "Brasilian" OR "Brasilians" OR "Bulgarian" OR "Bulgarians" OR "Burkinabe" OR "Burkinese" OR "Burundian" OR "Burundians" OR "Cape Verdean" OR "Cape Verdeans" OR "Cabo Verdean" OR "Cabo Verdeans" OR "Cambodian" OR "Cambodians" OR "Khmer" OR "Cameroonian" OR "Cameroonians" OR "Central African" OR "Central Africans" OR "Chadian" OR "Chadians" OR "Chilean" OR "Chileans" OR "Colombian" OR "Colombians" OR "Comorian" OR "Comorians" OR "Congolese" OR "Costa Rican" OR "Costa Ricans" OR "Ivorian" OR "Ivorians" OR "Croatian" OR "Croatians" OR "Cuban" OR "Cubans" OR "Cypriot" OR "Cypriots" OR "Czech" OR "Czechs" OR "Djiboutian" OR "Djiboutians" OR "Dominican" OR "Dominicans" OR "Ecuadorian" OR "Ecuadorians" OR "Egyptian" OR "Egyptians" OR "Salvadoran" OR "Salvadorans" OR "Equatorial Guinean" OR "Equatorial Guineans" OR "Equatoguinean" OR "Equatoguineans" OR "Eritrean" OR "Eritreans" OR "Estonian" OR "Estonians" OR "Swazi" OR "Swazis" OR "Swati" OR "Swatis" OR "Ethiopian" OR "Ethiopians" OR "Fijian" OR "Fijians" OR "Gabonese" OR "Gabonaise" OR "Gambian" OR "Gambians" OR "Georgian" OR "Georgians" OR "Ghanaian" OR "Ghanaians" OR "Gibraltarian" OR "Gibraltarians" OR "Greek" OR "Greeks" OR "Grenadian" OR "Grenadians" OR "Guamanian" OR "Guamanians" OR "Guatemalan" OR "Guatemalans" OR "Guinean" OR "Guineans" OR "Bissau Guinean" OR "Bissau Guineans" OR "Guyanese" OR "Haitian" OR "Haitians" OR "Honduran" OR "Hondurans" OR "Indian" OR "Indians" OR "Indonesian" OR "Indonesians" OR "Iranian" OR "Iranians" OR "Iraqian" OR "Iraqians" OR "Iraqi" OR "Iraqis" OR "Manx" OR "Jamaican" OR "Jamaicans" OR "Jordanian" OR "Jordanians" OR "Kazakhstani" OR "Kazakhstanis" OR "Kenyan" OR "Kenyans" OR "Kirabati" OR "Kirabatian" OR "Kirabatians" OR "Korean" OR "Koreans" OR "Kosovar" OR "Kosovars" OR "Kosovan" OR "Kosovans" OR "Kyrgyzstani" OR "Kyrgyzstanis" OR "Kyrgyz" OR "Lao" OR "Laotian" OR "Laotians" OR "Latvian" OR "Latvians" OR "Lebanese" OR "Lesothan" OR "Lesothans" OR "Lesothonian" OR "Lesothonians" OR "Mosotho" OR "Basotho" OR "Liberian" OR "Liberians" OR "Libyan" OR "Libyans" OR "Lithuanian" OR "Lithuanians" OR "Macanese" OR "Macedonian" OR "Macedonians" OR "Malagasy" OR "Madagascan" OR "Madagascans" OR "Malawian" OR "Malawians" OR "Malay" OR "Malaya" OR "Malaysian" OR "Malaysians" OR "Maldivian" OR "Maldivians" OR "Malian" OR "Malians" OR "Maltese" OR "Marshallese" OR "Marshalleses" OR "Mauritanian" OR "Mauritanians" OR "Mauritian" OR "Mauritians" OR "Mexican" OR "Mexicans" OR "Micronesian" OR "Micronesians" OR "Moldovan" OR "Moldovans" OR "Mongolian" OR "Mongolians" OR "Mongol" OR "Montenegrin" OR "Montenegrins" OR "Moroccan" OR "Moroccans" OR "Mozambican" OR "Mozambicans" OR "Burmese" OR "Myanma" OR "Namibian" OR "Namibians" OR "Nauruan" OR "Nauruans" OR "Nepali" OR "Nepalese" OR "Netherlands Antillean" OR "Netherlands Antilleans" OR "Nicaraguan" OR "Nicaraguans" OR "Nigerien" OR "Nigeriens" OR "Nigerian" OR "Nigerians" OR "Northern Mariana Islander" OR "Northern Mariana Islanders" OR "Mariana" OR "Marianas" OR "Omani" OR "Omanis" OR "Pakistani" OR "Pakistanis" OR "Palauan" OR "Palauans" OR "Palestinian" OR "Palestinians" OR "Panamanian" OR "Panamanians" OR "Papua New Guinean" OR "Papua New Guineans" OR "Paraguayan" OR "Paraguayans" OR "Peruvian" OR "Peruvians" OR "Philippine" OR "Philippines" OR "Philipine" OR "Philipines" OR "Phillipine" OR "Phillipines" OR "Phillippine" OR "Phillippines" OR "Filipino" OR "Filipinos" OR "Filipina" OR "Filipinas" OR "Portuguese" OR "Puerto Rican" OR "Puerto Ricans" OR "Romanian" OR "Romanians" OR "Rwandan" OR "Rwandans" OR "Rwandese" OR "Ruandan" OR "Ruandans" OR "Ruandese" OR "Samoan" OR "Samoans" OR "Sao Tomean" OR "Sao Tomeans" OR "Santomean" OR "Santomeans" OR "Senegalese" OR "Montenegrin" OR "Montenegrins" OR "Seychellois" OR "Seychelloise" OR "Seychelloises" OR "Sierra Leonean" OR "Sierra Leoneans" OR "Solomon Islander" OR "Solomon Islanders" OR "Somali" OR "Somalis" OR "South African" OR "South Africans" OR "South Sudanese" OR "Sri Lankan" OR "Sri Lankans" OR "Ceylonese" OR "Kittitian" OR "Kittitians" OR "Nevisian" OR "Nevisians" OR "Saint Lucian" OR "Saint Lucians" OR "Vincentian" OR "Vincentians" OR "Sudanese" OR "Surinamese" OR "Surinameses" OR "Syrian" OR "Syrians" OR "Tajik" OR "Tajiks" OR "Tajikistani" OR "Tajikistanis" OR "Tanzanian" OR "Tanzanians" OR "Tanganyikan" OR "Tanganyikans" OR "Thai" OR "Timorese" OR "Timoreses" OR "Togolese" OR "Tongan" OR "Tongans" OR "Trinidadian" OR "Trinidadians" OR "Tobagonian" OR "Tobagonians" OR "Tunisian" OR "Tunisians" OR "Turkmen" OR "Turkmens" OR "Tuvaluan" OR "Tuvaluans" OR "Ugandan" OR "Ugandans" OR "Ukrainian" OR "Ukrainians" OR "Uruguayan" OR "Uruguayans" OR "Uzbek" OR "Uzbeks" OR "Vanuatu" OR "Vanuatuan" OR "Vanuatuans" OR "Venezuelan" OR "Venezuelans" OR "Vietnamese" OR "Yemeni" OR "Yemenis" OR "Yemenite" OR "Yemenites" OR "Yemenese" OR "Yugoslav" OR "Yugoslavs" OR "Yugoslavian" OR "Yugoslavians" OR "Zambian" OR "Zambians" OR "Zimbabwean" OR "Zimbabweans")**)**

NOT (SO ("Plant Disease"))

Limits: January 2000 – December 2021

**Supplement 2. Review protocol**

**Review title**

Prehospital trauma care for explosive ordnance-related injuries in low-resource settings: a systematic review

**Review rationale and objective**

The objective of this review is to perform a structured evaluation of trauma interventions in low-resource and austere settings with a focus on victims of blast injury. By assessing prior efforts in this domain, this review will synthesize what is known about interventions that have demonstrated potential to reduce morbidity and mortality among populations affected by explosive ordnance. By conducting this review, we intend to identify prior successes, barriers, and gaps that may be used to inform programming for actors within the mine action sector to strengthen the emergency health response to blast injury.

**Study design**

Given the limited number of reports relevant to the subject of interest identified by preliminary database searches, we will perform a structured search of the published literature, and records from non-peer-reviewed academic literature such as non-governmental organization (NGO) reports and policy documents will also be included in this review.

**Search strategy and information sources**

We will utilize a structured search strategy implementing database-specific language and structured index terms designed to capture all records describing interventions focused on prehospital trauma care in low-resource settings and the prehospital care of victims injured by blast mechanisms. In addition to reports retrieved by the above search strategy, reference lists of records meeting eligibility criteria will be screened for additional relevant results.

We will search the following electronic bibliographic databases:

- PUBMED/MEDLINE
- EMBASE (Elsevier)
- CINAHL (EBSCO)
- Global Index Medicus
- CABI Global Health (EBSCO)
- Cochrane Library (Wiley)
- Web of Science Core Collection – SCI-EXPANDED, SSCI, AHCI, ESCI

In addition, we will search the following organizational websites and grey literature repositories:

- WHO
- IOM
- UNHCR
- Google Scholar

**Eligibility criteria**

Inclusion: Eligible studies will describe trauma interventions and trauma care systems or the prehospital care of trauma patients or victims of blast injury in austere/low-resource settings. Patient population will be limited to local nationals from low- and middle-income countries (LMICs) as defined by the World Bank economic classification, or countries affected by active armed conflict. Local combatants are eligible for inclusion, while reports describing the injuries of/care rendered to military servicemembers of high-income countries will be excluded from this review. Given the limited extent of available data on the subject of interest, no studies will be excluded from consideration based on type of study design.

Exclusion: To remain pertinent to humanitarian care in the setting of contemporary armed conflict, date restrictions will limit results to 21^st^ century literature (i.e. records published before the year 2000 will be excluded). Reports discussing mental health interventions will be excluded. Reports pertaining to high-resource settings will be excluded. No studies will be excluded on the basis of language. Records without full-text availability will be excluded from analysis.

**Data selection**

Records identified through database searches will be screened by title and abstract for relevance to eligibility criteria by two independent reviewers, who will consult a senior reviewer to arbitrate any discrepancies. After verifying relevance to eligibility criteria and excluding duplicate data presented from previously published reports, full-text articles will be retrieved for the remaining candidate records. Two independent reviewers will complete the screening of full-text articles, consulting a third reviewer to arbitrate discrepancies and reach majority consensus. References of articles ultimately identified as meeting all inclusion criteria will then be screened for relevant citations, from which additional records meeting eligibility criteria will be included.

**Data items, extraction, and management**

In order to synthesize elements most pertinent to the review question, after completion of the screening process data extraction will be conducted using a standardized extraction form by two reviewers for the purposes of subsequent narrative synthesis. The following information will be extracted from studies included in the review if presented:

- Study information: year, location/setting, conflict, study design and methods, duration
- Program/intervention sponsor: e.g. NGO, local government, etc.
- Study sample: patient population, sample size, demographics (e.g. age, sex, ethnic group, special populations)
- Description of patient injuries: mechanism of injury, anatomic region of injury, grading of injury severity if available
- Description of intervention
- Resource utilization
- Outcomes: mortality, functional disability, complications, prehospital time
- Metrics of intervention effectiveness

**Risk of bias (quality) assessment**

Given the limited extent of available data on the subject of interest, reports will not be excluded from consideration based on assessment of data quality or potential bias.

**Strategy for data synthesis and extraction**

Due to the heterogeneity of reports on this topic, we anticipate that there will be a limited role for meta-analysis. Both quantitative and structured qualitative analysis of retrieved reports will be performed to extract information most pertinent to the focused research question. Results will be reported in accordance with PRISMA guidelines. Data will be synthesized qualitatively in narrative and tabular format. Where possible, report characteristics will be pooled and presented as a summary of findings table for the primary variables of interest.

**Keywords**

Prehospital trauma care; blast injury; explosive ordnance; trauma systems; low-resource settings; austere environments

**Supplement 3. PRISMA-SCr Checklist**

**
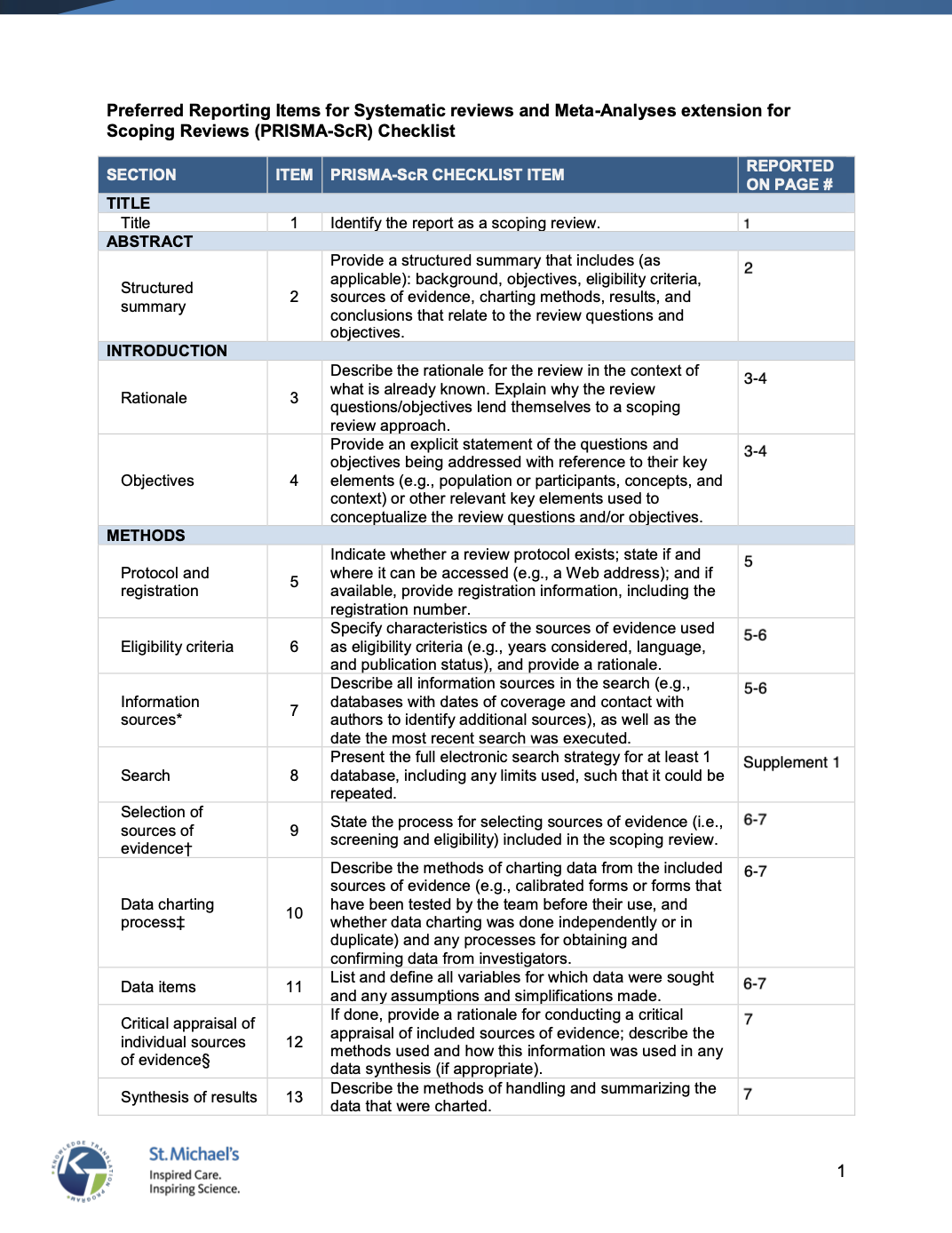
**


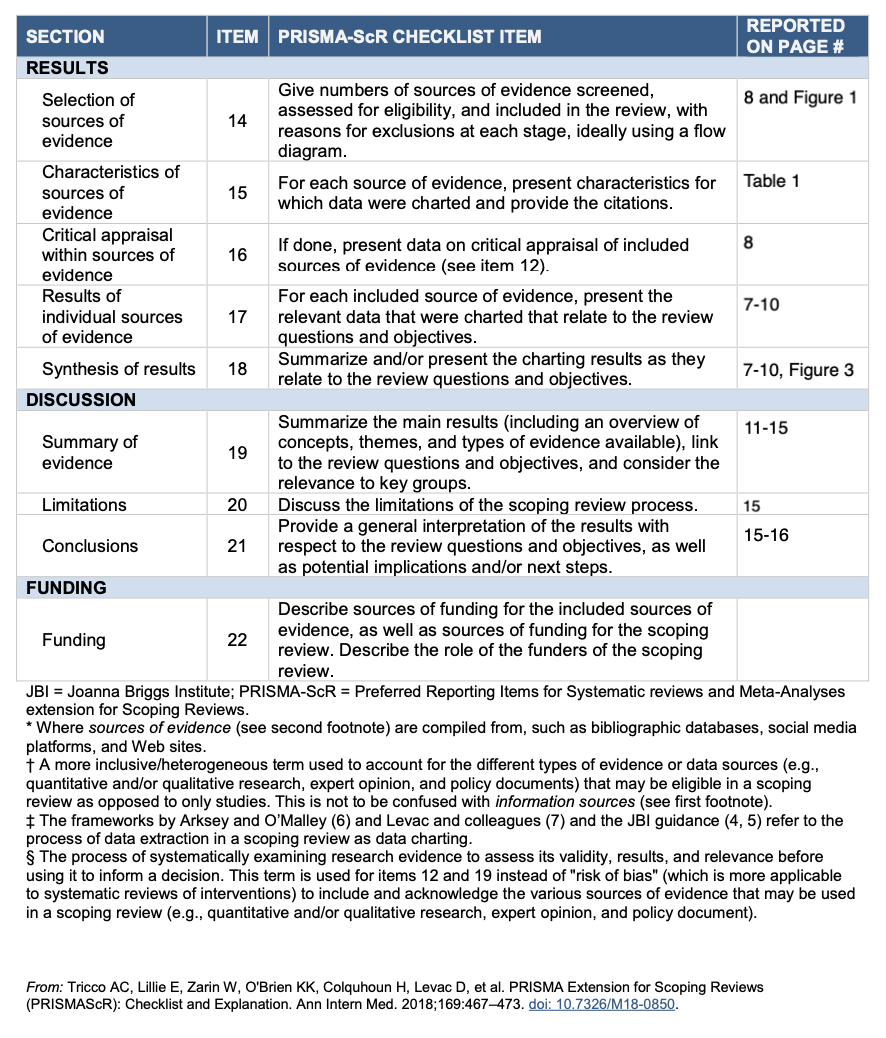


**Supplement 4. Descriptive summary of reports included in analysis**

| **Author** | **Year** | **Country** | **Summary** | **Outcomes** | **QuADS Score*** |
| --- | --- | --- | --- | --- | --- |
| Abib [1] | 2006 | Brazil | The PHPTC was compared to the Glasgow Coma Score and Revised Trauma Score and was found to be a reliable method for assessment and triage of pediatric trauma resources. | The PHPTC was found to be useful for rapid assessment, triage and resource allocation in the prehospital setting and had a significant association with the Glasgow Coma Score and Revised Trauma Score (p<0.0001.) | 16 |
| Aboutanos [2] | 2007 | Ecuador | A basic trauma course for practitioners in rudimentary health posts in Ecuador reported improvements in prehospital care, extremity injury care, and patient evaluation. | Test scores significantly improved from pretest to post-test (72% to 79%.) In rudimentary health posts 76% of physicians passed, management was adequate for hemorrhage control (65%), immobilization (77%), and early transfer (92%.) | 36 |
| Abraham [3] | 2015 | N/A | A first responder training program Massive Open Online Course Trauma Training was proposed in India. | The course was deemed to be a no-cost, high-quality education, targeted for an expansive audience of laypersons with sustainable dissemination through a “train-the-trainer” approach. | 5 |
| Adolfi [4] | 2010 | Brazil | An emergency coordination system model was implemented in São Paulo to promote coordination among local emergency medicine systems. | Model implementation facilitated the development of indicators that allowed monitoring of the emergency coordination process for quality improvement and systems advances in Brazil. | 32 |
| Aekka [5] | 2015 | India | A two-day course for non-physician first responders significantly improved competence in trauma management. | Statistically significant improvement in all areas of trauma management was observed including airway, hemorrhage control, extrication, scene assessment, triage, and communication. | 29 |
| Agrawal [6] | 2012 | India | The creation of a call center in New Delhi, India that was integrated with the medical record system of a level 1 trauma center improved patient satisfaction, waiting times, and reduced unnecessary follow-up visits. | Patient satisfaction increased from 32% to 96% following implementation of call center operations. 95% reported decreased waiting time in clinics. The initial cost of outsourcing the call center was USD $4,000 with a recurring cost of $2,000 per month. | 11 |
| Alexandrino [7] | 2020 | Brazil, Portugal | The joint DSTC and DATC courses resulted in an increase in self-assessed importance of team briefing and intraoperative communication. | Prior to the course, 36% of participants agreed that a pre-operative briefing was very important compared to 91% post-course. The authors highlight the importance of nontechnical components of damage control trauma surgery. | 30 |
| Ali [8] | 1993 | Trinidad & Tobago | ATLS training in a tertiary hospital in Trinidad and Tobago decreased overall and ICU mortality. | Mortality for both blunt and penetrating injuries was higher in the pre-ATLS group (77% pre- versus 46% post-ATLS for blunt, 20% pre- vs. 6% post-ATLS for penetrating). Observed to expected mortality ratio decreased (pre- 3; post-ATLS 2). | 14 |
| Ali [9] | 1997 | Trinidad & Tobago | The PHTLS program in Trinidad and Tobago demonstrated improvements in the performance of numerous prehospital interventions. | Post-PHTLS frequency increased for airway control (10 vs. 100%), C-spine control (2 vs. 89%), splinting (22 vs. 61%), hemorrhage control (16 vs. 97%), and oxygen use (7 vs. 89.5%.) | 16 |
| Ali [10] | 1994 | Trinidad & Tobago | ATLS training at a tertiary hospital in Trinidad and Tobago signiﬁcantly improved the appropriate use of vital ED interventions. | The pre-ATLS vs. post-ATLS frequencies of endotracheal intubation (83.5% vs. 65%), nasogastric tube insertion (68% vs. 91%), Foley catheterization (75% vs. 96%) and chest tube insertion (18% vs. 47%) were statistically significant. | 14 |
| Alizadeh [11] | 2012 | Iran | A protocol to triage trauma calls at an EMS system in Iran was implemented. | The implementation of this trauma dispatch algorithm was reduced the number of unnecessary ambulance runs by 16%. | 20 |
| Al Sabah [12] | 2018 | Kuwait | A "stop the bleed" training campaign administered in Kuwait increased utilization of first-aid skills as well as perceived competence in implementing those skills. | Most participants demonstrated knowledge of hemorrhage control and tourniquet application, and 89% felt the campaign was useful for raising awareness. | 9 |
| Altamirano [13] | 2020 | Ecuador | A novel Trauma and Acute Care Surgery model implemented in Ecuador decreased surgical wait time, length of stay, and the mortality. | A statistically significant decrease in surgical wait time (10.6–3.2 hours for emergency general surgery, 6.3–1.6 hours for trauma) was observed as well as reduction in mortality from 1.1 to 0.86%. | 17 |
| Altintas [14] | 1999 | Turkey | A cost-effectiveness analysis of the development of a citywide ambulance service in Ankara, Turkey was conducted. | The costs per year were USD $85,170 and recurrent costs were $833,710. The cost per ambulance run was $163; the cost per injured patient was $181. | 5 |
| Alto [15] | 2007 | Brazil | Implementation of the ATLS Program in Brazil reported a 17% reduction in mortality. | A statistically significant reduction of 17% in the group post-ATLS in mortality was observed. | 4 |
| Amiri [16] | 2013 | Iran | Implementation of Prehospital Trauma Care training in a tertiary hospital in Iran resulted in improved knowledge and skill retention. | Pretest, early post-test, and late post-test scores were 18.84, 26.7, and 22.2, respectively (p< 0.001). Hands-on patient scenarios improved primary survey performance. | 20 |
| Anderson [17] | 2018 | Uganda | The Emergency Ward Management of Trauma (EWMT) and Surgical Techniques and Repairs in Trauma for the Low-resource Environment (STaRTLE) courses for providers in Uganda significantly improved participant knowledge. | EMWT participants felt better prepared to care for the injured patient (median Likert [IQR] 5.0). Similarly, following the STaRTLE course, participants felt significantly more comfortable with performing 20 of the 22 operative procedures taught. | 8 |
| Arnson [18] | 2009 | Iran | After reviewing data on the human cost of landmines the authors underscored the impact of broad-reaching educational programs in reducing mortality. | To reduce morbidity and mortality caused by landmines, the authors recommend: discontinuation of production and use of landmines; detection and clearance; mine risk education for threatened populations; and assistance to victims of landmines. | 2 |
| Arreola-Risa [19] | 2004 | Mexico | Cost-effectiveness comparison of prehospital training models between two ambulance services in Mexico demonstrated process improvements at both sites but showed a reduction in mortality only in the service that implemented the simplest and lowest-cost interventions. | The training was effective for both sites, with increases in basic airway maneuvers in Monterey (16% pre- versus 39% post-) and San Pedro (14% versus 64%). Mortality decreased only in Monterrey (8.2% pre- versus 4.7% post.) Costs ranged from US$150–400 per medic trained. | 20 |
| Arreola-Risa [20] | 2000 | Mexico | An increase in prehospital dispatch sites and implementation of a PHTLS course reduced response time and prehospital mortality. | The introduction of the PHTLS course resulted in increased use of prehospital interventions (e.g., airway management, placement of IV catheters.) Together, these interventions reduced prehospital mortality among transported patients from 8.2% to 4.7%. | 31 |
| Arreola-Risa [21] | 1995 | Mexico, US | A modeling study compared prehospital and ED care structure, processes, and outcomes between cities and tertiary hospitals in the US and Mexico. Findings led the authors to recommend targeting prehospital and ED care with interventions to reduce mortality. | Mortality was higher in the Monterey than Seattle (55% vs. 34%.) In Monterrey, 40% of seriously injured patients died in the field and 11% in the ED, compared with 21% and 6% in Seattle. Scene and transport times were < 30 minutes for 47% of Monterrey cases vs. 75% in Seattle. | 27 |
| Arreola-Risa [22] | 2007 | Mexico | A 3-month Emergency Medical Technician training program in Mexico decreased pre-hospital mortality by 45% and improvements in the use of prehospital interventions (e.g., airway management, use of IV ﬂuids). | Mortality decreased from 1.8% to 0.5% after the training. The 3-month program cost US$200-600 per medic compared to 2-3 day BTLS courses that are approximately $150. | 36 |
| Balhara [23] | 2019 | N/A | A systematic review of layperson aid or transport for trauma victims in LMICs reported high rates of bystander involvement in prehospital trauma transport and care | All studies demonstrated high rates of bystander involvement in prehospital trauma care and transport in LMICs, highlighting the need for bystander training and the “train the trainer” models. | 27 |
| Bandyo-padhyay [24] | 2017 | India | An educational intervention on first aid among middle school students in West Bengal was found to be effective. | A significant change in knowledge from pretest score (mean = 1.50) to posttest score (mean = 6.53) was noted. | 18 |
| Barss [25] | 1983 | Papua New Guinea | An aeromedical prehospital care system was implemented in Papua New Guinea. | It was estimated that 63 of 92 patients would have died without this service. The average cost per life saved was approximately USD $520 and the average cost of providing the emergency service per population per year was about $0.12. | 5 |
| Barthel [26] | 2011 | N/A | A model was created using data collected by the Israeli Defense Forces' response to the Haiti earthquake of 2010 to estimate the impact of a pediatric trauma center. | It was estimated that the availability of a pediatric trauma center could reduce the time to treatment by approximately half, the time to treat all children affected by the disaster by more than a third, and result in a relative mortality risk reduction of 37%. | 31 |
| Bertol [27] | 2014 | Haiti, DRC, Afghanistan | A study conducted in MSF programs in the DRC, Afghanistan, and Haiti showed that availability of external fixation resources and a training program for non-specialist surgeons reduced amputation rates. | In the post-earthquake response in Haiti, 81% of open fractures were treated by amputation. This program demonstrated a decrease in amputation rates among open fractures from 100% to 21% in the DRC and 20% to <10% in Haiti and Afghanistan. | 36 |
| Bissell [28] | 2004 | US, Armenia, Japan | A modeling study described differences between cities affected by earthquakes in the US, Japan, and Armenia. | Deaths-per-100-injuries ratios from each city after their respective earthquakes were better in the HICs owing to the structural differences in disaster preparedness and response. | 8 |
| Boeck [29] | 2018 | Bolivia | An 8-hour layperson Trauma First Responder course (TFRC) in Bolivia resulted in increased participant knowledge and skill. | Median test scores improved after course completion (48% vs. 76%), along with skill confidence (Likert 4 vs. 4.5.) | 23 |
| Boeck [30] | 2015 | Bolivia | A TFRC in Bolivia reported a significant increase in important injury management knowledge. | Overall median test scores increased significantly after course completion across the cohort (40% vs. 80%). | 21 |
| Brooks [31] | 1999 | South Africa | The impact of an educational symposium on universal barrier precautions and risk of occupational transmission of HIV was evaluated in a trauma unit in South Africa. | A significant improvement in compliance from 48% to 74% after the symposium (p = 0.007), with specific improvement in the wearing of masks and visors was reported. | 27 |
| Brown [32] | 2016 | India | A public-private emergency prehospital training for medical technicians accompanied by public awareness campaigns was deemed successful. | The authors found the public-private partnership training model to be responsive to the needs of the population while sustaining low operational costs. | 5 |
| Bruijns [33] | 2008 | South Africa | The Cape Triage Score was evaluated prospectively in South Africa at a secondary referral hospital. | Modifications to the triage score reduced undertriage from 24% to 12%. | 34 |
| Buntman [34] | 2002 | South Africa | The introduction of helicopter transport in the prehospital care system in South Africa reported a decrease in preventable deaths | Helicopter transport reduced preventable mortality by 21% compared to the road-transfer model. | 22 |
| Cai [35] | 2014 | China | Comparison between level 1 trauma centers in China and the US suggested that system organization and center designation may positively impact mortality. | The adoption of ACS-COT standard in the accreditation of level 1 trauma centers in China may reduce injury-related death. | 31 |
| Calderale  [36] | 2008 | Romania, Italy | An audit of level 1 trauma centers in Italy and Romania demonstrated structural differences and higher ISS-adjusted mortality rates in Romania. | The authors suggest that increased infrastructure and specialist care availability and improved documentation and transfer processes could decrease trauma mortality rates in Romania. | 17 |
| Callese [37] | 2014 | N/A | A systematic review examined trauma educational initiatives for layperson first responders in resource-poor settings. | Recommended course design includes: needs assessment; leverage of existing resources; inclusion of participants with minimal education; and postimplementation evaluation. | 35 |
| Capone [38] | 2000 | Brazil | A study examined first aid skills acquisition by factory workers using two different means of delivery (live demonstration vs television-projected demonstrations.) | A significant proportion of factory workers acquired skills through television viewing alone, except for CPR steps B (rescue breaths) and C (external chest compressions). | 30 |
| Carlson [39] | 2012 | Haiti | A cost-effectiveness analysis of an orthopedic trauma training program for doctors in Haiti was conducted. | 12,213 DALYs were averted per fellow trained, at a cost of USD $134 per DALY which was deemed highly cost-effective. | 19 |
| Carter [40] | 2011 | Ghana | An evaluation of a trauma course conducted at a teaching hospital in Ghana reported significant improvement in provider knowledge on trauma management. | Participants demonstrated significant improvement in pre- and post-test scores (50% vs. 77.5%) including clinical skills in RSI, cricothyroidotomy, tube thoracostomy, and pericardiocentesis with significant improvement in 25 of 39 skills measures tested. | 3 |
| Chaleekrua [41] | 2011 | Thailand | A model for community-based prehospital care management for emergency volunteers in rural Thailand was conducted. | This model was deemed effective for managing emergency volunteers and built self-confidence among the participants. | 5 |
| Chittawata-narat [42] | 2013 | Thailand | A Rapid Response Trauma Team (RRTT) for thoracic injuries in a tertiary hospital in Thailand improved mortality odds ratios. | The RRTT significantly improved the mortality odds ratio in overall and severe trauma and reduced the ISS-adjusted mortality rate from 25% to 15%. The RRTT also had a positive impact on the outcomes of maxillofacial, head, and orthopedic injuries. | 34 |
| Colohan [43] | 1989 | India, US | A modeling study compared structural differences in neurosurgical care between tertiary hospitals in the US and India. | Lack of prehospital emergency care was thought to cause increased mortality after head injury in India. The authors suggest that improvements in planning and organization of EMS could decrease ISS-adjusted mortality rate from 11 to 7%. | 3 |
| De Ramirez [44] | 2014 | Uganda | A community-based prehospital training program in rural Uganda reported sustained structural changes including improved staff retention and functioning ambulances. | Cost-benefit analysis demonstrated a cost of USD $89.95 per life saved with an estimated $0.93/capita to establish the system and $0.09/capita/year to maintain the system. | 3 |
| Demetria-des [45] | 2005 | N/A | A study on trauma center designation and volume on outcome of severe injuries among level I and II trauma centers found significantly better outcomes at level I centers. | Level I centers had significantly lower mortality (25% vs 29%) and lower severe disability at discharge (20% vs 34%) than level II centers. | 34 |
| Durham [46] | 2005 | Laos | A health education and mine awareness program was deployed in Laos. | The authors advocate for community liaisons to build a structured approach to mine risk education and local perceptions of risk. | 5 |
| Duron [47] | 2014 | Peru | A locally driven trauma registry was implemented in Peru. | Improvements were observed in percentage of patients registered as well as quality of information (e.g., vital sign documentation, increase in temperature 42 to 97%, oxygen saturation 26 to 92%.) | 30 |
| El-Shinawi [48] | 2015 | Egypt | A Trauma Education Programs training program in a tertiary hospital in Egypt was implemented and scaled. | The program was successfully adopted by the Egyptian Emergency Medicine Board and continued to run for more than eight years even after transitioning to in-country leadership and trainers. | 11 |
| Erickson [49] | 1996 | Rwanda | Locally organized trauma training courses for physicians, nurses, and medical assistants at a tertiary hospital in Rwanda improved the use of ED interventions. | Specific areas of statistically significant improvement included sharps disposal, wound irrigation, and sterile technique. No impact on mortality was observed. | 16 |
| Fuzaylov [50] | 2021 | Ukraine | The impact of a multidisciplinary burn outreach program was assessed and reported a decrease in complication rates. | Major complication rates were reduced including: wound infection (7% vs. 16%), pneumonia (2.4% vs. 0.3%), sepsis (1.6% vs. 0.6%), UTI (2% vs. 0.6%), and respiratory failure (1.3% vs. 0.4%.) | 20 |
| Gallaher [51] | 2020 | Malawi | An analysis of the relationship between operative access and burn-associated mortality in a sub-Saharan African burn unit reported that surgical intervention reduced the risk of death. | Crude mortality was 3.3% and 27.0% in the operative and nonoperative groups, respectively. Surgical intervention reduced the risk of death by almost 50% for adult patients. | 34 |
| Geduld [52] | 2010 | Madagas-car | A 1-day course for taxi drivers in Madagascar taught by doctors was deemed the course a success by both faculty and candidates. | Content included prehospital scene management, hemorrhage control, fracture immobilization, and labor & delivery. Formal evaluation of knowledge and skill retention was in development. | 11 |
| Georgoff [53] | 2010 | N/A | A modeling study compared outcomes after TBI between HICs and LMICs and suggested that LMICs could improve TBI outcomes by improving spatial access to care. | LMICs have the highest mortality rates in treating patients with severe closed traumatic brain injury. Lower treatment intensity and lack of access to care contributed to this discrepancy. | 35 |
| Gosselin [54] | 2008 | Cambodia | A cost-effectiveness analysis was conducted at an NGO hospital providing orthopedic care to the war-injured and victims of landmine-related injury. | Costs for trauma surgical care were USD $77 per DALY averted, which is very cost-effective and compares favorably to other non-surgical public health interventions. | 15 |
| Gosselin [55] | 2010 | Nigeria, Haiti | A cost-effectiveness analysis was conducted at two MSF hospitals in Nigeria and Haiti. | Trauma care costs were found to be USD $172 and $223 per DALY averted at each site, in line with other reported costs for surgical and nonsurgical activities in similar contexts. | 22 |
| Hanche-Olsen [56] | 2015 | Botswana | An assessment of a training program in government hospitals in Botswana two years post-implementation reported sustained structural changes in physical and human resources and trauma administrative functions. | Most hospitals formed local trauma committees; approximately half were performing simulations regularly. Numerous hospitals had created defined trauma teams with activation criteria and developed local trauma guidelines and registries. Equipment availability (e.g. airway, cervical collar) increased. | 16 |
| Hashmi [57] | 2013 | Pakistan | A trauma team with ATLS training along with the creation of a trauma registry and a qualifying program in Pakistan signiﬁcantly reduced mortality rates. | injured patients were 4.9 times less likely to die and 2.6 times less likely to have a complication compared with those cared for before the interventions. | 35 |
| Hauswald [58] | 1998 | Malaysia | A modeling study compared prehospital spinal immobilization and neurologic outcomes between tertiary hospitals in the US and Malaysia. | There was less neurologic disability in the unimmobilized Malaysian patient cohort (OR 2.03; 95% CI 1.03-3.99; p = 0.04), with a <2 % chance that prehospital spinal immobilization has any beneficial effect. | 31 |
| Hauswald [59] | 1997 | Malaysia | The authors modeled that the development of a prehospital system comparable to the US in Kuala Lumpur, Malaysia would cost US$2.5 million per year. | A prehospital system for Kuala Lumpur would cost approximately $2.5 million per year to save an estimated seven lives, leading the authors to recommend alternative models for prehospital care. | 3 |
| Henry [60] | 2012 | N/A | A meta-analysis on the effectiveness of prehospital systems using mortality, ISS, and prehospital time as primary and secondary outcomes, showed a 25% decreased risk of dying from trauma in areas that have prehospital trauma systems | Estimates showed a 25% decreased risk of dying from trauma in areas that have prehospital trauma systems. Rural settings showed slightly enhanced treatment effect compared with urban settings (RR rural 0.71 vs. urban 0.79.) | 27 |
| Higashi [61] | 2015 | N/A | A modeling study estimated that 21% of the global injury burden is potentially avertable by essential trauma care. | 21% of the injury burden in LMICs (52.3 million DALYs) could be averted by basic trauma care services. Sub-Saharan Africa had the largest proportion of potentially avertable burden (25%). | 34 |
| Houtchens [62] | 1993 | Armenia, Russia | A telemedicine program between the US and two Armenian and Russian medical centers used in disaster situations positively inﬂuenced patient evaluations and treatment plans. | Telemedicine consultations resulted in altered diagnostic processes and modified treatment plans for 47 of 185 Armenian patients presented. | 30 |
| Husum [63] | 1999 | Afghanistan | Prehospital life support by local paramedics increased the survival rate of war casualties during the battle of Jalalabad, Afghanistan. | 3,890 war casualties were treated in the combat zone by paramedics and evacuated to surgical hospitals in Pakistan, reducing the prehospital mortality rate from 26.% to 13.6%. | 29 |
| Husum [64] | 2003 | Iraq, Cambodia | Training village health workers and laypersons in rural Iraq and Cambodia in prehospital injury care and equipping them with ﬁrst-aid supplies reduced mortality rates. | The mortality rate for trauma victims decreased from 22.6% in 1997 to 13.7% in 1999. Costs for 1 trained paramedic were USD $300 (3 training courses, 450 h) and $800 in equipment (1 ATLS backpack kit), equaling $26 per case. | 28 |
| Husum [65] | 2003 | Iraq, Cambodia | A prehospital care system and training program for nongraduate paramedics and laypersons in rural Iraq and Cambodia reduced mortality. | The trauma mortality rate was reduced from pre-intervention level at 40% to 15% over the study period. | 35 |
| Husum [66] | 2003 | Iraq, Cambodia | In-field ALS provided by local paramedics in mine-infested areas reduced trauma mortality rates. | After implementation of a rural rescue system, there was a reduction in trauma mortality from 26% in 1997 to 12% in 2001. | 31 |
| Jacobs [67] | 2016 | Cambodia | The authors assessed the feasibility of an ambulance service in rural Cambodia. | Ambulance services were well received by the population and authorities with a cost of USD $34 per case. | 18 |
| Jan [68] | 2020 | Pakistan | An assessment of the perception of trainees on ATLS training in Pakistan showed that the course enhanced trainees' approach towards management of emergencies. | 93% of non-surgeon participants and 86% of surgeon participants felt that ATLS should be mandatory. | 6 |
| Jayaraman [69] | 2009 | Uganda | A context-appropriate course on basic first aid improved lay people's knowledge of trauma management in Kampala. | Before training, participants answered 45% of test questions correctly and this increased to 86% after training (p < 0.0001). | 8 |
| Jayaraman [70] | 2009 | Uganda | A one-day basic prehospital trauma care course in Kampala for police, commercial drivers, and community leaders resulted in sustained structural changes at six months | At 6 months, participant knowledge/skill retention remained high, 97% had used at least one skill from the course, and trainees were confident in providing first-aid. The projected cost of scaling this program was $0.12 per capita or $25–75 per life-year saved. | 17 |
| Job [71] | 2011 | Brazil | ATLS training for non-radiologist doctors in Brazil improved their ability to interpret cervical spine and chest radiographs. | Accuracy in cervical spine and chest radiograph interpretation improved by 22% and 26% respectively. | 21 |
| Joshipura [72] | 2006 | India | After using the WHO Guidelines for Essential Trauma Care (GETC), a report from India described sustained structural changes and a stakeholder meeting after facility assessments. | The report delineated strategies to strengthen prehospital and hospital-based care of trauma patients in India and made the GETC the basis for national-level policy changes. | 12 |
| Kannan [73] | 2019 | Uganda | An evaluation of a standardized chart that serves dual functions as clinical documentation and a data collection form in improving data collection rates of key points from the Dataset for Injury (DSI) reported a significant improvement in capturing key elements of the DSI. | The strongest perceived facilitators were motivated local champions and administrative buy-in. The strongest perceived barriers were high staff turnover rate, poor form integration into existing workflow, and lack of equipment to capture quantitative data elements such as vital signs. | 14 |
| Karmacha-rya [74] | 2008 | Nepal | A study presented the planning and organization of prehospital services in Nepal to promote healthcare access to victims in inaccessible regions. | A training package and Emergency Preparedness Workshop were developed, and mobile phone-based information system was proposed. Implementation results remained pending. | 11 |
| Katona [75] | 2015 | South Sudan | A 3-day wilderness first aid training course taught in Kit, South Sudan improved first aid knowledge of community members with limited medical knowledge. | The course resulted in statistically significant improvement in first aid knowledge. Although men started with more health care knowledge, men and women demonstrated equal levels of knowledge upon course completion. | 20 |
| Kesinger [76] | 2014 | Colombia | The introduction of a standardized trauma protocol (STP) for patients with TBI at a level 1 trauma center in Colombia decreased in-hospital mortality, increased discharge GCS, and increased the use of ED interventions (e.g., bladder catheterization, use of hypertonic saline, blood transfusion.) | Post-STP in-hospital mortality decreased (38% vs. 18%), and discharge GCS increased (median 10 vs. 14). Odds of in-hospital mortality post- compared to pre-STP were 0.248. ED interventions increased, including bladder catheterization (49% vs. 73), hypertonic saline (38% vs. 63), arterial blood gas draws (25% vs. 43%), and blood transfusions (3% vs. 18%). | 34 |
| Kesinger [77] | 2014 | Colombia | An STP for damage control resuscitation of trauma patients at a level 1 trauma center in Colombia decreased mortality and length of stay, and signiﬁcantly increased the use of ED interventions. | ED interventions increased pre- and post-STP including blood transfusion (1.0 vs. 2.7 %), tetanus vaccinations (19.3 vs. 26.0 %), adequate IV access (29.5 vs. 34.7 %), prophylactic antibiotics (34.9 vs. 38.0 %.) Mortality of trauma patients decreased (3.9 vs. 2.9 %). | 35 |
| Khan [78] | 2006 | India | A study described the pattern of mine blast limb injuries in a civilian population and evaluated the use of Doppler ultrasound for tissue viability assessments. | The authors conclude that conservative wound debridement to preserve maximum soft tissue results in improved functionally outcomes among landmine victims. | 18 |
| Khan [79] | 2012 | N/A | A systematic review to identify studies reporting rehabilitation outcomes for polytrauma patients identified large evidence gaps. | Major gaps in evidence exist with respect to rehabilitation settings, components, intensity, duration and types of therapy, and long-term outcomes for survivors of polytrauma. | 35 |
| Li [80] | 2007 | China | Implementation of an organized approach to management of severely injured patients at a tertiary hospital in China reported a decrease in mortality and time to operation. | Implementation reduced ISS-adjusted mortality from 39 to 20% and time to operation from 140 to 90 minutes. | 3 |
| Lindquist [81] | 2020 | India | A continuing education course for EMTs in India used low-cost implementation improvements to increase impact. | Strategies included: video instruction for easy translation; low-cost simulation; non-technical skills; training-of-trainers. | 22 |
| Marson [82] | 2001 | Brazil | The development of a prehospital care system in an urban area in Brazil reported reduced early trauma deaths. | Deaths within the first hour after injury were reduced from 54 to 41%. | 6 |
| Mehreen [83] | 2021 | India | An educational intervention on the first aid knowledge of adolescents demonstrated improved test scores. | Pre- and post-intervention knowledge scores of students were 8 and 21, respectively (p<0.001) with an effect size of 3.7. | 32 |
| Merchant [84] | 2015 | Mozambique | A basic trauma resuscitation training in modiﬁed ABCD (airway, breathing, circulation, disability) technique administered to hospital personnel and laypersons in Mozambique reported improved knowledge and skills. | Following the education intervention, both groups demonstrated an improvement in test scores. Hospital personnel and community laypeople had pre- and post-test score of 42% vs 60% and 27% vs 50% respectively. | 8 |
| Mitra [85] | 2020 | India | A prehospital notification application for use by ambulance and emergency clinicians to notify emergency departments of an impending arrival of a patient was associated with improved trauma reception and reduction in early deaths. | More patients were managed with a trauma notification (RR 1.30; 95% CI: 1.10 to 1.52) and trauma team leader presence (RR 1.50; 95% CI: 1.07 to 2.10). There was reduced risk of death in the ED (RR 0.11; 95% CI: 0.03 to 0.39). | 29 |
| Mock [86] | 2003 | US, Ghana, Mexico | A modeling study compared prehospital and hospital-based care in the US, Mexico, and Ghana. The authors suggested that sustained structural changes in essential trauma care may reduce the number of deaths and disabilities. | For both prehospital and hospital-based care, studies revealed several critical weak points to address in: (1) human resources (staffing and training); (2) physical resources (equipment, supplies, and infrastructure); and (3) administration and organization. | 10 |
| Mock [87] | 2012 | US, Ghana, Mexico | A modeling study was conducted based on: (a) case fatality rates for seriously injured persons in the US, Mexico, and Ghana and (b) data from the Global Burden of Disease Study. | Between 1,730,000 and 1,965,000 lives could be saved in LMICs if case fatality rates among seriously injured persons could be reduced to those in HICs, or 34–38% of all injury-related deaths. | 12 |
| Mock [88] | 1993 | US, Ghana | Prehospital and hospital-based care and patient outcomes were compared between a first-level referral hospital in Ghana and a tertiary hospital in the US. | The authors suggested that lack of prehospital care and long delays in transport are partially responsible for significant differences in time to treatment, admission ISS, and mortality rates. | 18 |
| Mock [89] | 1998 | US, Ghana, Mexico | Trauma systems and patient outcomes were compared between cities in the US, Mexico, and Ghana. | Mortality declined with increased economic level, due primarily to decreased prehospital times and prehospital/ED death. | 14 |
| Mock [90] | 2005 | Ghana | A trauma course in rural hospitals in Ghana reported improvements in the knowledge of participants. | Test scores improved from 69% to 80% with improvements in all major categories with high level of utilization of basic airway maneuvers (93%) and chest tube insertion (67%.) | 29 |
| Mock [91] | 2002 | Ghana | A 6-hour ﬁrst-aid course for commercial drivers in Ghana resulted in sustained knowledge and skills improvement. | Improvement in first aid included: scene management (7% pre- vs. 35% post-), airway management (2% vs. 35%), hemorrhage control (4% vs. 42%), and splinting of injured extremities (1 vs. 16%). | 28 |
| Mullan [92] | 2014 | Botswana | Use of a locally-adapted South African Triage Scale in Botswana reduced both over-triage and under-triage rates. | Over-triage improved from 53% to 38% and under-triage from 47% to 16%. ICU admission rates decreased from 0.35 to 0.06%. | 15 |
| Murad [93] | 2010 | Iraq | Prehospital care training for paramedics and laypersons in rural Iraq reduced mortality rates among injured patients who were cared for in the ﬁeld. | The mortality rate was significantly lower among patients initially managed in-field by first responders compared to patients without first-responder support (9.8% versus 15.6%.) | 17 |
| Murad [94] | 2012 | Iraq | Prehospital care training for paramedics and laypersons in Iraq reduced the mortality rate from road trafﬁc injury. | Prehospital care reduced the mortality rate to 8% in the treatment group compared to 44% in the control group. | 23 |
| Murad [95] | 2012 | Iraq | A prehospital care system and training program for medics and laypersons in rural Iraq reported a reduction in mortality. | Trauma mortality was reduced from 17% to 4% with particular survival benefits in more severely injured patients. | 34 |
| Nafissi [96] | 2008 | Iran | A training course for physicians, nurses, EMTs, and laypersons in Iran reported improved physiologic severity scores (PSS) among patients who received care from trained providers. | The physiological status of patients improved significantly by treatment from the emergency clinic to the university hospital with a mean improvement in the PSS of 1.11 (95% CI 0.98–1.24). | 26 |
| Nelson [97] | 2014 | Uganda | An assessment of a training program for non-physician clinicians reported improved patient outcomes and CFR. | The CFR for all trauma patients was 2.9% which the authors felt compared favorably to similar settings. | 5 |
| O'Donnell [98] | 2000 | Somalia | A study after a land mine awareness course to train national staff of NGOs in Somalia reported increased awareness. | The use of simple educational materials and oral dissemination improved awareness for only USD $0.47 per participant. | 11 |
| Olumide [99] | 2015 | Nigeria | An evaluation of first aid training on the knowledge and skills of commercial drivers showed significant improvements. | First aid knowledge scores improved from 49% to 59% pre- and post-course, and skills sores improved from 17.5% to 81%. | 27 |
| Orkin [100] | 2021 | N/A | A systematic review assessed the impact of first aid training for laypeople to build community capacity for emergency health response in low-resource settings. | The quality of evidence is low but overall task-shifting for first aid training of laypersons is a promising approach to reduce morbidity and mortality for numerous health emergencies. | 31 |
| Orlas [101] | 2018 | Colombia | The implementation of the Stop the Bleed course in Colombian improved participant knowledge and willingness to act for hemorrhage control. | Participants were 16 times more likely to obtain a higher score than during the pre-training period and 99% expressed willingness to place a tourniquet on a bleeding victim. | 25 |
| Palomino [102] | 2020 | Colombia | The approach to patients with penetrating thoracic trauma treated with laparoscopy and thoracoscopy in a level 2 trauma center in an LMIC is discussed. | The authors discuss diagnostic and therapeutic benefits and limitation to each approach in a descriptive manner. | 5 |
| Pandey [103] | 2010 | Nepal | A study assessing the management of snake bite victims one year after first aid training reported a decrease in CFR. | The CFR after venomous snake bite decreased to 22% from 27%. | 13 |
| Petroze [104] | 2015 | Rwanda | The implementation of a focused trauma education initiative in Rwanda reported differences in injury-related outcomes and resource utilization. | Mortality of injured patients decreased from 8.8 to 6.3%. Patients with an initial GCS of 3–8 had the highest injury-related mortality, which significantly decreased from 58.5 % to 37.1 %. | 23 |
| Prospero [105] | 2000 | Italy | The authors present a descriptive analysis of the interventions performed for a displaced Yugoslavian population at a first-aid station in Italy. | The authors reported that the resources deployed exceeded actual needs, and gaps existed in inter-agency coordination, data sharing, and follow-up. | 23 |
| Quansah [106] | 2008 | Ghana | A week-long trauma course for doctors in rural districts in Ghana reported an increase in knowledge. | Post-test scores improved for all major sections, and trainees reported performing more trauma procedures after the course. | 21 |
| Richard [107] | 2009 | Myanmar | A Trauma Management Program was developed to improve local health workers’ ability to perform trauma care. | Protocols and procedures taught during training workshops were implemented effectively in the field with over 300 trainees. | 23 |
| Roudsari [108] | 2007 | Multiple | A comparative analysis of pre-hospital trauma care systems reported variation in the use of air transport, physicians as prehospital personnel, and various prehospital interventions. | Air ambulance use was not common except in HICs. Median scene time ranged from 10 min (Mexico) to 30 min (Germany.) Variation in use of IV fluids and prehospital intubation was also observed. | 36 |
| Saghaﬁnia [109] | 2008 | Iran | An animal-model based training course for village healthcare workers reduced mortality among patients with penetrating injuries in Iran. | A significant reduction in mortality was observed pre- and post-training from 7.3% to 3%. | 35 |
| Saghaﬁnia [110] | 2009 | Iran | Three years after the implementation of an education course in Iran, the mean PSS at admission was reported to be higher among those who had received prehospital trauma care. | Mean PSS at admission was higher among those who had received prehospital trauma care (7.5) compared with those who did not (6.8; p < 0.001.) | 4 |
| Saghaﬁnia [111] | 2009 | Iran | A trauma care training program in Iran reported improved PSS among landmine victims who received prehospital care. | Mean PSS among patients who received prehospital care was 7.4 compared to 5.9 among those who did not. | 35 |
| Sangowawa [112] | 2012 | Nigeria | A training for drivers in Nigeria to provide first aid to road crash victims reported improvements in knowledge. | Intervention drivers had a statistically significant increase in skills scores from 49% pre- to 78% post-intervention . | 21 |
| Schuurman [113] | 2011 | South Africa | The Cape Town Trauma Registry (CTTR) was implemented with high rates of data capture. | Successful Implementation of the CTTR was due to the ability to calculate injury severity, key minimal data elements, expansion to include quality indicators, and minimal drain on human resources. | 2 |
| Scott [114] | 2017 | Rwanda | An electronic evaluation of the Rwandan pre-hospital emergency care service reported that a data-driven quality improvement (QI) program yielded immediate and sustained improvements in pre-hospital care for trauma. | All 5 endpoints had a significant increase between the pre- and post-QI periods. The QI program led to an immediate improvement of +6.1% and sustained monthly improvements in care delivery of 0.7% per month. | 25 |
| Sharma [115] | 2013 | Nepal | A layperson ﬁrst-responder and motorcycle prehospital transport system with community health education for snakebite victims in rural Nepal reduced the CFR. | The case-fatality rate was reduced from 11% to <1% post-intervention. | 27 |
| Shehu [116] | 1997 | Nigeria | This intervention focused on informal transport systems to increase health facility accessibility for women with obstetric complications in Nigeria. | The cost of transport ranged from USD $2.10 to $10.40, with a mean cost of $5.89. Time from onset of labor to treatment ranged from 3 to 36 hours, with a mean of 9 hours for the two years. | 28 |
| Son [117] | 2006 | Vietnam | Training for hospital and ambulance staff after an assessment adapted from the WHO GETC resulted in sustained structural changes (e.g., physical and human resources, infrastructure.) | Training improved provider skill and prehospital advances reduced response times from 20 to 15 minutes. | 11 |
| Stanley [118] | 2015 | Thailand, Myanmar | A simple teaching tool to improve patient assessment and management by local health workers with minimal resources reported improved competence and confidence. | Post-test assessment results improved with a median score of 20 and a 71% (42/59) pass rate overall. A significant rise in confidence was observed among participants. | 4 |
| Stevens [119] | 2013 | Kenya | This study examined the challenges and lessons learned from trauma registry implementation in Kenya. | Problems were identified in planning, data collection, entry processes, and analysis. The authors recommended continued engagement and training of local stakeholders. | 9 |
| Stolz [120] | 2015 | Uganda | A point-of-care ultrasound training program in a tertiary hospital in Uganda resulted in sustained structural changes (e.g., retention of trained sonographers.) | The focused assessment with sonography in trauma examination (53%) and echocardiography (16%) were most commonly performed. Point-of-care ultrasound studies were performed more frequently than radiology department-performed studies. Positive findings were documented in 46% of all examinations. | 14 |
| Sun [121] | 2012 | South Africa | A layperson prehospital care system in an urban setting in South Africa resulted in sustained structural changes and improved use of vital prehospital care interventions. | Emergency First Aid Responders (EFARs) tested 28% on competence examinations before vs. 78% after training. EFARs reported using nearly every skill taught. The cost of the course was reported to be USD $6,570 per year or $5 per trainee. | 23 |
| Sun [122] | 2011 | South Africa | A survey assessed Cape Town residents' perception on the impact of the EFAR training course. | The course provided stress relief to the community, increased the likeliness community members will help each other during an emergency, and increased the participant's confidence. | 18 |
| Tannvik [123] | 2012 | N/A | A systematic review examined first aid provided by laypeople to trauma victims and its impact on mortality reduction | Incorrect care was given in up to 84% of cases. Airway and hemorrhage control were areas of concern. One study reported a 6% reduction in mortality whereas two studies estimated that correct first aid could have reduced mortality by 2–4.5%. | 23 |
| Tansley [124] | 2015 | Namibia, Haiti | A modeling study demonstrated poor population-level spatial access to hospital-based trauma care in Namibia and Haiti. | The population of both countries had poor access to services with 25% of the population in Haiti and 51% in Namibia living >50km from a facility capable of providing 24-hour care. | 21 |
| Tiska [125] | 2004 | Ghana | Trauma training for commercial drivers in Ghana found that the greatest difference in prehospital mortality could be made through airway management and hemorrhage control. | Hemorrhage control was quickly learned and used appropriately by the drivers. The course cost approximately USD $3 per student. | 6 |
| Tolppa [126] | 2020 | Congo | A two-day Primary Trauma Care course delivered to healthcare workers in the Democratic Republic of Congo over two years reported sustained improvements in knowledge. | There was an increase of 4.8 in test scores and 9.6 in confidence scores (p < 0.01) post-course. | 35 |
| Ullrich [127] | 2021 | Uganda | A pediatric emergency surgical care course for non-specialist rural providers was found to improve knowledge. | Scores on the knowledge tests improved significantly from 55% to 72% post-course p <0.0001. | 4 |
| Vakili [128] | 2014 | Iran | A first aid training course for drivers from northern Iran demonstrated improvements in first aid administration. | Improvements were observed in drivers' efficacy in first aid (hemorrhage control, airway, fracture immobilization). The rate of correct interventions was higher at 4-6 months than at 0-3 months. | 21 |
| Van de Velde [129] | 2011 | N/A | Evidence-based guidelines on first aid interventions requiring minimal or no equipment for training first responders in African were produced. | The implementation of newly developed guidelines through Red Cross national societies across Africa was planned. No quantitative outcomes were reported. | 11 |
| Van Heng [130] | 2008 | Cambodia | A surgical skills training program for nondoctors in Cambodia demonstrated no effect on the already-low in-hospital trauma mortality rate but improved postoperative infection and provider coping capacity. | The in-hospital trauma fatality rate was low in both populations and not significantly affected by the intervention. Post-operative infections were reduced from 22.0% to 10.3% and trainees’ self-rated skill and coping improved. | 18 |
| VanRooyen [131] | 2000 | South Sudan | A training program to teach civilian prehospital care to military medics in the Sudan People’s Liberation Army improved knowledge among trainees. | A mean improvement in pre- and post- test scores of 35.0% (p<0.0005) was improved, with the most dramatic improvements in wound care, hemorrhage, and head trauma. | 23 |
| Wafik [132] | 2014 | Egypt | A study examining the effectiveness of a first-aid program delivered by undergraduate nursing students to preparatory school children in Egypt reported statistically significant improvements knowledge and practice. | Statistically significant improvements in post-test scores were shown in all areas. Only 1% of school students had satisfactory knowledge at the pretest phase, compared to 100% of school students in the post- and follow-up phases (P < 0.001). | 5 |
| Wang [133] | 2010 | China | Implementation of ATLS training decreased mortality and the duration in the ED from admission to operation. | Mortality decreased from 20% to 15% post-ATLS implementation, with a reduction in time to operation of 61.5 vs. 48 minutes. | 20 |
| Wesson [134] | 2014 | N/A | A literature review focusing on injury-related costing studies in LMICs reported a wide variation in cost of injury. | Cost per DALY averted for injury-prevention interventions ranged widely from USD $10.9 for speed bump installation to $17,000 for drunk driving and breath testing campaigns in Africa. | 33 |
| Wisborg [135] | 2008 | Iraq | An assessment of villagers' perception of the impact of first responder training found that the systems’ success was due to a strong local anchor and adaptation to population needs. | Paramedics were perceived as having a large impact as an emergency medical resource. The program coordinator was a villager himself, who facilitated adaptation to local needs. | 24 |
| Wisborg [136] | 2008 | Iraq | Prehospital care training for paramedics and laypersons in rural Iraq resulted in reduced time from injury to treatment, and improved admission PSS. | Mortality in victims of mines and war injuries decreased from 28% to 9.4%, as did time from injury to treatment (2.4 to 0.6 hours.) Retention of paramedics in the program was 72% after 8 years. | 31 |
| Wu [137] | 2016 | N/A | A 2-day training course delivered to orthopedic surgeons from LMICs reported improved management of soft tissue defects and reduced rate of amputations. | Participants rated the training at ≥4.4 on a Likert scale of 5. 100% of participants performed flaps learned at the course to treat soft-tissue defects, preventing 116 patients from undergoing amputation. 97% of course participants taught flap reconstruction techniques to others. | 17 |
| Xu [138] | 2011 | China | A retrospective review of victims with earthquake-related head injuries admitted to the Department of Neurosurgery or Neurosurgical ICU was conducted. | Tertiary hospitals with neurosurgeons and advanced equipment did not effectively contribute to successful treatment. Mobile hospitals played an important role in initial triage and treatment. | 6 |

*QUADS scoring framework: 0-6 = Very Low; 7-13 = Low; 14-26 = Moderate; 27-33 = High; 34-39 = Very High.[139]

Abbreviations: *ACS-COT –* American College of Surgeons Committee on Trauma; *ALS –* Advanced Life Support*; ATLS –* Advanced Trauma Life Support; *BTLS* – Basic Trauma Life Support; *CFR –* Case Fatality Rate; *CPR* -- Cardiopulmonary Resuscitation; *CTTR –* Cape Town Trauma Registry*; DALY –* Disability-Adjusted Life Years *; DATC –* Definitive Anesthesia Trauma Care Course*; DRC –* Democratic Republic of Congo*; DSI –* Dataset for Injury; *DSTC –* Definitive Surgical Trauma Care Course*; ED –* Emergency Department; *EFAR –* Emergency First Aid Responders; *EMS –* Emergency Medical Services; *EMT –* Emergency Medical Technician*; EMWT –* Emergency Ward Management of Trauma; *GCS –* Glasgow Coma Scale; *GETC –* Guidelines for Essential Trauma Care; *IQR –* Interquartile range*; ISS –* Injury Severity Score; *HIC –* High-income Country*; HIV –* Human Immunodeficiency Virus; *ICU –* Intensive care unit; *IV –* Intravenous; *LMIC* – Low- and middle-income country; *MSF –* Médecins sans Frontières; *MVC –* Motor Vehicle Crash; *NGO –* Nongovernmental Organization; *OR –* Odds ratio; *PHPTC –* Prehospital Pediatric Trauma Classification*; PHTLS –* Prehospital Trauma Life Support*; PSS –* Physiologic Severity Score; *QI –* Quality improvement; *RR –* Relative risk*; RRTT –* Rapid Response Trauma Team*; RSI –* Rapid Sequence Intubation*; STaRTLE –* Surgical Techniques and Repairs in Trauma for the Low-resource Environment; *STP –* Standardized Trauma Protocol; *TFRC –* Trauma First Responder Course; *US* -- United States; *USD –* United States Dollar; *WHO –* World Health Organization

**References**

1. Abib S de CV, Schettini ST, Figueiredo LFP de (2006) Prehospital pediatric trauma classification (PHPTC) as a tool for optimizing trauma care resources in the city of São Paulo, Brazil. Acta Cir Bras 21:7–11. https://doi.org/10.1590/s0102-86502006000100003

2. Aboutanos MB, Rodas EB, Aboutanos SZ, et al (2007) Trauma education and care in the jungle of Ecuador, where there is no advanced trauma life support. J Trauma 62:714–719. https://doi.org/10.1097/TA.0b013e318031b56d

3. Abraham R, Vyas D, Narayan M, Vyas A (2015) Strategically Leapfrogging Education in Prehospital Trauma Management: Four-Tiered Training Protocols. Am J Robot Surg 2:9–15. https://doi.org/10.1166/ajrs.2015.1022

4. Adolfi Júnior MS, Pallini FM, Pessotti H, et al (2010) Emergency medical coordination using a web platform: a pilot study. Rev Saude Publica 44:1063–1071. https://doi.org/10.1590/s0034-89102010000600011

5. Aekka A, Abraham R, Hollis M, et al (2015) Prehospital trauma care education for first responders in India. J Surg Res 197:331–338. https://doi.org/10.1016/j.jss.2015.03.047

6. Agrawal D (2012) Transforming trauma healthcare delivery in rural areas by use of an integrated call center. J Emerg Trauma Shock 5:7–10. https://doi.org/10.4103/0974-2700.93099

7. Alexandrino H, Baptista S, Vale L, et al (2020) Improving Intraoperative Communication in Trauma: The Educational Effect of the Joint DSTC^TM^-DATC^TM^ Courses. World J Surg 44:1856–1862. https://doi.org/10.1007/s00268-020-05421-5

8. Ali J, Adam R, Butler AK, et al (1993) Trauma outcome improves following the advanced trauma life support program in a developing country. J Trauma 34:890–898; discussion 898-899. https://doi.org/10.1097/00005373-199306000-00022

9. Ali J, Adam RU, Gana TJ, et al (1997) Effect of the prehospital trauma life support program (PHTLS) on prehospital trauma care. J Trauma 42:786–790. https://doi.org/10.1097/00005373-199705000-00006

10. Ali J, Adam R, Stedman M, et al (1994) Advanced trauma life support program increases emergency room application of trauma resuscitative procedures in a developing country. J Trauma 36:391–394. https://doi.org/10.1097/00005373-199403000-00020

11. Alizadeh R, Panahi F, Saghafinia M, et al (2012) Impact of trauma dispatch algorithm software on the rate of missions of emergency medical services. Trauma Mon 17:319–322. https://doi.org/10.5812/traumamon.6341

12. AlSabah S, Al Haddad E, AlSaleh F (2018) Stop the bleed campaign: A qualitative study from our experience from the middle east. Ann Med Surg (Lond) 36:67–70. https://doi.org/10.1016/j.amsu.2018.10.013

13. Sarmiento Altamirano D, Himmler A, Chango Sigüenza O, et al (2020) The Successful Implementation of a Trauma and Acute Care Surgery Model in Ecuador. World J Surg 44:1736–1744. https://doi.org/10.1007/s00268-020-05435-z

14. Altintaş KH, Bilir N, Tüleylioğlu M (1999) Costing of an ambulance system in a developing country, Turkey: costs of Ankara Emergency Aid and Rescue Services’ (EARS) ambulance system. Eur J Emerg Med 6:355–362. https://doi.org/10.1097/00063110-199912000-00014

15. Alto L de SM (2008) Análise do impacto do Programa ATLS (R) (Advanced Trauma Life Support (R)) no atendimento do traumatizado em cidade de pequeno porte no Brasil. Text, Universidade de São Paulo

16. Amiri H, Gholipour C, Mokhtarpour M, et al (2013) Two-day primary trauma care workshop: early and late evaluation of knowledge and practice. Eur J Emerg Med 20:130–132. https://doi.org/10.1097/MEJ.0b013e32835608c6

17. Anderson GA, Kayima P, Ilcisin L, et al (2018) Development of a Comprehensive Trauma Training Curriculum for the Resource-Limited Environment. J Surg Educ 75:1317–1324. https://doi.org/10.1016/j.jsurg.2018.02.014

18. Arnson Y, Bar-Dayan Y (2009) Reducing landmine mortality rates in Iran using public medical education and rural rescue teams--what can be learned from landmine casualties, and how can the situation be improved? Prehosp Disaster Med 24:130–132. https://doi.org/10.1017/s1049023x00006683

19. Arreola-Risa C, Mock C, Herrera-Escamilla AJ, et al (2004) Cost-effectiveness and benefit of alternatives to improve training for prehospital trauma care in Mexico. Prehosp Disaster Med 19:318–325. https://doi.org/10.1017/s1049023x00001953

20. Arreola-Risa C, Mock CN, Lojero-Wheatly L, et al (2000) Low-cost improvements in prehospital trauma care in a Latin American city. J Trauma 48:119–124. https://doi.org/10.1097/00005373-200001000-00020

21. Arreola-Risa C, Mock CN, Padilla D, et al (1995) Trauma care systems in urban Latin America: the priorities should be prehospital and emergency room management. J Trauma 39:457–462. https://doi.org/10.1097/00005373-199509000-00011

22. Arreola-Risa C, Vargas J, Contreras I, Mock C (2007) Effect of emergency medical technician certification for all prehospital personnel in a Latin American city. J Trauma 63:914–919. https://doi.org/10.1097/TA.0b013e31806bf141

23. Balhara KS, Bustamante ND, Selvam A, et al (2019) Bystander Assistance for Trauma Victims in Low- and Middle-Income Countries: A Systematic Review of Prevalence and Training Interventions. Prehosp Emerg Care 23:389–410. https://doi.org/10.1080/10903127.2018.1513104

24. Bandyopadhyay L, Manjula M, Paul B, Dasgupta A (2017) Effectiveness of first-aid training on school students in Singur Block of Hooghly District, West Bengal. J Family Med Prim Care 6:39–42. https://doi.org/10.4103/2249-4863.214960

25. Barss P, Blackford C (1983) Medical emergency flights in remote areas: experience in Milne Bay Province, Papua New Guinea. P N G Med J 26:198–202

26. Barthel ER, Pierce JR, Goodhue CJ, et al (2011) Availability of a pediatric trauma center in a disaster surge decreases triage time of the pediatric surge population: a population kinetics model. Theor Biol Med Model 8:38. https://doi.org/10.1186/1742-4682-8-38

27. Bertol MJ, Van den Bergh R, Trelles Centurion M, et al (2014) Saving life and limb: limb salvage using external fixation, a multi-centre review of orthopaedic surgical activities in Médecins Sans Frontières. Int Orthop 38:1555–1561. https://doi.org/10.1007/s00264-014-2451-6

28. Bissell RA, Pinet L, Nelson M, Levy M (2004) Evidence of the effectiveness of health sector preparedness in disaster response: the example of four earthquakes. Fam Community Health 27:193–203. https://doi.org/10.1097/00003727-200407000-00006

29. Boeck MA, Callese TE, Nelson SK, et al (2018) The development and implementation of a layperson trauma first responder course in La Paz, Bolivia: A pilot study. Injury 49:885–896. https://doi.org/10.1016/j.injury.2017.11.022

30. Boeck MA, Schuetz SJ, Miller CG, et al (2015) A novel trauma first responder course in Potosí, Bolivia: initial results. Annals of Global Health 81:17. https://doi.org/10.1016/j.aogh.2015.02.552

31. Brooks AJ, Phipson M, Potgieter A, et al (1999) Education of the trauma team: video evaluation of the compliance with universal barrier precautions in resuscitation. Eur J Surg 165:1125–1128. https://doi.org/10.1080/110241599750007621

32. Brown HA, Douglass KA, Ejas S, Poovathumparambil V (2016) Development and Implementation of a Novel Prehospital Care System in the State of Kerala, India. Prehosp Disaster Med 31:663–666. https://doi.org/10.1017/S1049023X16000960

33. Bruijns SR, Wallis LA, Burch VC (2008) A prospective evaluation of the Cape triage score in the emergency department of an urban public hospital in South Africa. Emerg Med J 25:398–402. https://doi.org/10.1136/emj.2007.051177

34. Buntman AJ, Yeomans KA (2002) The effect of air medical transport on survival after trauma in Johannesburg, South Africa. S Afr Med J 92:807–811

35. Cai B, Sigrid B, Redick B, et al (2014) Comprehensive level one trauma center could lower in-hospital mortality of severe trauma in China. Biomed Environ Sci 27:537–543. https://doi.org/10.3967/bes2014.084

36. Calderale SM, Sandru R, Tugnoli G, et al (2008) Comparison of quality control for trauma management between Western and Eastern European trauma center. World J Emerg Surg 3:32. https://doi.org/10.1186/1749-7922-3-32

37. Callese TE, Richards CT, Shaw P, et al (2014) Layperson trauma training in low- and middle-income countries: a review. J Surg Res 190:104–110. https://doi.org/10.1016/j.jss.2014.03.029

38. Capone PL, Lane JC, Kerr CS, Safar P (2000) Life supporting first aid (LSFA) teaching to Brazilians by television spots. Resuscitation 47:259–265. https://doi.org/10.1016/s0300-9572(00)00230-6

39. Carlson LC, Slobogean GP, Pollak AN (2012) Orthopaedic trauma care in Haiti: a cost-effectiveness analysis of an innovative surgical residency program. Value Health 15:887–893. https://doi.org/10.1016/j.jval.2012.06.004

40. lecture1-2-advancedemergencytraumacourse-introductionandcourseoverview_2.pdf

41. Chaleekrua T, Phlainoi S, Jirojanakul P, Plitponkarnpim A (2011) Development of a Community-based Pre-hospital Care Management Model for Emergency Volunteers. 56:287–296

42. Chittawatanarat K, Ditsatham C, Chandacham K, Chotirosniramit N (2013) Effects of rapid response trauma team in thoracic injuries in northern trauma center level I. J Med Assoc Thai 96:1319–1325

43. Colohan AR, Alves WM, Gross CR, et al (1989) Head injury mortality in two centers with different emergency medical services and intensive care. J Neurosurg 71:202–207. https://doi.org/10.3171/jns.1989.71.2.0202

44. Ss de R, J D, S C, et al (2014) Emergency response in resource-poor settings: a review of a newly-implemented EMS system in rural Uganda. Prehospital and disaster medicine 29:. https://doi.org/10.1017/S1049023X14000363

45. D D, M M, A S, et al (2005) The effect of trauma center designation and trauma volume on outcome in specific severe injuries. Annals of surgery 242:. https://doi.org/10.1097/01.sla.0000184169.73614.09

46. J D, S G, B S (2005) Effective mine risk education in war-zone areas--a shared responsibility. Health promotion international 20:. https://doi.org/10.1093/heapro/dai014

47. Duron V, DeUgarte D, Bliss D, et al (2016) Implementation and analysis of initial trauma registry in Iquitos, Peru. Health Promot Perspect 6:174–179. https://doi.org/10.15171/hpp.2016.28

48. El-Shinawi M, McCunn M, Sisley AC, et al (2015) Developing sustainable trauma care education in Egypt: sequential trauma education program, steps to success. J Surg Educ 72:e29-32. https://doi.org/10.1016/j.jsurg.2014.12.001

49. Erickson TB, VanRooyen MJ, Werbiski P, et al (1996) Emergency medicine education intervention in Rwanda. Ann Emerg Med 28:648–651. https://doi.org/10.1016/s0196-0644(96)70088-4

50. Fuzaylov G, Dabek RJ, Bojovic B, et al (2021) Experience of outreach in a resource-constrained environment: 10 years of outcomes in burn care. Burns 47:1656–1664. https://doi.org/10.1016/j.burns.2021.01.019

51. Gallaher JR, Banda W, Robinson B, et al (2020) Access to Operative Intervention Reduces Mortality in Adult Burn Patients in a Resource-Limited Setting in Sub-Saharan Africa. World J Surg 44:3629–3635. https://doi.org/10.1007/s00268-020-05684-y

52. Geduld H, Wallis L (2011) Taxi driver training in Madagascar: the first step in developing a functioning prehospital emergency care system. Emerg Med J 28:794–796. https://doi.org/10.1136/emj.2010.101683

53. Georgoff P, Meghan S, Mirza K, Stein SC (2010) Geographic variation in outcomes from severe traumatic brain injury. World Neurosurg 74:331–345. https://doi.org/10.1016/j.wneu.2010.03.025

54. Gosselin RA, Heitto M (2008) Cost-effectiveness of a district trauma hospital in Battambang, Cambodia. World J Surg 32:2450–2453. https://doi.org/10.1007/s00268-008-9708-4

55. Gosselin RA, Maldonado A, Elder G (2010) Comparative cost-effectiveness analysis of two MSF surgical trauma centers. World J Surg 34:415–419. https://doi.org/10.1007/s00268-009-0230-0

56. Hanche-Olsen TP, Alemu L, Viste A, et al (2015) Evaluation of training program for surgical trauma teams in Botswana. World J Surg 39:658–668. https://doi.org/10.1007/s00268-014-2873-8

57. Hashmi ZG, Haider AH, Zafar SN, et al (2013) Hospital-based trauma quality improvement initiatives: first step toward improving trauma outcomes in the developing world. J Trauma Acute Care Surg 75:60–68; discussion 68. https://doi.org/10.1097/TA.0b013e31829880a0

58. Hauswald M, Ong G, Tandberg D, Omar Z (1998) Out-of-hospital spinal immobilization: its effect on neurologic injury. Acad Emerg Med 5:214–219. https://doi.org/10.1111/j.1553-2712.1998.tb02615.x

59. Hauswald M, Yeoh E (1997) Designing a prehospital system for a developing country: estimated cost and benefits. Am J Emerg Med 15:600–603. https://doi.org/10.1016/s0735-6757(97)90167-4

60. Henry JA, Reingold AL (2012) Prehospital trauma systems reduce mortality in developing countries: a systematic review and meta-analysis. J Trauma Acute Care Surg 73:261–268. https://doi.org/10.1097/TA.0b013e31824bde1e

61. Higashi H, Barendregt JJ, Kassebaum NJ, et al (2015) Burden of injuries avertable by a basic surgical package in low- and middle-income regions: a systematic analysis from the Global Burden of Disease 2010 Study. World J Surg 39:1–9. https://doi.org/10.1007/s00268-014-2685-x

62. Houtchens BA, Clemmer TP, Holloway HC, et al (1993) Telemedicine and international disaster response. Medical consultation to Armenia and Russia via a Telemedicine Spacebridge. Prehosp Disaster Med 8:57–66

63. Husum H (1999) Effects of early prehospital life support to war injured: the battle of Jalalabad, Afghanistan. Prehosp Disaster Med 14:75–80

64. Husum H, Gilbert M, Wisborg T (2003) Training pre-hospital trauma care in low-income countries: the “Village University” experience. Med Teach 25:142–148. https://doi.org/10.1080/0142159031000092526

65. Husum H, Gilbert M, Wisborg T, et al (2003) Rural Prehospital Trauma Systems Improve Trauma Outcome in Low-Income Countries: A Prospective Study from North Iraq and Cambodia. Journal of Trauma and Acute Care Surgery 54:1188. https://doi.org/10.1097/01.TA.0000073609.12530.19

66. Husum H, Gilbert M, Wisborg T, et al (2003) Land mine injuries: a study of 708 victims in North Iraq and Cambodia. Mil Med 168:934–940

67. Jacobs B, Men C, Sam OS, Postma S (2016) Ambulance services as part of the district health system in low-income countries: a feasibility study from Cambodia. Int J Health Plann Manage 31:414–429. https://doi.org/10.1002/hpm.2285

68. (2020) ASSESSMENT OF ADVANCED TRAUMA LIFE SUPPORT COURSE AMONG TRAINEES AS KEY TREATMENT OBJECTIVES: A CASE OF CPSP REGIONAL CENTRE PESHAWAR, PAKISTAN | Journal of Medical Sciences

69. Jayaraman S, Mabweijano JR, Lipnick MS, et al (2009) Current patterns of prehospital trauma care in Kampala, Uganda and the feasibility of a lay-first-responder training program. World J Surg 33:2512–2521. https://doi.org/10.1007/s00268-009-0180-6

70. Jayaraman S, Mabweijano JR, Lipnick MS, et al (2009) First things first: effectiveness and scalability of a basic prehospital trauma care program for lay first-responders in Kampala, Uganda. PLoS One 4:e6955. https://doi.org/10.1371/journal.pone.0006955

71. Job PM, Von Bahten LC, de Oliveira-Junior N (2011) Evaluation of the effectiveness of systematized training of advanced trauma life support protocol in the interpretation of cervical spine and chest radiographs in three different emergency services. J Trauma 70:E122-124. https://doi.org/10.1097/TA.0b013e3181bbd721

72. Joshipura M (2006) Guidelines for essential trauma care: progress in India. World J Surg 30:930–933. https://doi.org/10.1007/s00268-005-0765-7

73. Kannan VC, Kalanzi J, Osiro M, Reynolds T (2019) 198EMF Improving Ugandan Trauma Data Capture and Documentation Quality Using A Novel World Health Organization Emergency Unit Trauma Form. Annals of Emergency Medicine 74:S78. https://doi.org/10.1016/j.annemergmed.2019.08.204

74. Nepal Community Emergency Preparedness Group, Karmacharya PC, Singh GK, et al (2008) Managing the injury burden in Nepal. Clin Orthop Relat Res 466:2343–2349. https://doi.org/10.1007/s11999-008-0384-3

75. Katona LB, Douglas WS, Lena SR, et al (2015) Wilderness First Aid Training as a Tool for Improving Basic Medical Knowledge in South Sudan. Prehosp Disaster Med 30:574–578. https://doi.org/10.1017/S1049023X15005270

76. Kesinger MR, Nagy LR, Sequeira DJ, et al (2014) A standardized trauma care protocol decreased in-hospital mortality of patients with severe traumatic brain injury at a teaching hospital in a middle-income country. Injury 45:1350–1354. https://doi.org/10.1016/j.injury.2014.04.037

77. Kesinger MR, Puyana JC, Rubiano AM (2014) Improving trauma care in low- and middle-income countries by implementing a standardized trauma protocol. World J Surg 38:1869–1874. https://doi.org/10.1007/s00268-014-2534-y

78. Khan MI, Zafar A, Khan N, et al (2006) Outcome of tissue sparing surgical intervention in mine blast limb injuries. J Coll Physicians Surg Pak 16:773–776

79. Khan F, Amatya B, Hoffman K (2012) Systematic review of multidisciplinary rehabilitation in patients with multiple trauma. Br J Surg 99 Suppl 1:88–96. https://doi.org/10.1002/bjs.7776

80. Li N, Fang W, Gu Y, et al (2007) First aid strategy for severe traumatic patients in hospital. Chin J Traumatol 10:357–359

81. Lindquist BD, Koval KW, Acker PC, et al (2020) Continuing Education for Prehospital Healthcare Providers in India - A Novel Course and Concept. Open Access Emerg Med 12:201–210. https://doi.org/10.2147/OAEM.S249447

82. Marson AC, Thomson JC (2001) The influence of prehospital trauma care on motor vehicle crash mortality. J Trauma 50:917–920; discussion 920-921. https://doi.org/10.1097/00005373-200105000-00024

83. Mehreen S, Mathur A, Jat J, Pathak A (2021) Effectiveness of an Educational School-Based Intervention on Knowledge of Unintentional Injury Prevention and First Aid Among Students in Ujjain, India. Indian Pediatr 58:532–536

84. Merchant A, Outhay M, Gonzaléz-Calvo L, et al (2015) Training laypersons and hospital personnel in basic resuscitation techniques: an approach to impact the global trauma burden in Mozambique. World J Surg 39:1433–1437. https://doi.org/10.1007/s00268-015-2966-z

85. Mitra B, Kumar V, O’Reilly G, et al (2020) Prehospital notification of injured patients presenting to a trauma centre in India: a prospective cohort study. BMJ Open 10:e033236. https://doi.org/10.1136/bmjopen-2019-033236

86. Mock C, Arreola-Risa C, Quansah R (2003) Strengthening care for injured persons in less developed countries: a case study of Ghana and Mexico. Inj Control Saf Promot 10:45–51. https://doi.org/10.1076/icsp.10.1.45.14114

87. Mock C, Joshipura M, Arreola-Risa C, Quansah R (2012) An estimate of the number of lives that could be saved through improvements in trauma care globally. World J Surg 36:959–963. https://doi.org/10.1007/s00268-012-1459-6

88. Mock CN, Adzotor KE, Conklin E, et al (1993) Trauma outcomes in the rural developing world: comparison with an urban level I trauma center. J Trauma 35:518–523. https://doi.org/10.1097/00005373-199310000-00004

89. Mock CN, Jurkovich GJ, nii-Amon-Kotei D, et al (1998) Trauma mortality patterns in three nations at different economic levels: implications for global trauma system development. J Trauma 44:804–812; discussion 812-814. https://doi.org/10.1097/00005373-199805000-00011

90. Mock CN, Quansah R, Addae-Mensah L, Donkor P (2005) The development of continuing education for trauma care in an African nation. Injury 36:725–732. https://doi.org/10.1016/j.injury.2004.12.044

91. Mock CN, Tiska M, Adu-Ampofo M, Boakye G (2002) Improvements in prehospital trauma care in an African country with no formal emergency medical services. J Trauma 53:90–97. https://doi.org/10.1097/00005373-200207000-00018

92. Mullan PC, Torrey SB, Chandra A, et al (2014) Reduced overtriage and undertriage with a new triage system in an urban accident and emergency department in Botswana: a cohort study. Emerg Med J 31:356–360. https://doi.org/10.1136/emermed-2012-201900

93. Murad MK, Husum H (2010) Trained lay first responders reduce trauma mortality: a controlled study of rural trauma in Iraq. Prehosp Disaster Med 25:533–539. https://doi.org/10.1017/s1049023x00008724

94. Murad MK, Issa DB, Mustafa FM, et al (2012) Prehospital trauma system reduces mortality in severe trauma: a controlled study of road traffic casualties in Iraq. Prehosp Disaster Med 27:36–41. https://doi.org/10.1017/S1049023X11006819

95. Murad MK, Larsen S, Husum H (2012) Prehospital trauma care reduces mortality. Ten-year results from a time-cohort and trauma audit study in Iraq. Scand J Trauma Resusc Emerg Med 20:13. https://doi.org/10.1186/1757-7241-20-13

96. Nafissi N, Saghafinia M, Balochi K (2008) Improving trauma care in rural Iran by training existing treatment chains. Rural Remote Health 8:881

97. Bisanzo M (2014) Training emergency care practitioners and creating access to acute care services in Uganda: The pilot phase. 80:172. https://doi.org/10.1016/j.aogh.2014.08.044

98. O’Donnell CR (2000) A land mine awareness program in Southern Somalia. Journal of Multicultural Nursing & Health 6:42

99. Olumide AO, Asuzu MC, Kale OO (2015) Effect of First Aid Education on First Aid Knowledge and Skills of Commercial Drivers in South West Nigeria. Prehosp Disaster Med 30:579–585. https://doi.org/10.1017/S1049023X15005282

100. Orkin AM, Venugopal J, Curran JD, et al (2021) Emergency care with lay responders in underserved populations: a systematic review. Bull World Health Organ 99:514-528H. https://doi.org/10.2471/BLT.20.270249

101. Orlas CP, Angamarca E, Manzano-Nunez R, et al (2018) CONTROL PREHOSPITALARIO DE LA HEMORRAGIA EN PACIENTES DE TRAUMA. In: XX Simposio de investigaciones en salud “Enfermedades Crónicas No Transmisibles”

102. Palomino WM, Pendelton A, Ferrada R, Ferrada P (2020) Safety and Feasibility of Minimally Invasive Approach for Trauma in a Low-Resource Income Environment. The American Surgeon^TM^ 86:524–526. https://doi.org/10.1177/0003134820919742

103. Pandey DP, Thapa CL, Hamal PK (2010) Impact of first aid training in management of snake bite victims in Madi valley. J Nepal Health Res Counc 8:5–9

104. Petroze RT, Byiringiro JC, Ntakiyiruta G, et al (2015) Can Focused Trauma Education Initiatives Reduce Mortality or Improve Resource Utilization in a Low-Resource Setting? World J Surg 39:926–933. https://doi.org/10.1007/s00268-014-2899-y

105. Prospero E, Raffo M, Appignanesi R, et al (2000) Evaluation of a transit first-aid station providing emergency care to former Yugoslavian war victims evacuated in Ancona, Italy. Eur J Epidemiol 16:253–256. https://doi.org/10.1023/a:1007670201028

106. Quansah R, Abantanga F, Donkor P (2008) Trauma Training for Nonorthopaedic Doctors in Low- and Middle-income Countries. Clin Orthop Relat Res 466:2403–2412. https://doi.org/10.1007/s11999-008-0401-6

107. Richard AJ, Lee CI, Richard MG, et al (2009) Essential trauma management training: addressing service delivery needs in active conflict zones in eastern Myanmar. Human Resources for Health 7:19. https://doi.org/10.1186/1478-4491-7-19

108. Roudsari BS, Nathens AB, Arreola-Risa C, et al (2007) Emergency Medical Service (EMS) systems in developed and developing countries. Injury 38:1001–1013. https://doi.org/10.1016/j.injury.2007.04.008

109. Nia MS, Naffisi N, Mohebbi HA, Moharamzadeh Y (2008) The role of performing life support courses in rural areas in improving pre-hospital physiologic conditions of patients with penetrating injuries. J Coll Physicians Surg Pak 18:538–41

110. M. Saghafi N, N. N, S. M, et al (2008) [Assessment of the role of prevention training and care in trauma patients in rural regions 2001-2005]. 83–90

111. Saghafinia M, Nafissi N, Asadollahi R (2009) Effect of the rural rescue system on reducing the mortality rate of landmine victims: a prospective study in Ilam Province, Iran. Prehosp Disaster Med 24:126–129

112. Sangowawa AO, Owoaje ET (2012) Building capacity of drivers in Nigeria to provide first aid for road crash victims. Injury Prevention 18:62–65. https://doi.org/10.1136/injuryprev-2011-040134

113. Schuurman N, Cinnamon J, Matzopoulos R, et al (2011) Collecting injury surveillance data in low- and middle-income countries: The Cape Town Trauma Registry pilot. Global Public Health 6:874–889. https://doi.org/10.1080/17441692.2010.516268

114. Scott JW, Nyinawankusi JD, Enumah S, et al (2017) Improving prehospital trauma care in Rwanda through continuous quality improvement: an interrupted time series analysis. Injury 48:1376–1381. https://doi.org/10.1016/j.injury.2017.03.050

115. Sharma SK, Bovier P, Jha N, et al (2013) Effectiveness of Rapid Transport of Victims and Community Health Education on Snake Bite Fatalities in Rural Nepal. Am J Trop Med Hyg 89:145–150. https://doi.org/10.4269/ajtmh.12-0750

116. Shehu D, Ikeh A t, Kuna M j, Team) (The Sokoto PMM (1997) Mobilizing transport for obstetric emergencies in northwestern Nigeria. International Journal of Gynecology & Obstetrics 59:S173–S180. https://doi.org/10.1016/S0020-7292(97)00163-X

117. Son NT, Mock C (2006) Improvements in trauma care capabilities in Vietnam through use of the WHO-IATSIC Guidelines for Essential Trauma Care. International Journal of Injury Control and Safety Promotion 13:125–127. https://doi.org/10.1080/17457300500310152

118. Stanley L, Min TH, Than HH, et al (2015) A tool to improve competence in the management of emergency patients by rural clinic health workers: a pilot assessment on the Thai-Myanmar border. Confl Health 9:11. https://doi.org/10.1186/s13031-015-0041-x

119. Stevens KA, Paruk F, Bachani AM, et al (2013) Establishing hospital-based trauma registry systems: lessons from Kenya. Injury 44:S70–S74. https://doi.org/10.1016/S0020-1383(13)70216-X

120. Stolz LA, Muruganandan KM, Bisanzo MC, et al (2015) Point-of-care ultrasound education for non-physician clinicians in a resource-limited emergency department. Tropical Medicine & International Health 20:1067–1072. https://doi.org/10.1111/tmi.12511

121. Sun JH, Wallis LA (2012) The emergency first aid responder system model: using community members to assist life-threatening emergencies in violent, developing areas of need. Emerg Med J 29:673–678. https://doi.org/10.1136/emermed-2011-200271

122. Sun JH, Wallis LA (2011) The psychological effects of widespread emergencies and a first responder training course on a violent, developing community. African Journal of Emergency Medicine 1:166–173. https://doi.org/10.1016/j.afjem.2011.10.007

123. Tannvik TD, Bakke HK, Wisborg T (2012) A systematic literature review on first aid provided by laypeople to trauma victims. Acta Anaesthesiol Scand 56:1222–1227. https://doi.org/10.1111/j.1399-6576.2012.02739.x

124. Tansley G, Schuurman N, Amram O, Yanchar N (2015) Spatial Access to Emergency Services in Low- and Middle-Income Countries: A GIS-Based Analysis. PLoS One 10:e0141113. https://doi.org/10.1371/journal.pone.0141113

125. Tiska M, Adu-Ampofo M, Boakye G, et al (2004) A model of prehospital trauma training for lay persons devised in Africa. Emerg Med J 21:237–239. https://doi.org/10.1136/emj.2002.002097

126. Tolppa T, Vangu AM, Balu HC, et al (2020) Impact of the primary trauma care course in the Kongo Central province of the Democratic Republic of Congo over two years. Injury 51:235–242. https://doi.org/10.1016/j.injury.2019.12.013

127. Ullrich S, Kisa P, Ruzgar N, et al (2021) Implementation of a contextually appropriate pediatric emergency surgical care course in Uganda. J Pediatr Surg 56:811–815. https://doi.org/10.1016/j.jpedsurg.2020.10.004

128. Vakili MA, Mohjervatan A, Heydari ST, et al (2014) The efficacy of a first aid training course for drivers: an experience from northern Iran. Chin J Traumatol 17:289–292

129. Van de Velde S, De Buck E, Vandekerckhove P, Volmink J (2011) Evidence-Based African First Aid Guidelines and Training Materials. PLoS Med 8:e1001059. https://doi.org/10.1371/journal.pmed.1001059

130. Van Heng Y, Davoung C, Husum H (2008) Non-doctors as trauma surgeons? A controlled study of trauma training for non-graduate surgeons in rural Cambodia. Prehosp Disaster Med 23:483–489; discussion 490-491. https://doi.org/10.1017/s1049023x00006282

131. VanRooyen MJ, Erickson TB, Cruz C, et al (2000) Training military medics as civilian prehospital care providers in Southern Sudan. Prehosp Emerg Care 4:65–69. https://doi.org/10.1080/10903120090941687

132. Wafik W, Tork H (2014) Effectiveness of a first-aid intervention program applied by undergraduate nursing students to preparatory school children. Nurs Health Sci 16:112–118. https://doi.org/10.1111/nhs.12083

133. Wang P, Li N, Gu Y, et al (2010) Comparison of severe trauma care effect before and after advanced trauma life support training. Chin J Traumatol 13:341–344

134. Wesson HKH, Boikhutso N, Bachani AM, et al (2014) The cost of injury and trauma care in low- and middle-income countries: a review of economic evidence. Health Policy Plan 29:795–808. https://doi.org/10.1093/heapol/czt064

135. Wisborg T, Murad MK, Edvardsen O, Brinchmann BS (2008) Life or death. The social impact of paramedics and first responders in landmine-infested villages in northern Iraq. Rural Remote Health 8:816

136. Wisborg T, Murad MK, Edvardsen O, Husum H (2008) Prehospital trauma system in a low-income country: system maturation and adaptation during 8 years. J Trauma 64:1342–1348. https://doi.org/10.1097/TA.0b013e31812eed4e

137. Wu H-H, Patel KR, Caldwell AM, et al (2016) Surgical Management and Reconstruction Training (SMART) Course for International Orthopedic Surgeons. Ann Glob Health 82:652–658. https://doi.org/10.1016/j.aogh.2016.06.002

138. Xu J, You C, Zhou L, et al (2011) Long-term results of patients with head injuries treated in different hospitals after the Wenchuan, China, earthquake. World Neurosurg 75:390–396. https://doi.org/10.1016/j.wneu.2011.02.006

139. Harrison R, Jones B, Gardner P, Lawton R (2021) Quality assessment with diverse studies (QuADS): an appraisal tool for methodological and reporting quality in systematic reviews of mixed- or multi-method studies. BMC Health Serv Res 21:144. https://doi.org/10.1186/s12913-021-06122-y
